# Photochemical Probe Identification of a Small‐Molecule Inhibitor Binding Site in Hedgehog Acyltransferase (HHAT)**

**DOI:** 10.1002/anie.202014457

**Published:** 2021-05-14

**Authors:** Thomas Lanyon‐Hogg, Markus Ritzefeld, Leran Zhang, Sebastian A. Andrei, Balazs Pogranyi, Milon Mondal, Lea Sefer, Callum D. Johnston, Claire E. Coupland, Jake L. Greenfield, Joshua Newington, Matthew J. Fuchter, Anthony I. Magee, Christian Siebold, Edward W. Tate

**Affiliations:** ^1^ Department of Pharmacology University of Oxford Oxford OX1 3QT UK; ^2^ Department of Chemistry Imperial College London London W12 0BZ UK; ^3^ Division of Structural Biology Wellcome Centre for Human Genetics University of Oxford Oxford OX3 7BN UK; ^4^ National Heart & Lung Institute Imperial College London London SW7 2AZ UK

**Keywords:** enzymes, Hedgehog acyltransferase, Hedgehog signalling, membrane-bound O-acyltransferase, photoaffinity labelling

## Abstract

The mammalian membrane‐bound *O*‐acyltransferase (MBOAT) superfamily is involved in biological processes including growth, development and appetite sensing. MBOATs are attractive drug targets in cancer and obesity; however, information on the binding site and molecular mechanisms underlying small‐molecule inhibition is elusive. This study reports rational development of a photochemical probe to interrogate a novel small‐molecule inhibitor binding site in the human MBOAT Hedgehog acyltransferase (HHAT). Structure‐activity relationship investigation identified single enantiomer **IMP‐1575**, the most potent HHAT inhibitor reported to‐date, and guided design of photocrosslinking probes that maintained HHAT‐inhibitory potency. Photocrosslinking and proteomic sequencing of HHAT delivered identification of the first small‐molecule binding site in a mammalian MBOAT. Topology and homology data suggested a potential mechanism for HHAT inhibition which was confirmed by kinetic analysis. Our results provide an optimal HHAT tool inhibitor **IMP‐1575** (*K*_i_=38 nM) and a strategy for mapping small molecule interaction sites in MBOATs.

## Graphical abstract

Structure‐activity relationship analysis of Hedgehog acyltransferase (HHAT) inhibitors allowed rational design of photochemical probes for HHAT. Probe photocrosslinking identified the first small‐molecule inhibitor binding site in HHAT and revealed the inhibitory mechanism, providing an optimal HHAT tool inhibitor **IMP‐1575** (*K*_i_=38 nM) for future studies.

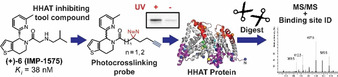

Members of the membrane‐bound *O*‐acyltransferase (MBOAT) superfamily of proteins are involved in several critically important biological pathways.^[1]^ In humans, these include Wnt acyltransferase (Porcupine; PORCN),^[2]^ Hedgehog acyltransferase (HHAT)^[3]^ and ghrelin *O*‐acyltransferase (GOAT)^[4]^ which regulate Wnt and Hedgehog signaling, and appetite sensing, respectively. These MBOATs are attractive therapeutic targets in cancer and obesity,^[1]^ and structural information for mammalian MBOATs is highly sought after. The membrane topology of various mammalian MBOATs has been experimentally determined, supporting a conserved arrangement of multiple transmembrane helices and catalytic residues.^[1a]^ Recent determination of the structure of a bacterial MBOAT, DltB, provided the first insights into MBOAT architecture and mechanism,^[5]^ revealing that DltB forms a transmembrane pore‐like structure with the active site in the centre of the pore, and providing a rationale for how MBOATs can combine substrates present on opposite sides of biological membranes.^[5]^ De novo computational predictions suggest GOAT may adopt a similar structure,^[6]^ and the first cryo‐EM structures of a human MBOAT, diacylglycerol *O*‐acyltransferase 1 (DGAT1),^[7]^ confirmed the pore‐like architecture of MBOATs and provided insights into the catalytic mechanism. However, the mechanism of small molecule MBOAT inhibition remains largely unknown.

Hedgehog signaling drives growth during development and is reactivated in certain cancers, making HHAT an attractive therapeutic target to block aberrant signaling.^[1]^ HHAT *S*‐acylates Hedgehog signaling proteins, which then undergo *S*,*N*‐acyl shift to yield the final amide‐linked product.^[3]^ HHAT predominantly attaches palmitic (C16:0) fatty acid, using palmitoyl‐Coenzyme A (Pal‐CoA) as the lipid donor substrate (Figure 1 A).^[3]^ *N*‐acylation of Sonic Hedgehog protein (SHH) by HHAT and C‐terminal auto‐*O*‐cholesterylation^[10]^ is required for signaling function. We previously determined the membrane topology of HHAT, which consists of ten transmembrane helices, two reentrant loops, and one palmitoylation‐tethered reentrant loop (Figure 1 B).^[8]^ Remarkably these experimental analyses undertaken in live cells placed the MBOAT signature residues His379 and Asp339 on opposite sides of the membrane, raising questions regarding the potential role of these residues in the catalytic mechanism. There is one known series of small‐molecule inhibitors of HHAT,^[11]^ with RUSKI‐201 (**1**, Figure 1 C) the only molecule in this class with proven on‐target cellular activity over a non‐cytotoxic concentration range.^[9]^ **1** contains two undefined stereocenters, with further scope for exploration of the structure–activity relationship (SAR) to generate higher potency inhibitors. There is also currently no information about this series’ binding mode at HHAT, or indeed the binding site of any inhibitor of a mammalian MBOAT. Photocrosslinking of ligands to proteins followed by proteomic mass spectrometry‐based sequencing is a powerful method to identify binding sites.^[12]^ However, mass spectrometry studies remain technically challenging for integral membrane proteins.^[13]^ Here, we report a SAR investigation leading to discovery of the most potent single‐enantiomer HHAT inhibitor to date, and rational design of a photochemical probe that was applied to identify the small‐molecule inhibitor binding site in HHAT, providing the first insights into the mechanism of HHAT inhibition.

**Figure 1:**
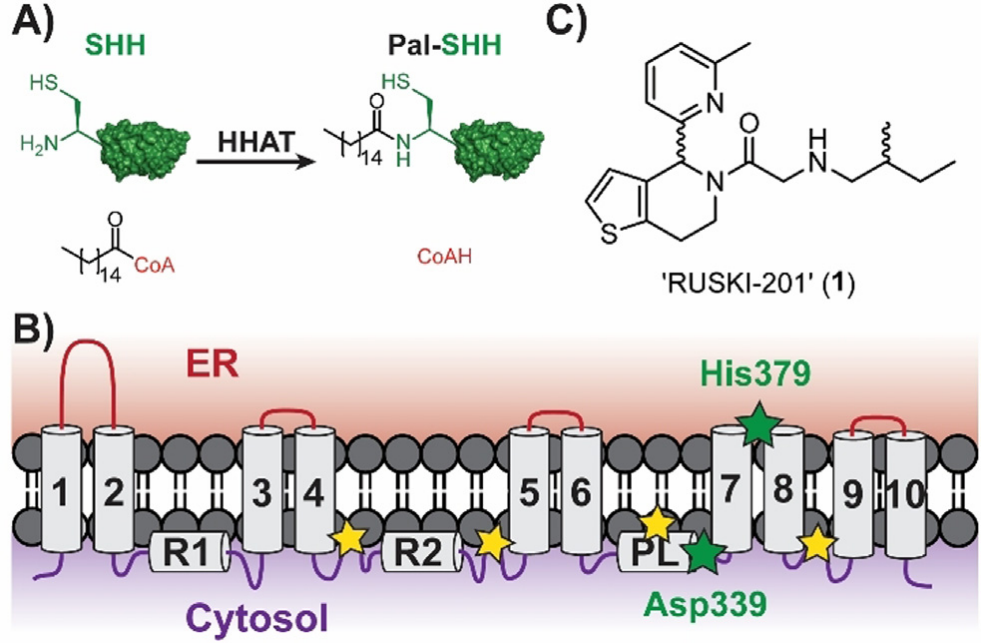
Hedgehog acyltransferase (HHAT) function and topology. A) N‐acylation reaction of Sonic Hedgehog (SHH) with palmitoyl‐CoA catalyzed by HHAT. B) Experimentally determined topology model for HHAT,^[8]^ showing ten transmembrane loops (1–10), two re‐entrant loops (R1–2) and one palmitoylation‐tethered loop (PL). Yellow stars: sites of palmitoylation; green stars: signature MBOAT residues involved in catalysis. C) Structure of RUSKI‐201, the only previously known highly selective HHAT inhibitor.^[9]^

The amide‐linked side chain of **1** was selected for SAR investigation. The 4,5,6,7‐tetrahydrothieno[3,2‐*c*]pyridine core (**2**) was synthesized by Bischler–Napieralski cyclization,^[14]^ or via a shorter and higher yielding Pictet–Spengler cyclization (Scheme S1). Acetylated derivative **3** was prepared to investigate the importance of the aminoalkyl chain. Amide‐linked sidechain derivatives **4**–**6** were prepared via *N*‐benzyl‐protected intermediates to enhance handling of the amines by decreasing volatility (Scheme S2). **1** contains two undefined stereocenters, in the central ring system and in the 2‐methylbutylamino chain. To investigate the stereochemical requirements for inhibition, (*S*)‐2‐methylbutylamine was used to prepare **4** with defined sidechain stereochemistry. 3‐Methylbutylamine (**5**) and 2‐methylpropylamine (**6**) derivatives were prepared to remove the stereocenter. To investigate the importance of the sidechain secondary amine, tertiary amine **7** and cyclic derivative **8** were prepared from *N*‐methyl isobutylamine and 3‐methyl piperidine, respectively. To further probe the role of the secondary amine, urea derivative **9** was prepared from isopentylamine (Scheme S2).

We recently reported the acylation‐coupled lipophilic induction of polarization (Acyl‐cLIP) assay, a facile and universally applicable method to monitor enzymes processing lipid post‐translational modifications which uses the hydrophobicity increase on lipidation to drive a polarized fluorescence readout.^[15]^ Dose‐response analysis of inhibition of purified HHAT was conducted using real‐time Acyl‐cLIP analysis (Table 1). **1** exhibited a half‐maximal inhibitory concentration (IC_50_) of 2.0 μM (95 % confidence interval (CI)=1.4–2.8 μM).^[15]^ Core **2** showed no inhibition, whereas acetamide **3** showed reduced activity (IC_50_=13 μM, 95 % CI=7.4–24 μM), indicating the amide is required for activity and that the aminoalkyl chain improves potency. The alkyl chain *S*‐enantiomer **4** exhibited ≈2‐fold reduced potency (IC_50_=3.7 μM, 95 % CI=1.7–8.2 μM) compared to **1**, indicating stereochemistry at this position marginally impacts activity. Moving the methyl group by one position in **5** showed a minor decrease in potency (IC_50_=5.8 μM, 95 % CI=4.3–7.7 μM), whereas shortening the alkyl chain to **6** modestly increased potency compared to **1** (IC_50_=1.3 μM, 95 % CI=0.88–2.0 μM) whilst also removing one stereocenter. Tertiary amine derivatives **7** and **8**, as well as urea analogue **9**, showed substantially reduced activity (Table 1).

**Table 1:** Structure‐activity relationship investigation of the amide substituent of **1** (RUSKI‐201). Data represent mean and 95 % confidence interval (CI, *n*=3).

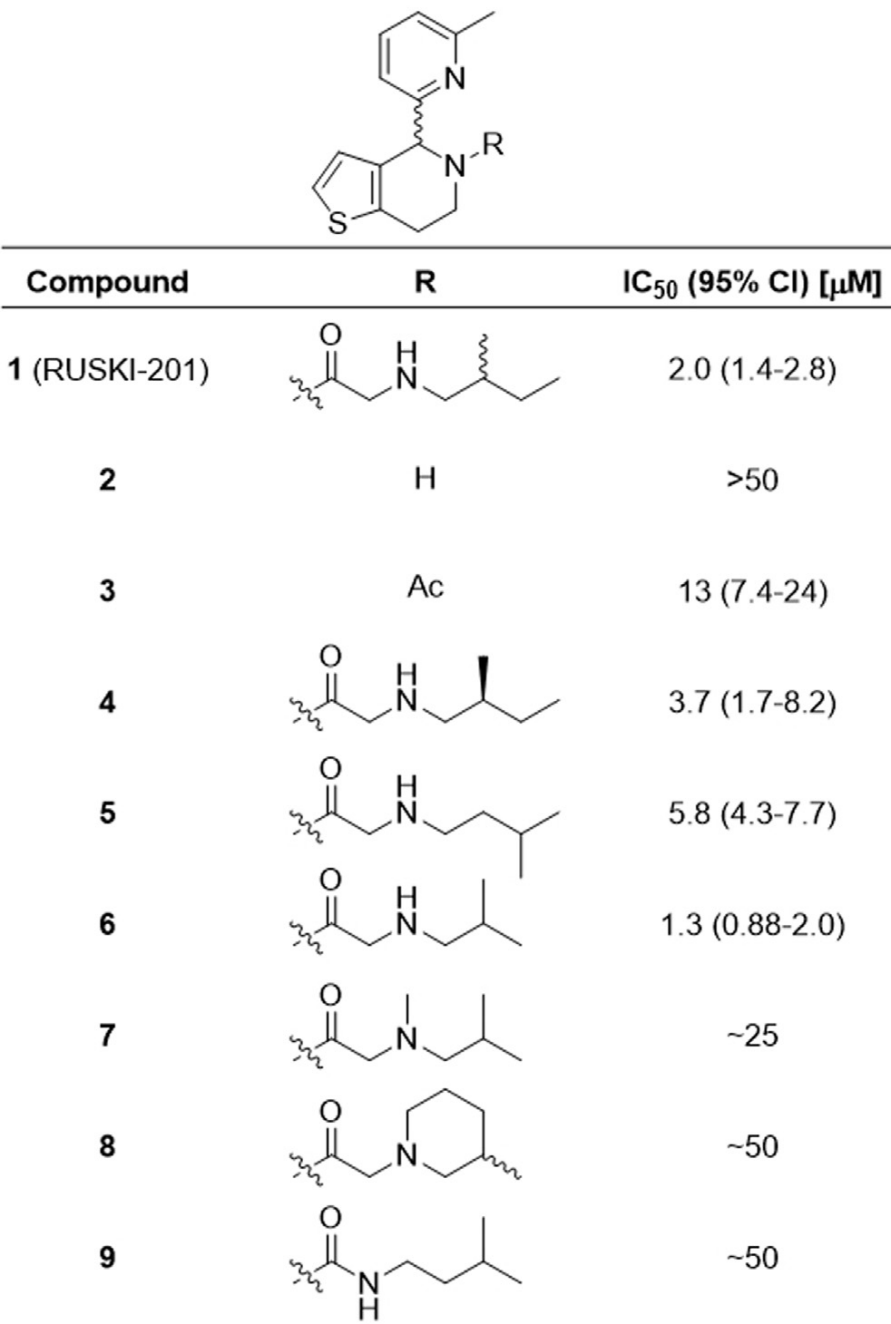

The most potent compound, **6**, contains an undefined stereocenter in the dihydrothieno[3,2‐*c*]pyridine core, and we hypothesized that one enantiomer may optimally fit the HHAT binding site. **(+/−)‐6** was therefore purified by chiral preparative HPLC to obtain **(+)‐6** and **(−)‐6** in >99:1 enantiomeric ratio (Figure S1). **(−)‐6** displayed no HHAT inhibition, whilst **(+)‐6** displayed two‐fold increased potency compared to **(+/−)‐6** (Table 2). The lead inhibitor **(+)‐6**, which we term **IMP‐1575**, is the first sub‐micromolar small‐molecule HHAT inhibitor reported to‐date (IC_50_=0.75 μM, 95 % CI=0.49–1.1 μM). **IMP‐1575** is an oil, precluding determination of absolute stereochemistry by X‐ray methods, despite extensive efforts. Circular dichroism (CD) spectra of **IMP‐1575** and **(−)‐6** were recorded (Figure S2), and compared to those predicted by three different functional/basis‐set configurations (Figure S3).^[16]^ Each of these predictions indicate that **(+)‐6** (**IMP‐1575**) is the (*R*)‐enantiomer (Figure S4). To further confirm the absolute stereochemistry of **IMP‐1575**, two enantioselective reduction methods predicted to yield **(*R*)‐2** were performed (Scheme S3).^[17]^ Asymmetric hydrogen transfer (AHT) using RuCl(*p*‐cymene)[(*R*,*R*)‐TsDPEN] generated the highest enantiomeric excess of **(+)‐2** in 92:8 *er*, and acylation of enantiomerically‐enriched **2** yielded an active HHAT inhibitor (Figure S5). Taking these data together, the stereogenic center in **(+)‐6** (**IMP‐1575**) was assigned as (*R*).

**Table 2:** Development of single‐enantiomer HHAT inhibitor **(+)‐6** (**IMP‐1575**). **(−)‐6** does not inhibit HHAT, whereas **(+)‐6** (**IMP‐1575**) is twice as potent as **(+/−)‐6**. Data represent mean and 95 % CI (*n*=3).

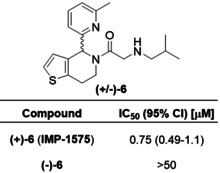

Collectively, SAR data demonstrated the amide carbonyl was critical for HHAT binding, whereas the secondary amine improved potency but was not essential and alteration of the alkyl chain was well‐tolerated. These new insights led to design of photocrosslinking chemical probes **10** and **11** to investigate the small‐molecule binding site in HHAT (Figure 2 A). In probe **10** the secondary amine moiety was exchanged for a diazirine group, and the terminal alkyl chain replaced with an alkyne; probe **11** contains an additional carbon between the carbonyl and diazirine. The diazirine allows photoactivated chemical crosslinking to nearby residues in HHAT, with the alkyne allowing biorthogonal functionalization via copper(I)‐catalyzed azide‐alkyne cycloaddition (CuAAC, “click chemistry”) for subsequent analysis. *In silico* SAR analysis using the inhibitory potency of compounds **1**–**9** as a training set predicted both probes to be active HHAT inhibitors (Figure S6). The sidechains were synthesized and coupled to **2** (Scheme S4,5). Pleasingly, probes **10** and **11** retained inhibitory activity against HHAT (IC_50_=16 μM, 95 % CI=12–20 μM and IC_50_=2.4 μM, 95 % CI=1.8–3.3 μM, respectively, Figure 2 B) demonstrating the key functional groups were tolerated. Probe photoactivation by UV irradiation (365 nm) was monitored by LC/MS, demonstrating an activation half‐life of 3.3 min for probe **10** and 32 s for **11** (Figure S7). Protein crosslinking was next investigated through CuAAC functionalization with a fluorophore and in‐gel fluorescence readout. As an integral membrane protein, HHAT presents substantial challenges in sample handling and cannot be precipitated to remove excess fluorophore which otherwise binds non‐specifically (data not shown). Fluorogenic azide CalFluor‐647 (Figure S8)^[18]^ was therefore used to reduce background fluorescence from dye that had not reacted with alkyne probe. **10** or **11** (10 μM) were incubated with purified HHAT with or without UV irradiation (1 min). Functionalization with CalFluor‐647 by CuAAC followed by in‐gel fluorescence analysis indicated crosslinking of probe **11** to HHAT promoted by UV irradiation (Figure 2 C), whereas probe **10** showed minimal crosslinking under these conditions (Figure S8). Competing probe **11** binding with 40‐fold excess **IMP‐1575** reduced crosslinking to near background levels (Figure 2 C).

**Figure 2:**
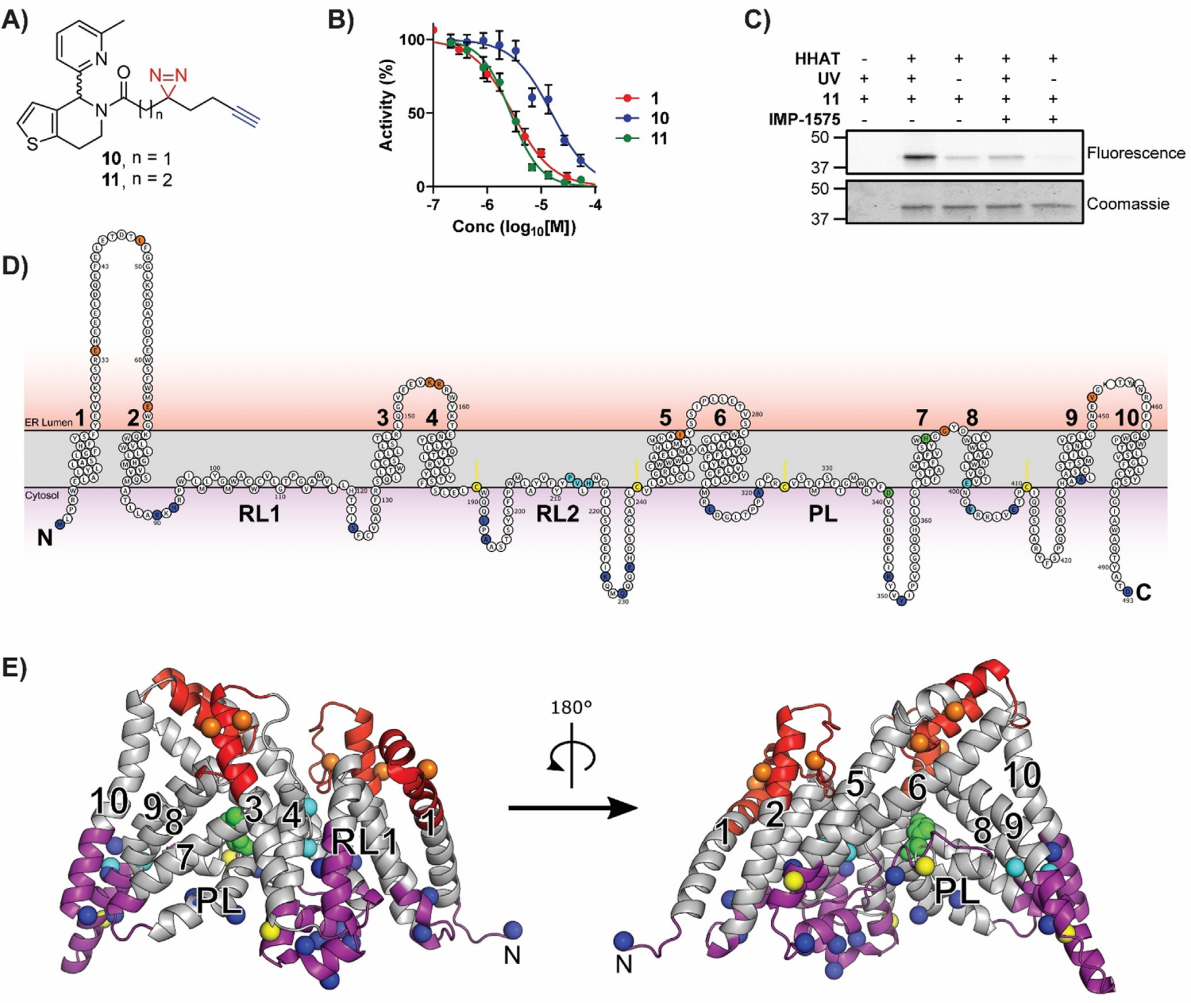
Photochemical probe identification of the small‐molecule binding site in HHAT. A) Structure of photocrosslinking probes **10** and **11**. B) Acyl‐cLIP assays showing probes are active HHAT inhibitors. Data represent mean and SEM (*n*=3). C) UV‐crosslinking (365 nm, 1 min) of **11** to purified HHAT and competition with **IMP‐1575**, analyzed by CalFluor647 functionalisation followed by in‐gel fluorescence and Coomassie staining. Image representative of two independent experiments. D) Topology model of HHAT showing cytosolic (purple) and ER lumen (red) loops. Key residues are colored as catalytic His379 and Asp339 (green), probe‐modified Pro212, Val213, His215, Glu399 and Val402 (cyan), palmitoylated cysteines (yellow), and residues that have had their topology experimentally determined as cytosolic (blue) or luminal (orange). E) Homology model of HHAT, colored as in (D), showing probe modified residues Pro212, Val213, His215 in proximity to the central catalytic site on the cytosolic face of HHAT.

Having identified suitable conditions for photocrosslinking of **11** to HHAT, identification of the small‐molecule binding site was sought through crosslinking with protease digestion and tandem LC‐MS/MS analysis. To achieve maximum sequence coverage purified HHAT (1 μg) was digested with trypsin, Glu‐C, Proteinase K, chymotrypsin, combinations of Lys‐C and trypsin, or ProteaseMAX and trypsin. Filter‐aided sample preparation (FASP)^[19]^ was used to remove *n*‐dodecyl‐β‐D‐maltopyranoside (DDM) detergent used to solubilized HHAT, and reduce and alkylate peptides prior to analysis. Moderate‐to‐low sequence coverage was achieved with all proteases (Table S1), with chymotrypsin having greatest coverage (51 %). Combined, chymotrypsin, Proteinase K (41 %), and trypsin (35 %) gave 68 % overall sequence coverage (Figure S9). Purified HHAT and **11** (25 μM) were irradiated for 3 min, followed by FASP processing and digestion with trypsin, chymotrypsin, or Proteinase K. LC‐MS/MS identified two peptides with increased mass consistent with crosslinking to **11**, and MS/MS fragmentation indicated modification of residues Pro212, Val213, His215, Glu399 and Val402 (Figure S10). Aliphatic diazirines have a general preference for crosslinking to larger polar amino acids,^[20]^ indicating the probe‐modified residues detected in HHAT may be directed by specific interactions with **11**.

To investigate how the identified binding residues may be involved in HHAT's structure and function, residue positions were compared to the experimentally determined membrane topology of HHAT (Figure 2 D).^[8]^ Probe‐binding residues are located in re‐entrant loop 2 (Pro212, Val213 and His215), which is positioned near the cytosolic face of the membrane, or immediately following transmembrane helix 8 (Glu399 and Val402), which is also positioned on the cytosolic membrane face. These residues are distant in the HHAT primary sequence from the signature MBOAT catalytic residues (Asp339 and His379), therefore a structural model for HHAT was generated based on sequence homology to DltB^[5]^ (Figure 2 E). Phyre2, SwissModel and Robetta (old and new) servers were used to generate homology models of HHAT. Models were initially screened based on agreement with previous topology data.^[8]^ Interestingly, all models shared the same fold between residues 94–206, with greater variability in the final two predicted transmembrane helices at the N‐ and C‐termini. A final model was selected based on optimal hydrophobic packing of the terminal membrane‐spanning helices (Figure 2 E). Predicted transmembrane regions from topology studies were arranged around a central pore (Figure 2 E, shown in grey). Signature MBOAT His379 was located at the center of the pore in close proximity to the catalytically essential Asp339, suggesting a central catalytic site. Inhibitor‐binding residues Pro212, Val213, and His215 were located adjacent to the proposed catalytic site, whereas residues Glu399 and Val402 were not located in proximity to the proposed catalytic site in this model (Figure 2 E).

Collectively, this analysis suggested that there may be a small‐molecule inhibitor‐binding site in HHAT located on the cytosolic side of the ER membrane in proximity to the catalytic site. As Pal‐CoA is expected to approach HHAT from the cytosolic side of the membrane, this suggested compounds from this series, such as **IMP‐1575** (Figure 3 A), may bind in competition with Pal‐CoA. **IMP‐1575** (50–0.023 μM) was therefore analyzed for effects on HHAT kinetics at varying Pal‐CoA concentrations (50–0.19 μM, Figure 3 B,C). Analysis using a mixed model equation for inhibition^[21]^ generated *α*=470 (95 % CI=320–620), indicative of a competitive mode of inhibition for **IMP‐1575** with respect to Pal‐CoA (*K*_i_=38 nM, 95 % CI=29–46 nM). Taken together, these data support identification of a small‐molecule binding site in HHAT that competes with Pal‐CoA binding to disrupt enzymatic function with high affinity.

**Figure 3:**
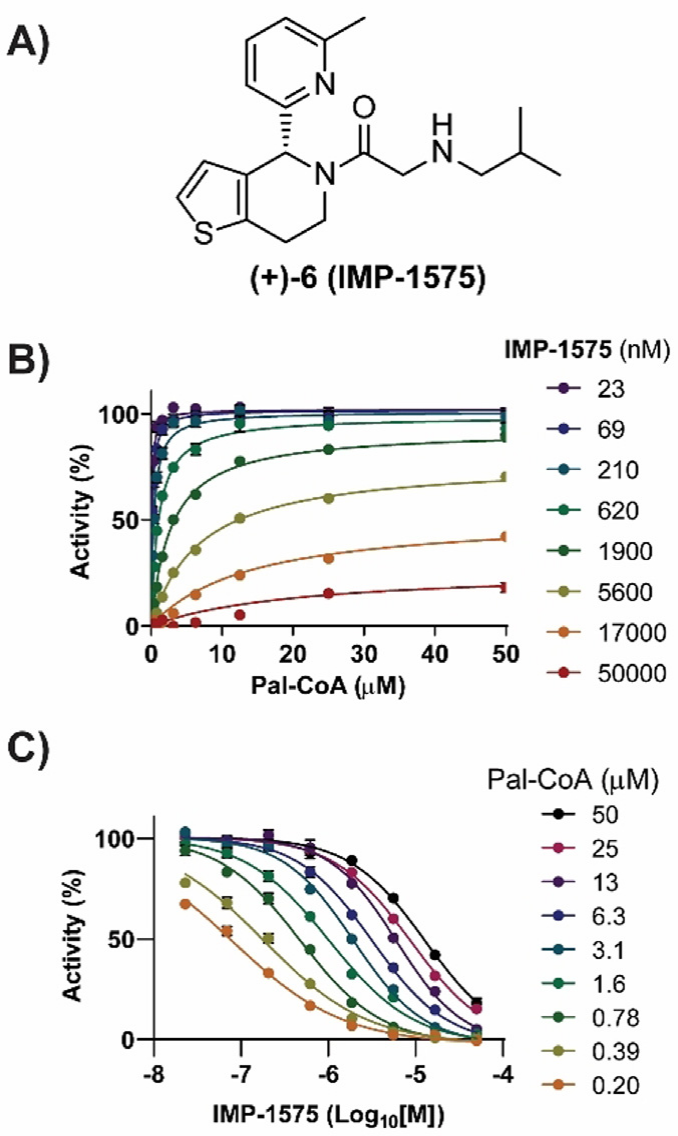
IMP‐1575 inhibition kinetics. A) Structure of **IMP‐1575**. B) Effect of IMP‐1575 inhibition on palmitoyl‐Coenzyme A (Pal‐CoA) kinetics. C) Corresponding effect of Pal‐CoA concentration on **IMP‐1575** inhibitory potency, demonstrating **IMP‐1575** is a highly potent competitive inhibitor with respect to Pal‐CoA (*K*_i_=38 nM, 95 % CI=29–46 nM). Data represent mean and SEM (*n*=3).

We present here a new paradigm in rational development of photocrosslinking chemical probes for MBOATs that can identify small‐molecule binding sites on these very challenging but important targets. Probe **11** was designed based on new SAR insights into known inhibitor **1**, which retained required HHAT inhibitory activity (Figure 2). Probe **11** was used here to identify a small‐molecule inhibitor binding site in an MBOAT for the first time. Other mammalian MBOATs, such as PORCN and GOAT, have known inhibitors and their investigation may be expedited by technical advancements reported here, including use of fluorogenic click regents for SDS‐PAGE analysis, and FASP processing in combination with multiple proteases to increase sequence coverage. Photochemical tools based on substrates may further allow mapping of key functional sites in HHAT and other MBOATs. The limitations of biochemical topology analysis and homology models for inhibitor design are significant, therefore direct structural information for MBOATs remains an important goal for future inhibitor development. We further disclose **IMP‐1575**, a single enantiomer small molecule, as the most potent HHAT inhibitor reported to date that competes with Pal‐CoA (*K*_i_=38 nM, Figure 3). Previous studies of this chemical series suggested it is noncompetitive with Pal‐CoA (for RUSKI‐43, *K*_i_=6.9 μM);^[11]^ however, these experiments did not test inhibitor concentrations below 10 μM, preventing accurate mechanistic determination. Future studies of HHAT enzymology could take advantage of **IMP‐1575**, with the inactive enantiomer serving as an ideal control. In this regard, the improved synthetic routes to HHAT inhibitors (Schemes S1–3) presented here will significantly accelerate future development of this series. In summary, we present chemical tools and methodology to provide insight into HHAT which may expedite future studies and drug discovery efforts against this important target class.

## Conflict of interest

The authors declare no conflict of interest.

## Supporting information

As a service to our authors and readers, this journal provides supporting information supplied by the authors. Such materials are peer reviewed and may be re‐organized for online delivery, but are not copy‐edited or typeset. Technical support issues arising from supporting information (other than missing files) should be addressed to the authors.

Supplementary
