# Supplementary material for: Photochemical Probe Identification of a Small‐Molecule Inhibitor Binding Site in Hedgehog Acyltransferase (HHAT)
Source: Angew Chem Int Ed Engl. 2021 May 14;60(24):13542–7. doi: 10.1002/anie.202014457 (PMC8252026; doi:10.1002/anie.202014457)
Supplement: Supplementary file 1 — Supplementary [file ANIE-60-13542-s001.pdf]

Angewandte Chemie
Eine Zeitschrift der Gesellschaft Deutscher Chemiker

Supporting Information

Photochemical Probe Identification of a Small-Molecule Inhibitor Binding Site in Hedgehog Acyltransferase (HHAT)**

Thomas Lanyon-Hogg, Markus Ritzefeld, Leran Zhang, Sebastian A. Andrei, Balazs Pogranyi, Milon Mondal, Lea Sefer, Callum D. Johnston, Claire E. Coupland, Jake L. Greenfield, Joshua Newington, Matthew J. Fuchter, Anthony I. Magee, Christian Siebold, and Edward W. Tate**

anie_202014457_sm_miscellaneous_information.pdf

Author Contributions

T.L. Conceptualization: Equal; Data curation: Lead; Formal analysis: Lead; Funding acquisition: Equal; Investigation: Supporting; Methodology: Lead; Project administration: Equal; Supervision: Equal; Writing—original draft: Lead; Writing—review & editing: Equal
M.R. Data curation: Supporting; Formal analysis: Supporting; Investigation: Supporting; Supervision: Supporting; Writing—review & editing: Supporting
L.Z. Data curation: Supporting; Formal analysis: Supporting; Investigation: Supporting; Writing—review & editing: Supporting
S.A. Data curation: Supporting; Formal analysis: Supporting; Investigation: Supporting; Writing—review & editing: Supporting
B.P. Data curation: Supporting; Formal analysis: Supporting; Investigation: Supporting; Methodology: Supporting; Writing—review & editing: Supporting
M.M. Data curation: Supporting; Formal analysis: Supporting; Investigation: Supporting; Methodology: Supporting; Writing—review & editing: Supporting
L.S. Resources: Lead; Writing—review & editing: Supporting
C.J. Data curation: Supporting; Formal analysis: Supporting; Investigation: Supporting; Writing—review & editing: Supporting
C.C. Formal analysis: Supporting; Software: Supporting; Visualization: Supporting; Writing—review & editing: Supporting
J.G. Data curation: Supporting; Formal analysis: Supporting; Writing—review & editing: Supporting
J.N. Data curation: Supporting; Visualization: Supporting; Writing—review & editing: Supporting
M.F. Formal analysis: Supporting; Supervision: Supporting; Writing—review & editing: Supporting
A.M. Conceptualization: Equal; Funding acquisition: Equal; Project administration: Equal; Supervision: Equal; Writing—review & editing: Equal
C.S. Formal analysis: Supporting; Funding acquisition: Equal; Project administration: Equal; Software: Supporting; Supervision: Equal; Visualization: Equal; Writing—review & editing: Equal
E.T. Conceptualization: Equal; Funding acquisition: Equal; Project administration: Equal; Supervision: Equal; Writing—review & editing: Equal.

Table of Contents

Supplementary Data 3

Supplementary Schemes 4

Supplementary Figures 6

Supplementary Tables 16

Materials and Methods 17

Abbreviations........ 17

General Information 17

Biological and Biochemical Methods 17

HHAT Purification 17

Acyl-cLIP Assay 17

Density-Functional Theory Modelling 18

Circular Dichroism........ 19

In Silico Bioactivity Analysis 19

Photoactivation Analysis by LC/MS........ 19

Click Chemistry and SDS-PAGE Analysis........ 19

Protease Digestion and Sequencing 20

Crosslinking for Binding Site Identification........ 20

NanoLC-MS/MS Data Acquisition 20

Peptide Search and Data Analysis 21

HHAT Modelling........ 21

Chemical Synthesis........ 21

General Information 21

Supplementary References........ 59

Supplementary Data

Supplementary Figure S1. Chiral HPLC traces for separation of (+)-6 (IMP1575) and (-)-6. . 6

Supplementary Figure S2. Molar ellipticity of (+)-6 (IMP-1575) and (-)-6. 7

Supplementary Figure S3. Geometry-optimised structures of (*R*)-6 8

Supplementary Figure S4. Experimental and theoretical circular dichroism spectra of (+)-6 (IMP-1575) and (-)-6. 9

Supplementary Figure S5. Asymmetric synthesis of (*R*)-enantiomer analogues................. 10

Supplementary Figure S6. Quantitative SAR analysis of probes 10 and 11 11

Supplementary Figure S7. Photoactivation of probes 10 and 11. 12

Supplementary Figure S8. Photocrosslinking of probes 10 and 11 to HHAT. 13

Supplementary Figure S9. Coverage of HHAT sequence. 14

Supplementary Figure S10. LC-MS/MS detection of peptides modified by probe 11. 15

Table S1. HHAT sequence coverage following protease digestion..................................... 16

Supplementary Schemes

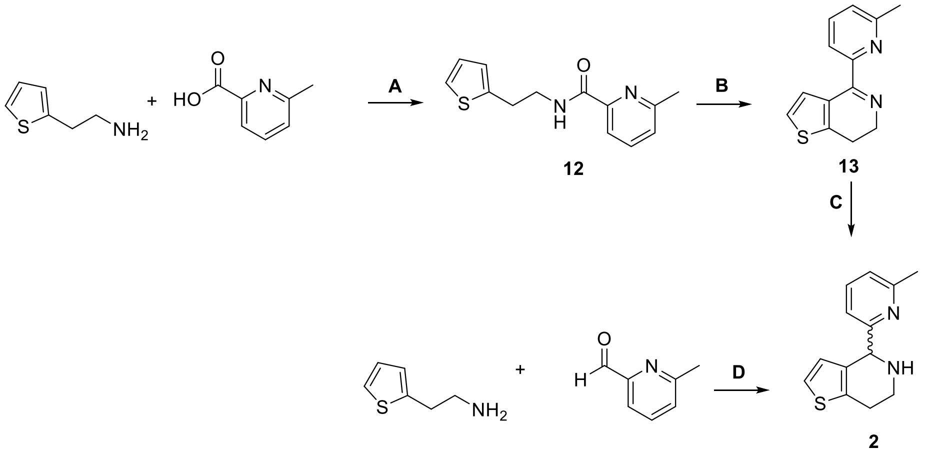

Route	2 yield (%)
A, B, C	9
D	54

Supplementary Scheme S1. Synthesis of key intermediate 2.

General procedures: **A)** PyBOP, DIPEA, DMF, room temperature, overnight; **B)** $POCl_3$, P_2O_5, toluene, microwave (140 °C); **C)** $NaBH_4$, room temperature, 1 h;[1] **D)** EtOH, TEA, room temperature, 15 h, then TFA, room temperature, 30 min. The new route for synthesis of **2** via **procedure D** is both quicker and higher yielding.

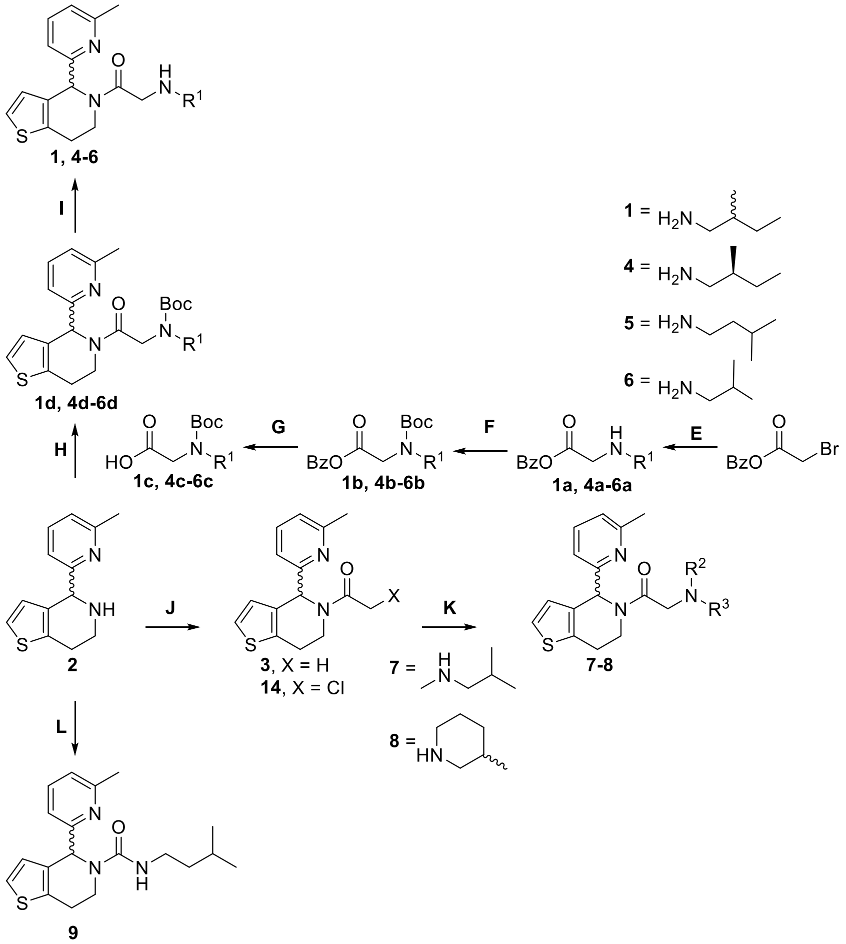

Supplementary Scheme S2. Synthesis of analogues 3-9.

General procedures: **E)** room temperature, overnight; **F)** amine, TEA, Boc_2O, 0 °C, 15 min; **G)** 10% Pd/C, NH_4HCO_3, 75 °C, 30 min; **H)** EDC, HOBt, DIPEA; **I)** TFA, room temperature, 3 h; **J)** acetyl chloride, TEA, DCM, room temperature, 2 h or chloroacetyl chloride, DIPEA, THF, 0 °C to room temperature, 1 h; **K)** amine, K_2CO_3, MeCN, 60 °C, overnight; **L)** CDI, DCM, room temperature, 1 h.

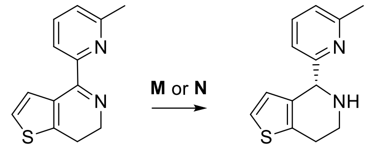

13 (+)-(*R*)-2

Supplementary Scheme S3. Asymmetric synthesis of (+)-(*R*)-2.

General procedures: **M)** 1 M HCl in TBME, then $[Ir(COD)Cl]_2$, (*S*)-P-Phos, H_3PO_4, THF, H_2, 3 bar, overnight;[2] **N)** RuCl(*p*-cymene)[(*R*,*R*)-Ts-DPEN], HCOOH:TEA (5:2), iPrOH, 30 °C, overnight.[3]

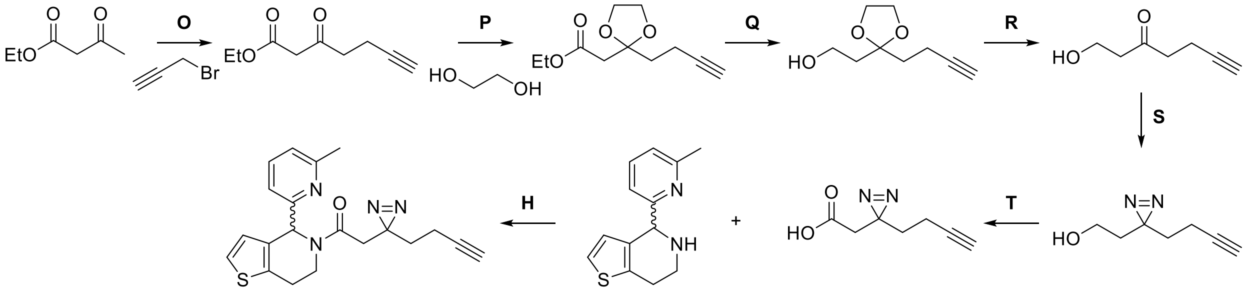

10 2

Supplementary Scheme S4. Synthesis of probe 10

General procedures: **O)** diisopropylamine, BuLi, THF, -78 °C to 0 °C; **P)** *p*-TsOH, $CH(OEt)_3$, room temperature, 36 h; **Q)** $LiAlH_4$,Et_2O, 0 °C to room temperature, 30 min; **R)** *p*-TsOH, acetone, room temperature, 4 h; **S)** 7 M NH_3, MeOH, 0 °C, 3 h, then NH_2OSO_3, MeOH, 0 °C to room temperature, overnight, then I_2, DIPEA, MeOH, 0 °C, 2 h;[4] **T)** Jones Reagent, acetone, 0 °C to room temperature, 3 h.[5]

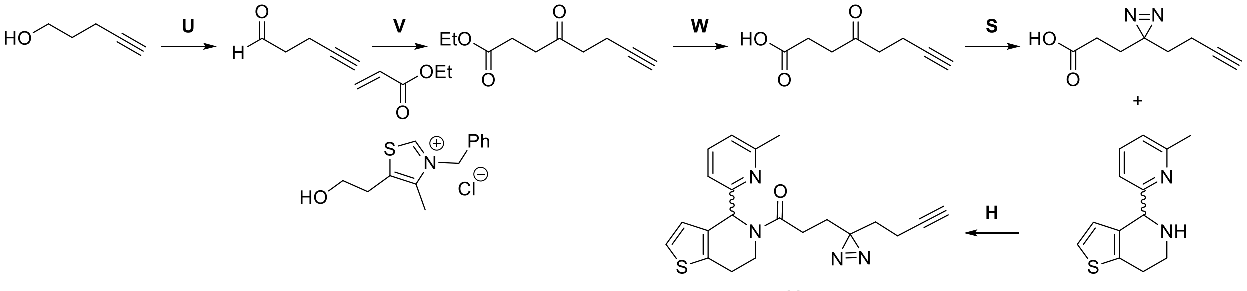

11 2

Supplementary Scheme S5. Synthesis of probe 11.

General procedures: **U)** Dess-Martin periodinane, DCM, 0 °C to room temperature, 2 h; **V)** TEA, 1,4-dioxane, 84 °C, 54 h; **W)** LiOH, water, MeOH, room temperature, overnight.[6]

Supplementary Figures

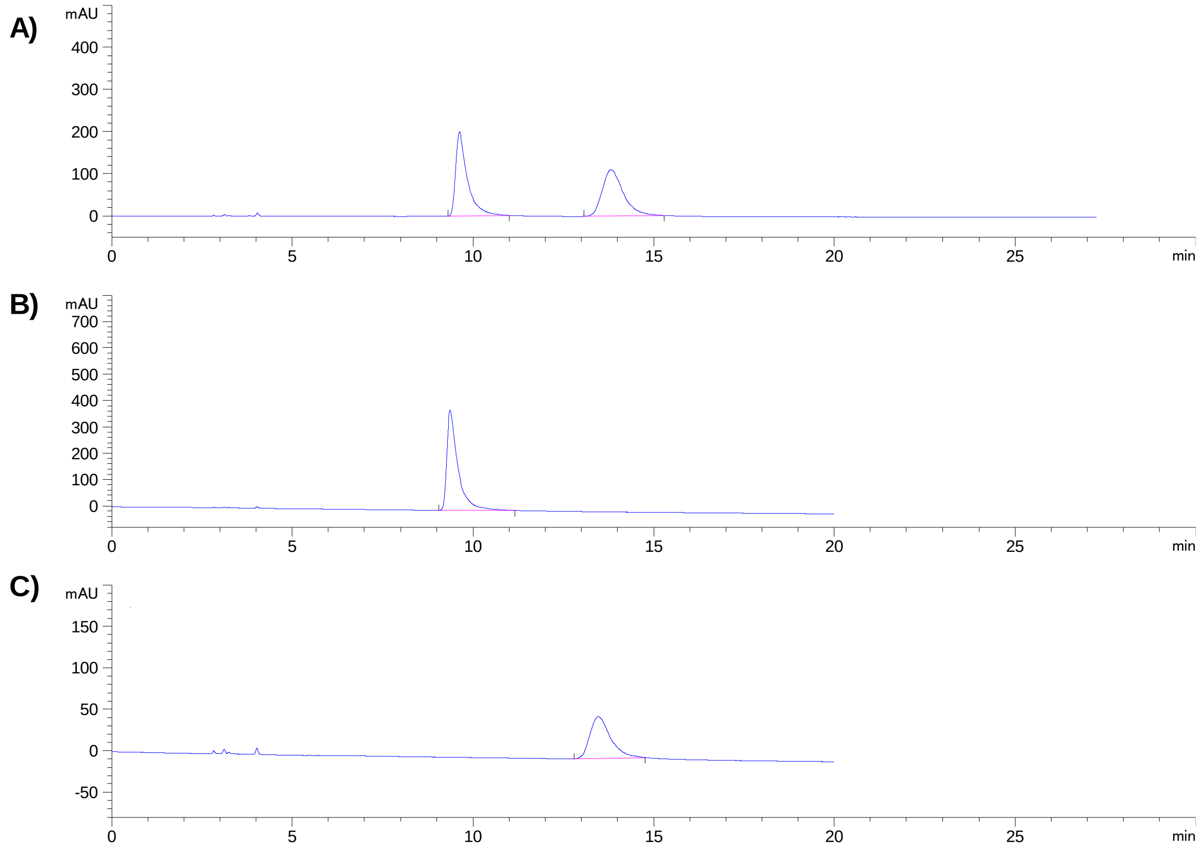

Chromatogram	Compound	Peak (min)	Area (%)
**A**	**(+/-)-6**	9.6	50
		13.8	50
**B**	**(+)-6 (IMP-1575)**	9.4	100
**C**	**(-)-6**	13.5	100

Supplementary Figure S1. Chiral HPLC traces for separation of (+)-6 (IMP1575) and (-)-6.

A) Analytical chiral HPLC trace for **(+/-)-6**. B) Analytical chiral HPLC trace for **(+)-6** (**IMP-1575**) following preparative chiral HPLC. C) Analytical chiral HPLC trace for **(-)-6** following preparative chiral HPLC.

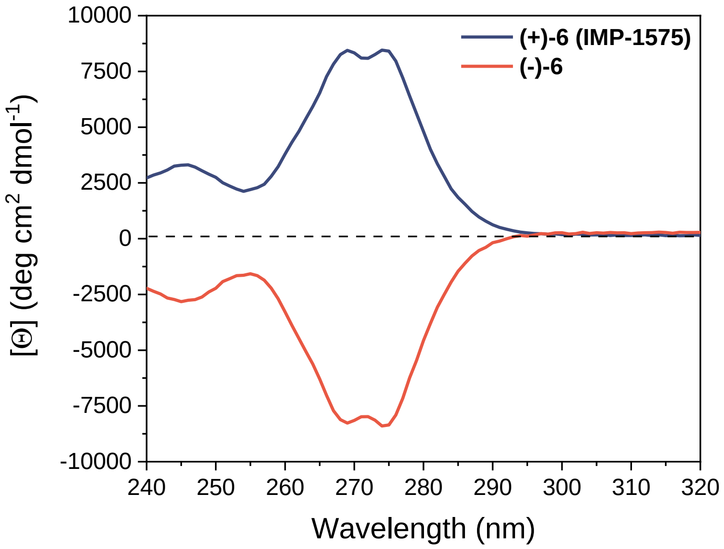

Supplementary Figure S2. Molar ellipticity of (+)-6 (IMP-1575) and (-)-6.

Spectra were recorded at 298 K in anhydrous acetonitrile at 155 µM concentration.

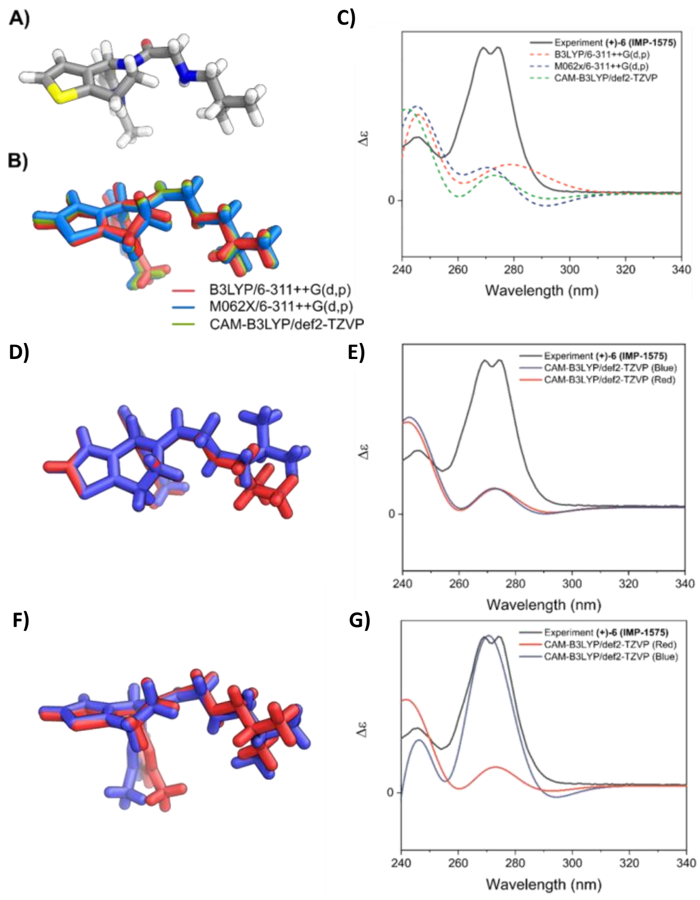

Supplementary Figure S3. Geometry-optimised structures of (*R*)-6

A) Geometry-optimised structure of (*R*)-**6** using a CAM-B3LYP functional and a def2-TZVP basis set. Acetonitrile was included in the calculation using a CPCM solvation model (carbon - grey, nitrogen - blue, oxygen – red, sulphur - yellow, hydrogen – white). B) Comparison of optimised structures of (*R*)-**6** with different functional/basis set combinations, using a CPCM solvation model for acetonitrile. C) Comparison of the predicted circular dichroism (CD) spectra using different functionals and basis-sets with the experimental spectrum of **(+)-6** (**IMP-1575**) (black). Overall, each of the different functional/basis-set combinations reproduce the general shape of the experimental CD spectrum, and, importantly, the predicted key CD bands are identical in sign. The B3LYP/6-311++G(d,p) spectrum (red) deviates from the experimental spectrum with respect to the relative energy separation of the two distinct CD bands (245 nm and 272 nm). The separation of these two CD band is better reproduced in the M062X/6-311++G(d,p) (blue). The predicted CD spectra from the range-separated CAM-B3LYP functional and def2-TZVP basis-set (green) provided a better match for the relative intensity of the two key CD bands (245 nm and 272 nm). Thus, from the combinations of functionals/basis-sets investigated, the CAM-B3LYP/def2-TZVP best describes our experimental data. D) Geometry-optimised structures of (*R*)-**6** for two different alkyl chain conformations, using a CAM-B3LYP functional and a def2-TZVP basis set, to determine if changes in the molecule's conformation can change the sign of the key CD band and hence the assignment of absolute stereochemistry. E) Comparison of the predicted CD spectra of the two alkyl chain conformations shown in (D) with the experimental spectrum of **IMP-1575** (black); minimal deviations in the predicted CD spectra are observed between conformations. F) Geometry-optimised structures of (*R*)-**6** with two different orientations of the pyridyl-ring with respect to the bicyclic system, using a CAM-B3LYP functional and a def2-TZVP basis set. G) Comparison of the predicted CD spectra of the two geometries shown in (F) with the experimental spectrum of **IMP-1575** (black); changes in the relative intensity of the two key CD bands are observed, but the sign of the predicted CD bands does not change.

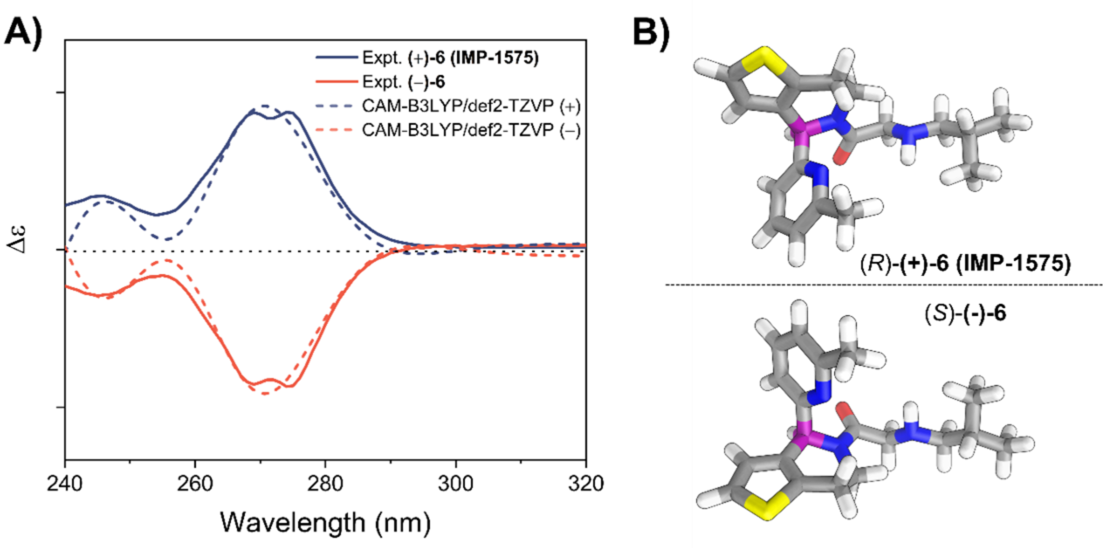

Supplementary Figure S4. Experimental and theoretical circular dichroism spectra of (+)-6 (IMP-1575) and (-)-6. A) Comparison of experimental and theoretically predicted CD spectra of **(+)-6 (IMP-1575)**. There is good agreement between the experimental and predicted spectra, with key features well reproduced at this level of theory. B) The geometry optimised structures of the two enantiomers of **6** (CAM-B3LYP/def2-TZVP) suggests that **(+)-6 (IMP-1575)** corresponds to the (*R*) enantiomer of **6**. The stereocentre has been coloured purple for clarity. It is worth noting that in all combinations of functional/basis-set investigated, the predicted CD bands possessed the same sign for a single enantiomer. Similarly, alterations in the conformation of the alkyl chain or orientation of pyridyl-moiety do not affect the sign of predicted CD bands (Figure S3), and hence the predicted absolute stereochemistry.

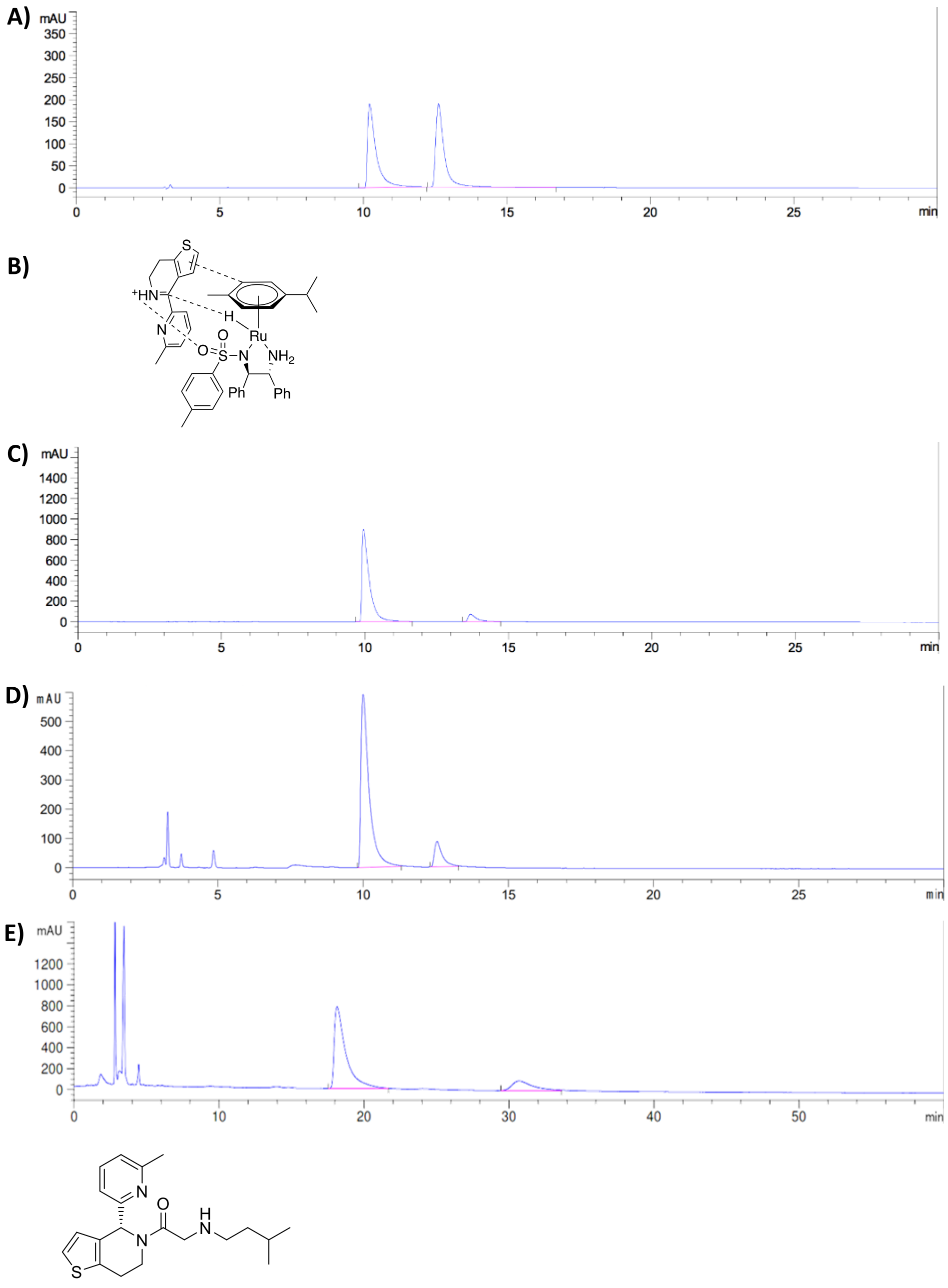

5 (83:17 er)
IC_{50} = 4.8 μM (95% CI 3.2-6.4)

Supplementary Figure S5. Asymmetric synthesis of (*R*)-enantiomer analogues

A) Analytical chiral HPLC trace for **(+/-)-2** prepared via $NaBH_4$ reduction (**procedure C**).[1] B) Proposed transition state for asymmetric hydrogen transfer (AHT) reaction. The main interactions are a CH-π interaction between cymene and thiophene and a hydrogen bond between the sulfonyl group and the iminium ion. Due to the (*R*,*R*) configuration of the TsDPEN ligand, these stabilising interactions can only occur on the *si*-face of the prochiral imine.[3] C) Analytical chiral HPLC trace for **(+)-(*R*)-2** prepared via AHT (**procedure N**) showing 92:8 *er*. D) Analytical chiral HPLC trace for **(+)-(*R*)-2** prepared via asymmetric reduction using iridium/(*S*)-P-Phos catalyst (**procedure M**) proposed to yield the *R*-enantiomer from reduction of cyclic imine **13** on the *si*-face, showing 83:17 *er*.[2] E) Acylation (**procedures H** and **I**) of the mixture enriched in **(+)-(*R*)-2** (panel D) retains inhibitory activity.

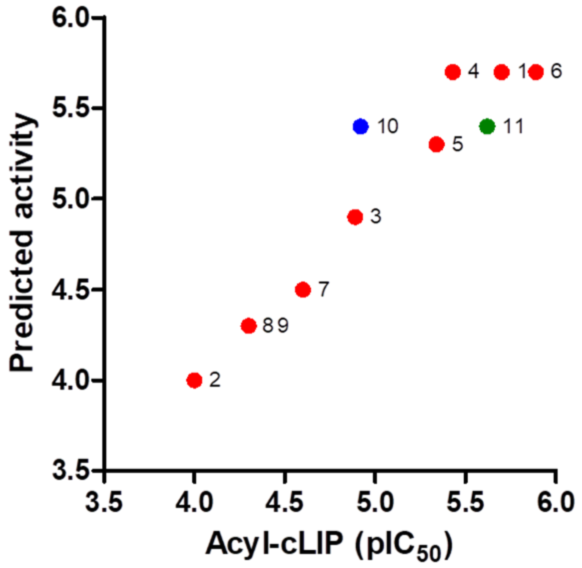

Supplementary Figure S6. Quantitative SAR analysis of probes 10 and 11

Quantitative structure-activity relationship (SAR) analysis of inhibitors in Table 1 using the software Forge™ (Cresset), which predicts probes **10** and **11** to be active HHAT inhibitors, as confirmed by Acyl-cLIP (Figure 2B).

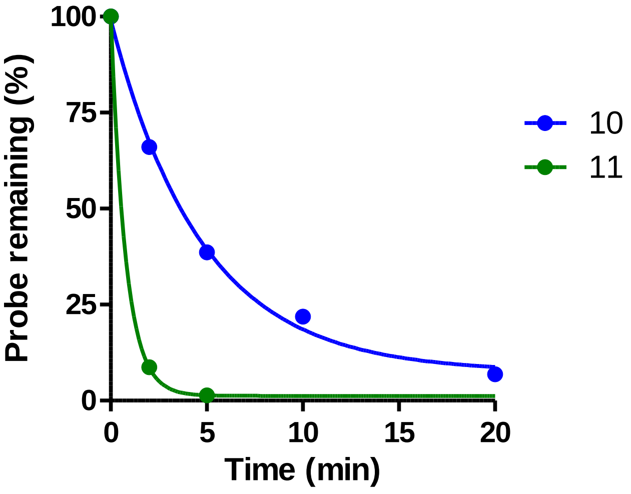

Supplementary Figure S7. Photoactivation of probes 10 and 11.

Probes **10** and **11** were UV irradiated (365 nm) and the presence of the starting material monitored by UV peak area by LC-MS. Data expressed as percentage of the probe remaining.

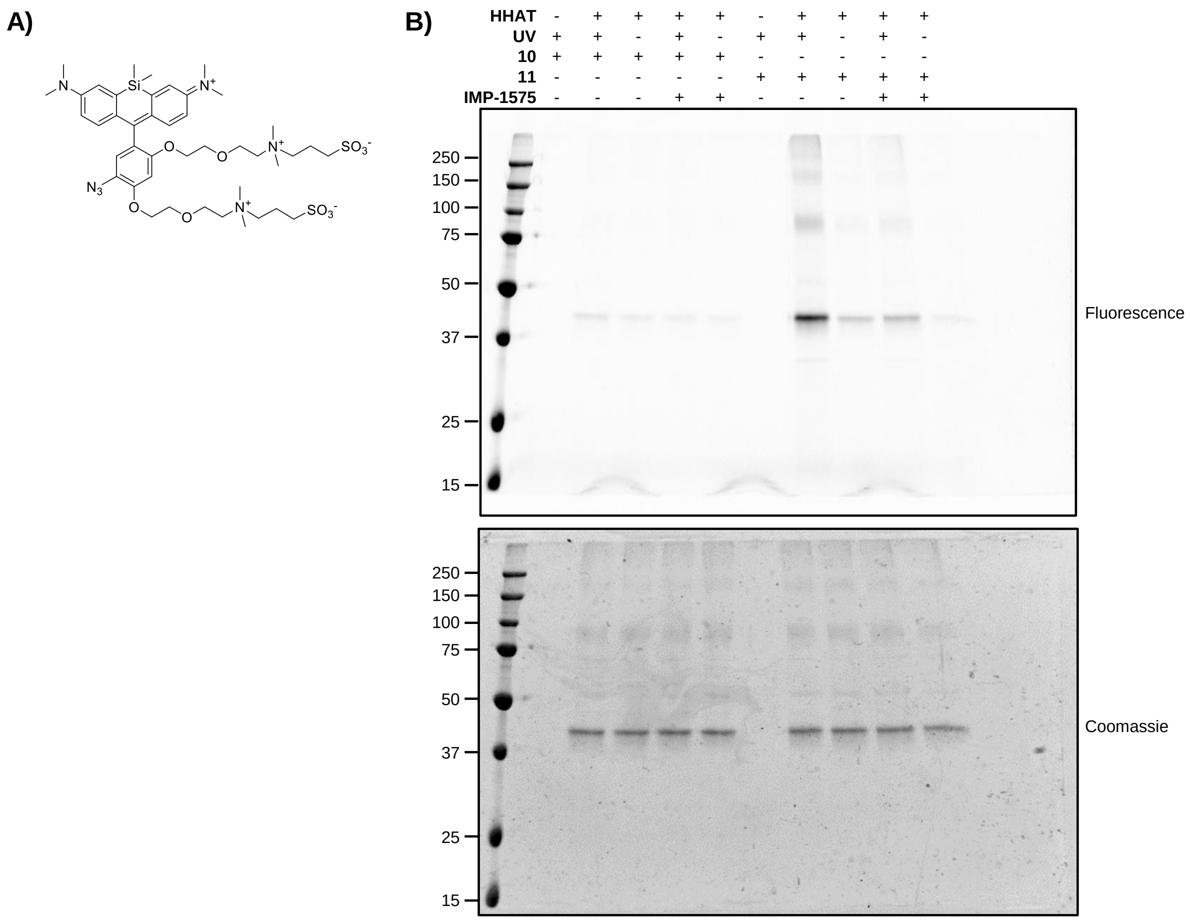

Supplementary Figure S8. Photocrosslinking of probes 10 and 11 to HHAT.

A) Structure of CalFluor647 fluorogenic dye which undergoes a turn-on in fluorescence after copper(I)-catalysed azide-alkyne cycloaddition (CuAAC).[7] B) Photocrosslinking of purified HHAT detected by click chemistry and in-gel fluorescence analysis (uncropped gel from Figure 2C). Probe **10** shows minimal labelling under these conditions. Probe **11** shows enhanced labelling with UV irradiation, which can be competed with a 40-fold excess of **IMP-1575**. Image representative of two separate experiments.

1 MLPRWELALY LLASLGFHFY SFYEVYKVSR EHEEELDQEF ELETDTLFGG LKKDATDFEW SFWMEWGKQW LVWLLLGHMV

21 VSQMATLLAR KHRPWILMLY GMWACWCVLG TPGVAMVLLH TTISFCVAQF RSQLLTWLCS LLLLSTLRLQ GVEEVKRRWY

161 KTENEYYLLQ FTLTVRCLYY TSFSLELCWQ QLPAASTSYS FPWMLAYVFY YPVLHNGPIL SFSEFIKQMQ QQEHDSLKAS

241 LCVLALGLGR LLCWWWLAEL MAHLMYMHAI YSSIPLLETV SCWTLGGLAL AQVLFFYVKY LVLFGVPALL MRLDGLTPPA

321 LPRCVSTMFS FTGMWRYFDV GLHNFLIRYV YIPVGGSQHG LLGTLFSTAM TFAFVSYWHG GYDYLWCWAA LNWLGVTVEN

401 GVRRLVETPC IQDSLARYFS PQARRRFHAA LASCSTSMLI LSNLVFLGGN EVGKTYWNRI FIQGWPWVTL SVLGFLYCYS

481 HVGIAWAQTY ATD

Supplementary Figure S9. Coverage of HHAT sequence.

Sequence coverage of HHAT after protease digestion. Underlined sequence represents detection of a peptide after digestion with trypsin (purple, 35%), chymotrypsin (yellow, 51%) or proteinase K (red, 41%). Collectively the three proteases allow detection of 68% of the amino acid sequence of HHAT. Signature MBOAT residues His379 and Asp339 are shown in green, with probe-modified residues Pro212, Val213, His215, Glu399 and Val402 shown in blue.

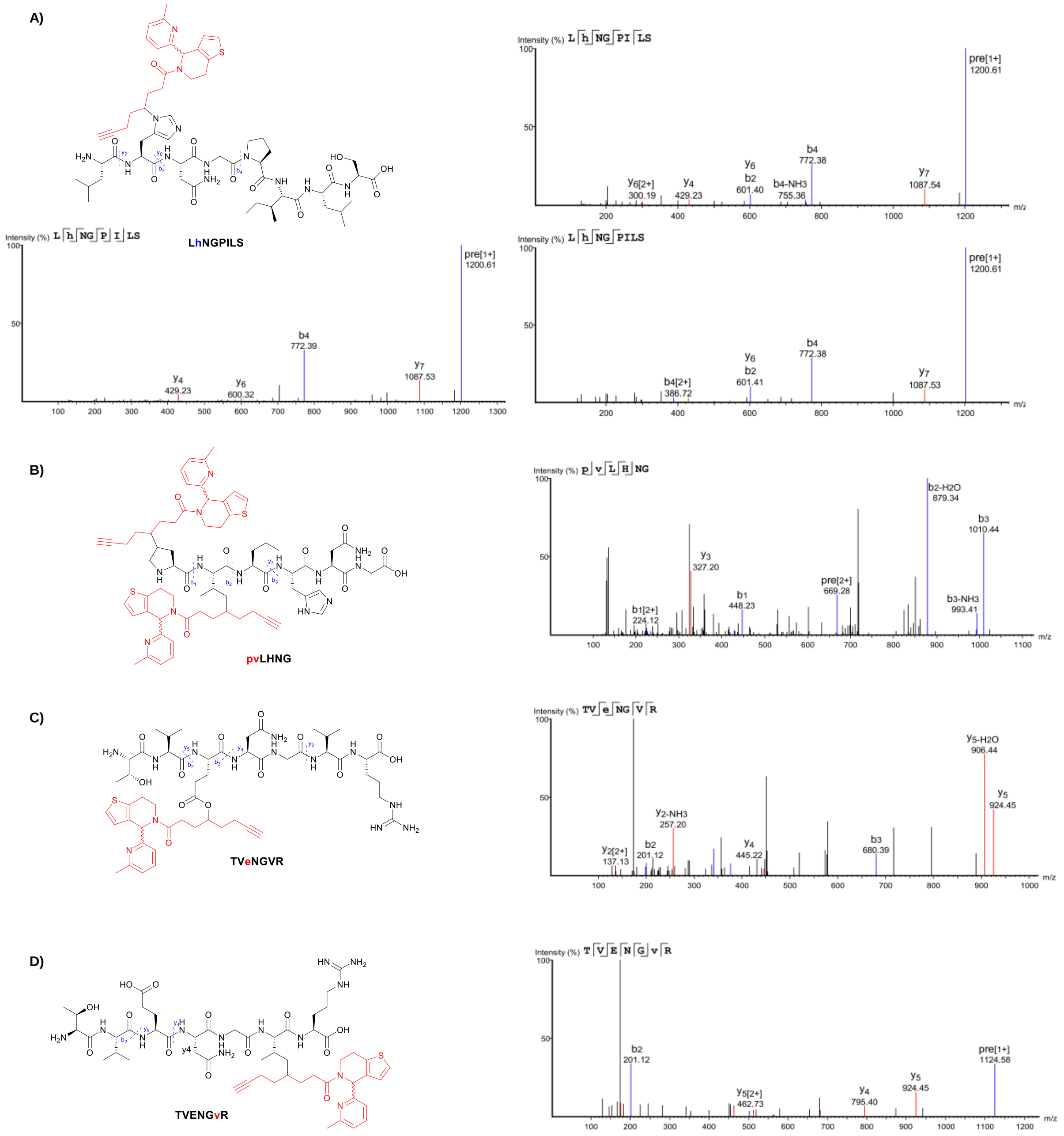

Supplementary Figure S10. LC-MS/MS detection of peptides modified by probe 11.

MS/MS fragmentation of peptides with molecular weight increases consistent with crosslinking to **11**. A) LhNGPILS for His215 modification. Calculated *m/z*: Precursor^{1+} 1200.62; y_7 1087.54; b_4 772.36; b_4-NH_3 755.33; b_2 601.30; y_6: 600.34; y_4 429.23; b_4^{2+} 386.69; y_6^{2+} 300.67. Modified fragments identified: HNGPILS, LH, LHNG. B) pvLHNG for Pro212, Val213 modification. Calculated *m/z*: Precursor^{2+} 668.82; b_3 1010.50; b_3-NH_3 993.47; b_2-H_2O 879.41; b_1 448.21; y_3: 327.14; b_1^{2+} 224.61. Modified fragments identified PV, PVL. C) TVeNGVR for Glu399 modification. Calculated *m/z*: y_5 924.44; y_5-H_2O 906.43; b_3 680.31; y_4 445.25; y_2-NH_3 257.16; y_2^{2+} 137.60; b_2 201.12. Modified fragments identified ENGVR, TVE. D) TVENGvR for Val402 modification. Calculated *m/z*: Precursor^{1+} 1124.56; y_5: 924.44; y_4 795.40;y_5^{2+} 462.73; b_2: 201.12. Modified fragments identified ENGVR and NGVR. Note, the position of modification within crosslinked residues has not been determined.

Supplementary Tables

Protease(s)	Coverage (%)
Trypsin	35
Glu-C	5
Proteinase K	41
Chymotrypsin	51
Lys-C and trypsin	36
ProteaseMAX and trypsin	35

Table S1. HHAT sequence coverage following protease digestion.

Sequence coverage following digestion of HHAT (1 µg) with either trypsin, Glu-C, Proteinase K, chymotrypsin, or combinations of Lys-C and trypsin, and ProteaseMAX and trypsin. Data represent percentage of HHAT sequence detected with a statistical significance of -LogP = 20

Materials and Methods

Abbreviations

AMBIC (ammonium bicarbonate); CDI (1,1'-carbonyldiimidazole); DCM (dichloromethane); DDM (*n*-dodecyl-β-D-maltopyranoside); DIPEA (*N*,*N*-diisopropylethylamine); DMF (dimethylformamide); DTT (dithiothreitol); EDTA (ethylenediaminetetraacetic acid); FAM (5/6-carboxyfluorescein); HEPES (4-(2-hydroxyethyl)-1-piperazineethanesulfonic acid); HOBt (hydroxybenzotriazole); TBME (*tert*-butyl methyl ether); TBTA (tris[(1-benzyl-1*H*-1,2,3-triazol-4-yl)methyl]amine); TCEP (tris(2-carboxyethyl)phosphine hydrochloride); TEA (triethylamine); TFA (trifluoroacetic acid); THF (tetrahydrofuran); TRIS (tris(hydroxymethyl)aminomethane).

General Information

All reagents were purchased from commercial sources (Sigma-Aldrich, Fisher Scientific, Acros Organics, Novabiochem) and used without further purification.

Pal-CoA (Sigma Aldrich) was stored in NaOAc (0.1 M, pH 6.0) at -80 °C. RUSKI-201 (**1**) was synthesised as previously described.[1b] The peptide comprising of residues 24-43 of SHH (residues 1-10 of the mature protein) labelled with 5/6-carboxyfluorescein (SHH-FAM) was prepared as previously described.[8]

Mixtures described as % represent v/v unless otherwise stated.

Biological and Biochemical Methods

HHAT Purification

Full-length human HHAT (UniProt Q5VTY9) C-terminally fused with a 1D4 epitope tag was expressed and purified via antibody purification with Rho-1D4 antibody (University of British Columbia) coupled to CNBr-activated sepharose beads (GE Healthcare) as previously described.[9] Purified HHAT 0.1-0.2 mg/mL in storage buffer (10 mM HEPES, 150 mM NaCl, 5% glycerol, 0.025% (w/v) DDM, 0.0006% (w/v) cholesterol-hemisuccinate, pH 7.5) was flash-frozen in liquid nitrogen and stored at -80 °C.

Acyl-cLIP Assay

The Acylation-coupled Lipophilic Induction of Polarisation (Acyl-cLIP) was performed as previously described,[9] using Rho-1D4 antibody purified HHAT. Briefly, HHAT was diluted to 45 µg/mL in solubilisation buffer (20 mM HEPES, 350 mM NaCl, 1% (w/v) DDM, 5% glycerol, pH 7.3) added to SHH-FAM (2 µM) in reaction buffer (100 mM MES, 20 mM NaCl, 1 mM DTT, 1 mM TCEP, 0.1% (w/v) BSA, pH 6.5) in a 1:10 (v/v) ratio, respectively. The HHAT/SHH-FAM solution (12 µL/well) was dispensed into a black 384-well plate (Corning, 3575). The reaction was initiated by addition of Pal-CoA (7.5 µM) in reaction buffer (8 µL/well), and fluorescence anisotropy measured every 1 min for 1 h on an EnVision Xcite 2104 (PerkinElmer). Measurement used a FITC FP D505fp mirror module, FITC FP 480 nm excitation filter (30 nm bandwidth), FITC FP P-pol 535 nm first emission filter (40 nm bandwidth), and FITC FP S-pol

535 nm second emission filter (40 nm bandwidth), measurement height = 9.52 mm, 8 flashes/well, PMT gain = 319, and G-factor = 1.09. Fluorescence anisotropy was calculated using equation 1:

$$(1)\ FA = \frac{S - GP}{S + 2GP}$$

Where S is signal in the S-channel, P is signal in the P-channel, and G is the G-factor.

Assay data were evaluated using Microsoft Excel 2013 and GraphPad Prism 5.0. Initial rate constants were determined by linear regression, normalized to DMSO negative controls and background corrected to positive controls with solubilization buffer instead of HHAT. The half-maximal inhibitory concentration (IC_{50}) was calculated using a 4-parameter sigmoidal dose-response curve fit. Determination of the mechanism of inhibition of IMP-1575 relative to Pal-CoA was performed by fitting equation 2 for a mixed model of inhibition[10] using nonlinear regression:

$$(2)\ v = \frac{\frac{V_{max}}{\left(1+\frac{[I]}{\alpha K_i}\right)}[S]}{[S] + K_M\left(\frac{\left(1+\frac{[I]}{K_i}\right)}{\left(1+\frac{[I]}{\alpha K_i}\right)}\right)}$$

Where v is the observed rate of reaction, V_{max} is the maximum rate, [I] is the inhibitor concentration, [S] is the substrate concentration, K_m is the substrate concentration at ½ V_{max}, K_i is the affinity of the inhibitor for the free enzyme, and α is the ratio of the affinity of the inhibitor for the enzyme-substrate complex (K_i') to K_i (typically noted as K_i'/K_i).

Density-Functional Theory Modelling

Density-functional theory (DFT) predictions were performed using Gaussian 16[11] and Orca (version 4.0.1.2).[12] The compound **6** structure was initially created using Avogadro software (1.2.0) and geometry-optimised using an MMFF94s force field.[13] Avogadro was also employed to explore conformers of the structure using a “systematic rotor search”. DFT geometry optimisations were initially carried out on the MMFF94s optimised structures using either the 6-31G basis set (for the B3LYP and M062X functionals) or the def2/SVP basis set (for the CAM-B3LYP functional). Structures were then optimised with a higher-level basis set (6-311++G(d,p), for the B3LYP and M062X functional, and def2/TZVP for the CAM-B3LYP functional). Solvation was included in these calculations using a CPCM solvation model for acetonitrile. Ground state structures were confirmed by checking for the absence of imaginary vibrational modes in frequency calculations performed at the same level of theory.

The predicted CD spectra were visualised using SpecDis software.[14] The energy of the predicted CD peaks were corrected from systematic error in the calculations by performing a counter-shift corrections; CD peaks were generated using a sigma of 0.3 eV.

Circular Dichroism

Solutions for circular dichroism (CD) measurements were prepared in anhydrous acetonitrile. CD measurements were performed on a Chirascan V100 CD spectrometer (Applied Photophysics) using a 10 mm pathlength quartz cuvette. Spectra were recorded at 298 K, maintained with a Peltier temperature control. Background subtraction for each measurement was performed using data obtained from anhydrous acetonitrile in an identical cuvette. The sample concentrations were adjusted to maintain a gain voltage below 700 V. A sample integration time of 4 s was used, data were smoothed using a 5 pts Savitzky-Golay algorithm and the residuals were checked for distortions.

In Silico Bioactivity Analysis

SAR analysis and prediction of bioactivity for probes **10** and **11** was performed in Forge™ 10.6.0 (Cresset®, Litlington, Cambridgeshire, UK).[15] **IMP-1575** was used as the Reference Molecule for alignment. Compounds **1**-**9** (*R*)-enantiomers and IC_{50} values from the Acyl-cLIP assay were used as a Training Set. For the purpose of analysis of weakly active inhibitors, IC_{50} values were assigned as **2** = 100 µM, **7** = 25 µM, **8** = 50 µM, and **9** = 50 µM. Conformation Hunt was set to Normal, Alignment set to Normal, and Build Model set to Field QSAR. Probes **10** and **11** were used as a Prediction Set.

Photoactivation Analysis by LC/MS

A solution of **10** or **11** (100 µM) in water was cooled on ice and irradiated using a custom-made UV light source (365 nm, 25 mW/cm^2) for the indicated times. LC-MS analysis was performed on a Waters preparative HPLC-MS equipped with a Waters XBridge C18 4.6 × 100 mm column with 1.2 mL/min flow rate, 5-98% MeOH in water gradient over 18 min. All eluents were supplemented with 0.1% formic acid.

Click Chemistry and SDS-PAGE Analysis

All steps were performed in the dark in a cold room (4 °C), until specified otherwise. Purified HHAT was diluted to 0.02 mg/mL in solubilisation buffer and 20 uL dispensed into amber, UV-protective microfuge tubes (Eppendorf). **IMP-1575** (20 mM in 50% DMSO, 50% reaction buffer) or DMSO control was added (0.42 µL, final assay concentration 400 µM) and incubated for 15 min. Probe **10** or **11** (50 µM in 50% DMSO, 50% reaction buffer) was then added (0.42 µL, final assay concentration 10 µM) and incubated for 15 min. The required samples were then UV irradiated individually for 1 min by placing tubes with an open lid centred underneath the UV-light source. After irradiation, 1.0 µL of “click mix” (8 µL 50 mM $CuSO_4$ in water, 8 µL 50 mM TCEP in water, 4 µL 10 mM TBTA in DMSO, 4 µL 10 mM CalFluor-647 in DMSO) was added to each sample. 15 µL of each sample was transferred to a fresh amber microfuge tube, which was then vortexed at room temperature for 1 h covered under tinfoil. The click reaction was quenched by adding 0.3 µL 500 mM EDTA solution (final concentration 10 mM) and vortexing at room temperature for 5 min. 4× SDS-PAGE loading buffer (5.1 µL) was added and samples loaded onto a 10% acrylamide gel without boiling. The gel was run until the loading dye had eluted from the gel (typically 55 min at 200 V) and the in-gel fluorescence

recorded (Excitation: 635 nm Emission: 669 nm) on a Typhoon FLA 9500 (GE Healthcare). The gel was then stained using InstantBlue™ protein stain (Abcam) and imaged on an ImageQuant LAS 4000 (GE Healthcare).

Protease Digestion and Sequencing

HHAT (10 µL, 0.1 mg/mL) was diluted 1:10 in proteomics sample buffer (100 mM MES, 20 mM NaCl, 1 mM TCEP, 1 mM DTT, 0.1% (w/v) DDM, pH 6.5), transferred to a 10 kDa molecular weight cut-off (MWCO) Vivaspin 500 concentrator (Sartorius) and incubated for 15 min on ice. Urea solution (200 µL, 8 M urea in 100 mM Tris, pH 8.5, kept on ice) was added and the samples were centrifuged (14,000 g, 10 min, 4 °C). Iodoacetamide (500 mM, kept on ice) was added to a final concentration of 50 mM and the mixture was vortexed for 1 min before incubation in the dark for 20 min. The concentrators were washed with urea solution (3 × 100 µL) and with AMBIC solution (50 mM NH_4HCO_3, pH 8.5, 3 × 100 µL) via centrifugation (10,000 g, 3 min, 4 °C).

Digestions were performed using (1) sequencing grade modified trypsin (Promega) (1:10, 0.5 µL of 0.2 µg/µL stock); (2) Glu-C (Promega) (1:20, 0.5 µL of 0.1 µg/µL stock); (3) Proteinase K (New England Biolabs) (1:20, 2.5 µL of 20 ng/µL stock); (4) chymotrypsin (Promega) (1:20, 0.5 µL of 0.1 µg/µL stock); (5) Lys-C (1:10, 0.5 µL of 0.2 µg/µL stock) and trypsin (1:10, 0.5 µL of 0.2 µg/µL stock); (6) Protease MAX (1:10, 1 µL of 0.1 µg/µL stock) and trypsin (1:10, 0.5 µL of 0.2 µg/µL stock). All samples were shaken overnight at 37 °C except for chymotrypsin (25 °C, overnight). Digested peptides were collected into a clean Vivacon tube with 40 µL washes of AMBIC solution (50 mM) and NaCl (0.5 M).

Samples were acidified with 0.5% TFA and loaded onto stage tips containing three SDB-XC poly(styrenedivinyl-benzene) copolymer discs (Merck). The stage tipping procedure was performed as described previously.[16] Peptides were eluted in 80% acetonitrile in water and the solvent removed using a Savant SPD1010 SpeedVac® Concentrator at 45 °C.

Peptide samples were dissolved in LC-MS grade water containing 2% acetonitrile and 0.5% TFA (15 µL) for nano-LC-MS/MS

Crosslinking for Binding Site Identification

HHAT (100 µL, 0.01 mg/mL) in proteomics sample buffer was incubated with photochemical probe **11** (25 µM) on ice for 15 min in 10 kDa MWCO Vivaspin 500 concentrator (Sartorius). The samples were irradiated for 3 min as previously described. Samples were prepared for nanoLC-MS/MS analysis by protease digestion as previously described.

NanoLC-MS/MS Data Acquisition

Peptide samples (3 µL) were separated on an EASY-Spray™ Acclaim PepMap C18 column (50 cm × 75 µm inner diameter, 3 µm particle size, Thermo Fisher Scientific) using a 2 h linear gradient separation of 0-100% solvent B (80% acetonitrile supplemented with 0.1% formic acid): solvent A (2% acetonitrile supplemented with 0.1% formic acid) at a flow rate of 250 nL/min. The liquid chromatograph was coupled to a Q Exactive mass spectrometer via an

easy-spray ion source which was operated in data-dependent mode with survey scans acquired at a resolution of 70,000 at m/z = 200. Scans were acquired from 350 to 1650 m/z. Up to 10 of the most abundant isotope patterns with charge +2 or higher from the survey scan were selected with an isolation window of 1.6 m/z and fragmented by higher-energy collisional dissociation with normalized collision energy of 25. The maximum ion injection times for the survey scan and the MS/MS scans (acquired with a resolution of 35,000 at m/z = 200) were 20 and 120 ms, respectively. The ion target value for MS was set to 10^6 and for MS/MS to 10^5, and the intensity threshold was set to 8.3×10^2.

Peptide Search and Data Analysis

Raw files were analysed in PEAKS Studio8.5 and searched against the HHAT sequence. Cysteine carbamidomethylation, methionine oxidation and N-terminus acetylation were selected as variable modifications. Probe modification was set as fixed modification for crosslinking experiments. Up to two missed cleavages were allowed. The –10logP value was set to ≥20 for peptides. Other parameters were used as pre-set in the software (precursor mass error tolerance 15 ppm, fragment mass error tolerance 0.5 Da, precursor mass type monoisotopic, maximum variable PTM per peptide 3, maximum missed cleavage 2, and non-specific cleavage 1).

HHAT Modelling

A set of homology models for human HHAT (Uniprot ID: Q5VTY9; Genbank ID: NM_001122834.3, natural variant VAR_024743) were built using the Robetta Web Server (http://old.robetta.org/) based on the structure of the bacterial MBOAT member DltB (PDB ID: 6BUH).[17] A final model was selected based on agreement with topological data.[18]

Chemical Synthesis

General Information

Analytical chiral HPLC was performed on an Agilent 1260 Infinity Series equipped with a CHIRALPAK-IA 4.6 mm × 250 mm (eluent: hexane:propan-2-ol 90:10 or 80:20; flow rate 1 mL/min, Method A). Preparative chiral HPLC was performed on an Agilent 1200 Series equipped with a CHIRALPAK-IF 250 mm x 20 mm column (eluent: isocratic hexane:propan-2-ol 90:10, flow rate 18 mL/min, Method B).

Synthetic procedures

Procedures A-C (synthesis of 2)

Procedures A-C were performed as previously described.[1]

Procedure D (synthesis of 2)

To a stirred solution of 6-methylpicolinaldehyde (1 eq) in ethanol (3.5 mL) was added 2-(thiophen-2-yl)ethan-1-amine (1 eq) and TEA (0.2 mL) and the solution stirred for 15 h. The solvent was removed under reduced pressure and the residue was dissolved in TFA (15 mL)

and stirred at room temperature for 30 min. The solvent was removed under reduced pressure and the residue was dissolved in DCM, washed with 2 N NaOH (aq), and the aqueous layer extracted with DCM. The organics were combined, dried over $MgSO_4$, filtered and concentrated under reduced pressure to afford a brown oil which was recrystallised from DCM/hexane.

Procedure E (side chain synthesis, first step)

Benzyl bromoacetate (1 eq) was added dropwise to the corresponding primary amine (3 eq) in dry THF at 0 °C under a nitrogen atmosphere. The solution was stirred overnight at room temperature. The solvent was removed under reduced pressure and the residue purified by column chromatography.

Procedure F (side chain synthesis, Boc protection)

TEA (1.5 eq) was added to the secondary amine (1 eq) dissolved in dry methanol at 0 °C. Subsequently, di-*tert*-butyl dicarbonate (1 eq) dissolved in dry methanol was added and the mixture was stirred under a nitrogen atmosphere at 0 °C for 15 min. The solvent was removed under reduced pressure and the residue purified by column chromatography.

Procedure G (side chain synthesis, benzyl deprotection)

The Boc protected side chain (1 eq) was dissolved in dry methanol under a nitrogen atmosphere. 10% Pd/C (0.3 eq) was added followed by ammonium formate (3 eq) and the mixture heated at 75 °C for 30 min. Subsequently, the mixture was cooled to room temperature and the catalyst removed by filtration through a celite pad. The filter cake was washed with methanol and chloroform and the wash fractions were combined with the filtrate. The solvent was removed under reduced pressure and the residue dissolved in DCM and washed with 1 N HCl (aq). The organic layer was dried over $MgSO_4$ and concentrated under reduced pressure.

Procedure H (side chain coupling to 2)

The side chain acid (1 eq), HOBt (1 eq), and 1-ethyl-3-(3-dimethylaminopropyl)carbodiimide (EDC, 1.5 eq) were dissolved in DMF (5 mL) and the reaction mixture was stirred at room temperature for 30 min. Subsequently, **2** (1 eq) and *N*,*N*-diisopropylethylamine (4 eq) were added and the mixture stirred over night at room temperature. The mixture was diluted with DCM (8 mL), washed with water, 5% (w/v) LiCl (aq), and brine. The organic layer was dried over $MgSO_4$, concentrated under reduced pressure and the residue purified by column chromatography.

Procedure I (Boc deprotection)

The Boc protected intermediate was dissolved in DCM (5 mL), TFA (5 mL) was added and the resultant solution was stirred for 3 h at room temperature. The solvent was removed under reduced pressure, neutralised with saturated $NaHCO_3$ (aq) and extracted with DCM. The organic layer was dried over $MgSO_4$ and concentrated under reduced pressure. The final compounds were either used without further purification or purified by column chromatography.

Procedure I (Boc deprotection variant b)

The Boc protected intermediate was dissolved in DCM (5 mL), TFA (5 mL) was added and the resultant solution was stirred for 3 h at room temperature. The deprotected amine was purified using a strong cation exchange (SCX) column. After addition of the reaction mixture, the column was washed with methanol (4 column volumes (CV)), followed by elution with 7 N ammonia in methanol (3 CV). The eluted fractions were concentrated under reduced pressure to yield the final compound.

Procedure J (acid chloride coupling to 2)

To a mixture of **2** (1 eq) and TEA (6 eq) in dry DCM was added acetyl chloride (1.5 eq). The reaction was stirred at room temperature for 2 h. Subsequently, the solvent was removed under reduced pressure and the residue purified by column chromatography.

Procedure K (tertiary amine analogue synthesis)

To a stirred solution of **2** (100 mg, 0.43 mmol) in THF (4 mL) at 0 °C was added DIPEA (150 µL, 0.86 mmol). Chloroacetyl chloride (350 µL, 0.43 mmol) was added dropwise and the reaction allowed to warm to room temperature and stirred for 1 h. The reaction mixture was diluted in DCM and washed with water and brine. The organic layer was dried over $MgSO_4$, filtered and the solvent was removed under reduced pressure to afford a brown oil. The crude material (1 eq crude yield) was taken forward without further purification. A suspension of potassium carbonate (3 eq) and the required amine (1.1 eq) in acetonitrile (2 mL) was added. The reaction mixture was stirred at 60 °C for 16 h. The solvent was removed under reduced pressure and the residue dissolved in EtOAc then washed with water. The organic layer was dried over $MgSO_4$, filtered and the solvent removed under reduced pressure. The residue was dissolved in methanol and purified using an SCX column, eluting with 2 N ammonia in methanol. The solvent was removed under reduced pressure and the residue purified by column chromatography.

Procedure L (urea synthesis)

The primary amine (1 eq) was added to a solution of CDI (1.5 eq) in DCM (1 mL) and the mixture stirred at room temperature for 1 h. **2** (1.3 eq) was added and the reaction mixture was stirred at room temperature overnight. The solvent was removed under reduced pressure and the residue purified by column chromatography.

Procedure M (asymmetric reduction using iridium/(*S*)-P-Phos catalyst)

The imine obtained from **procedure B** (1 eq) was added to a solution of HCl (1 M) in dry TBME (2 mL), and the solvent removed under reduced pressure to afford the HCl salt. Anhydrous THF (5 mL) was added to a Parr Shaker flask containing the HCl salt, $[Ir(COD)Cl]_2$ (0.01 eq) and (*S*)-P-Phos (0.02 eq). H_3PO_4 (85% in water, 2.4 eq) was added dropwise to the mixture. The reaction mixture was purged with hydrogen three times and shaken at 3 bar pressure at room temperature overnight. The solvent was removed under reduced pressure and redissolved in DCM (10 mL). Water (5 mL) and aqueous NH_4OH (25% w/v, 6 mL) were added, and the aqueous layer extracted with DCM. The organic phase was dried over $MgSO_4$ and

filtered. The solvent was removed under reduced pressure and the residue purified by column chromatography (EtOAc:TEA 100:1).

Procedure N (asymmetric hydrogen transfer (ATH))

The imine obtained from **procedure B** (1 eq) and RuCl(*p*-cymene)[(*R*,*R*)-Ts-DPEN] (0.01 eq) were added to an argon-flushed vial, with a septum and argon balloon. Dry iPrOH (2 mL) was added, followed by HCOOH:TEA (5:2, 6.3 eq with respect to HCOOH). The reaction mixture was stirred at 30 °C overnight. Saturated Na_2CO_3 (3 mL) was added followed by stirring for 10 min. The mixture was extracted with DCM three times. The organic layer was washed with water, brine, dried over $MgSO_4$ and filtered. The solvent was removed under reduced pressure and the residue purified by column chromatography. (EtOAc:TEA 100:1).

Synthesised Compounds

It should be noted that acylated derivatives of 4,5,6,7-tetrahydrothieno[3,2-*c*]pyridines display two amide rotameric confirmations in NMR spectra.[1]

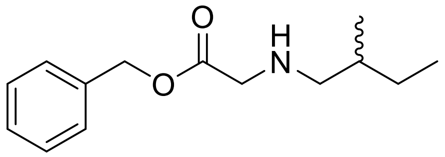

Benzyl [(2-methylbutyl)amino]acetate (1a). Compound **1a** was prepared from benzyl bromoacetate (2.8 mmol, 0.44 mL) and 2-methylbutylamine (8.4 mmol, 1.0 mL) using **procedure E** to afford the title compound as a light orange oil (2.2 mmol, 520 mg, 79%). R_f = 0.50 (n-hexane/ EtOAc/NEt_3 50:50:1). ^{1}H-NMR (400 MHz, $CDCl_3$) δ (ppm): 7.37-7.34 (m, 5H), 5.17 (s, 2H), 3.45 (s, 2H), 2.51 (dd, *J* = 11 Hz, *J* = 6.0 Hz, 1H), 2.38 (dd, *J* = 11 Hz, *J* = 7.2 Hz, 1H), 1.53-1.36 (m, 2H), 1.20-1.09 (m, 1H), 0.91-0.83 (m, 6H). ^{13}C-NMR (101 MHz, $CDCl_3$) δ (ppm): 172.6, 135.7, 128.6, 128.4, 66.5, 55.7, 51.3, 35.0, 27.4, 17.6,11.3. HRMS (ESI): calculated for $C_{14}H_{22}NO_2$ $[M+H]^+$: 236.1651, found: 236.1662.

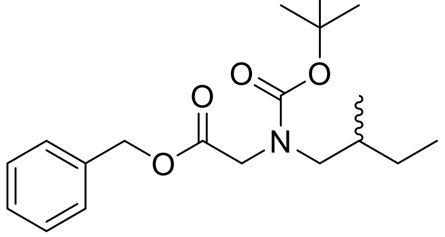

Benzyl [(*N-tert*-butoxycarbonyl)(2-methylbutyl)amino]acetate (1b). Compound **1b** was prepared from **1a** (1.5 mmol, 330 mg), TEA (2.2 mmol, 0.31 mL), and di-*tert*-butyl dicarbonate (1.5 mmol, 320 mg) using **procedure F** to afford the title compound as an orange oil (1.2 mmol, 390 mg, 81%). R_f = 0.44 (n-hexane/EtOAc 1:4). ^{1}H-NMR (400 MHz, $CDCl_3$) δ(ppm): 7.37-7.33 (m, 5H), 5.17 (s, 2H), 4.04-3.94 (m, 1H), 3.89 (s, 1H), 3.21-2.99 (m, 2H), 1.61-1.55 (m, 2H), 1.46-1.35 (m, 10H), 1.13-1.03 (m, 1H), 0.89-8.84 (m, 6H). ^{13}C-NMR (101 MHz, $CDCl_3$) δ (ppm):

170.0, 135.5, 128.6, 80.1, 66.7, 54.4, 50.0, 34.3, 28.4, 17.0, 11.3. HRMS (ESI): calculated for $C_{19}H_{30}NO_4$ $[M+H]^+$: 336.2175, found: 336.2167.

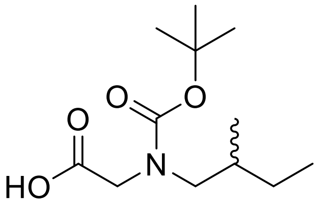

[(*N*-*tert*-Butoxycarbonyl)(2-methylbutyl)amino]acetic acid (1c). Compound **1c** was prepared from **1b** (1.7 mmol, 580 mg), 10% Pd/C (0.10 mmol, 110 mg) and ammonium formate (5.3 mmol, 330 mg) using **procedure G** to afford the title compound as a light brown oil (1.7 mmol, 410 mg, 98%). Characterisation data was consistent with previous reports.[1b]

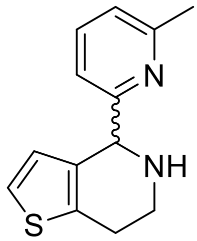

4-(6-Methylpyridin-2-yl)-4,5,6,7-tetrahydrothieno[3,2-*c*]pyridine (2). Compound **2** was prepared as reported[1b] or using **procedure D** from 6-methylpicolinaldehyde (3.5 mmol, 430 mg) to afford the title compound as a brown solid (1.9 mmol, 440 mg, 54% yield). Characterisation data were consistent with previous reports.[1b]

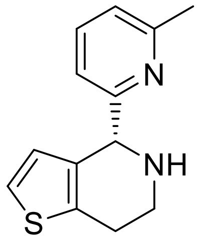

(*R*)-4-(6-methylpyridin-2-yl)-4,5,6,7-tetrahydrothieno[3,2-*c*]pyridine ((*R*)-2). The (*R*)-enriched cyclic amine **(*R*)-2** was obtained from **13** (0.15 mmol, 34 mg) using **procedure M** as a yellow oil (0.07 mmol, 17 mg, 47%, *er* 87:13), or from **13** (0.56 mmol, 128 mg) using **procedure N** as a brown oil (0.39 mmol, 90 mg, 70%, *er* 92:8, $[\alpha]^{23}_{D}$ (c = 0.8, $CHCl_3$): +26.8). Characterisation data were consistent with previous reports.[1b]

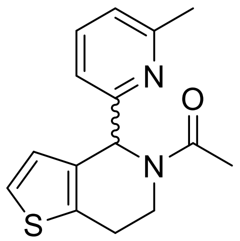

1-(4-(6-Methylpyridin-2-yl)-6,7-dihydrothieno[3,2-*c*]pyridin-5(4H)-yl)ethan-1-one (3). Compound **3** was prepared from **2** (0.089 mmol, 20 mg) using **procedure J** to afford the title compound as a yellow oil (0.062 mmol, 17 mg, 95%). R_f = 0.53 (EtOAc) ^{1}H-NMR (500 MHz, $CDCl_3$) δ (ppm): 7.56-7.47 (m, 1H), 7.18-7.12 (m, 1H), 7.10-7.04 (m, 1H), 7.03-6.94 (m, 1H), 6.92-6.80 (m, 1H), 6.63 (s, 1H), 6.03 (s, 1H), 5.03-4.95 (m, 1H), 4.09-3.97 (m, 1H), 3.08-2.82 (m, 2H), 2.56 (s, 2H), 2.51 (s, 1H), 2.35 (s, 3H), 2.22 (s, 3H). ^{13}C-NMR (125 MHz, $CDCl_3$) δ (ppm): 170.8, 159.1, 159.0, 158.6, 135.5, 133.1, 132.8, 126.7, 126.4, 123.2, 122.8, 122.3, 119.0, 117.9, 60.5, 60.4, 55.9, 42.5, 36.6, 29.7, 25.7, 24.9, 24.5, 22.6, 22.2, 21.1, 14.2. HRMS (ESI): calculated for $C_{15}H_{17}N_2O$ $[M+H]^+$: 273.1056, found: 273.1064.

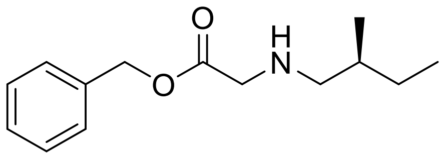

Benzyl [(*S*)-(2-methylbutyl)amino]acetate (4a). Compound **4a** was prepared from benzyl bromoacetate (2.8 mmol, 0.44 mL) and (*S*)-2-methylbutylamine (8.4 mmol, 1.0 mL) using **procedure E** to afford the title compound as a colourless oil (2.6 mmol, 620 mg, 93%). R_f = 0.25 (n-hexane/EtOAc/NEt_3 70:30:1). ^{1}H-NMR (400 MHz, $CDCl_3$) δ (ppm): 7.37-7.32 (m, 5H), 5.17 (s, 2H), 3.45 (s, 2H), 2.54-2.36 (m, 2H), 1.56-1.37 (m, 12H), 1.20-1.09 (m, 1H), 0.91-0.88 (m, 6H). ^{13}C-NMR (101 MHz, $CDCl_3$) δ (ppm): 192.9, 172.6, 135.7, 128.6, 128.4, 66.5, 55.7, 51.3, 35.0, 27.4, 17.6, 11.3. HRMS (ESI): calculated for $C_{14}H_{22}NO_2$ $[M+H]^+$: 236.1651, found: 236.1666.

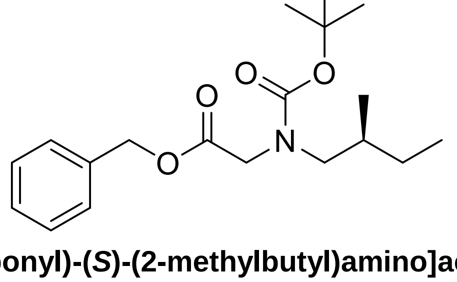

Benzyl [(*N*-*tert*-butoxycarbonyl)-(*S*)-(2-methylbutyl)amino]acetate (4b). Compound **4b** was prepared from **4a** (2.2 mmol, 520 mg), TEA (3.30 mmol, 0.46 mL) and di-*tert*-butyl dicarbonate (2.2 mmol, 480 mg) using **procedure F** to afford the title compound as a colourless oil (1.9 mmol, 620 mg, 85%). R_f = 0.45 (n-hexane/EtOAc 1:4). ^{1}H-NMR (400 MHz, $CDCl_3$) δ(ppm): 7.42-7.31 (m, 5H), 5.19 (d, J = 1.7 Hz, 2H), 4.09-3.75 (m, 2H), 3.26-2.99 (m, 2H), 1.67-1.56 (m, 2H), 1.51-1.33 (m, 9H), 1.07-1.05 (m, 1H), 0.96-0.84 (m, 6H). ^{13}C-NMR

(101 MHz, $CDCl_3$) δ (ppm): 128.6, 128.5, 128.4, 128.3,128.2, 66.7, 66.7, 54.4, 54.4, 50.0, 49.4, 34.3, 34.0, 28.4, 28.3, 28.2, 26.9, 17.0, 16.8, 11.3. HRMS (ESI): calculated for $C_{19}H_{30}NO_4$ $[M+H]^+$: 336.2178, found: 336.2178.

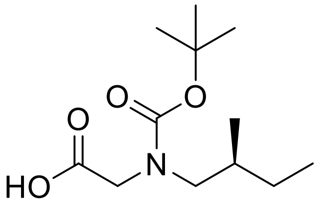

[(*N*-*tert*-Butoxycarbonyl)-(*S*)-(2-methylbutyl)amino]acetic acid (4c). Compound **4c** was prepared from **4b** (1.9 mmol, 620 mg), 10% Pd/C (0.23 mmol, 240 mg) and ammonium formate (4.6 mmol, 290 mg) using **procedure G** to afford the title compound as a brown oil (1.9 mmol, 460 mg, 100%). ^{1}H-NMR (400 MHz, $CDCl_3$) δ (ppm): 4.04-3.82 (m, 2H), 3.30-2.89 (m, 2H), 1.66-1.52 (m, 1H), 1.52-1.22 (m, 10H), 1.10 (m, 1H), 0.88 (q, *J* = 6.6, 5.8 Hz, 6H). ^{13}C-NMR (101 MHz, $CDCl_3$) δ (ppm): 54.6, 54.2, 49.5, 34.2, 34.0, 28.3, 28.2, 26.9, 17.0, 16.8, 11.3. HRMS (ESI): calculated for $C_{12}H_{24}NO_4$ $[M+H]^+$: 246.1705, found: 246.1693.

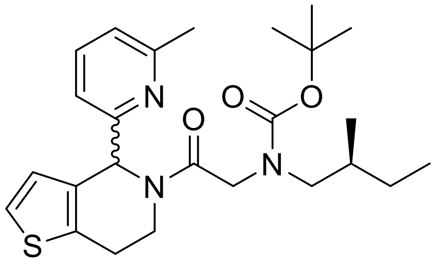

***tert*-Butyl (*S*)-(2-methylbutyl)(2-(4-(6-methylpyridin-2-yl)-6,7-dihydrothieno[3,2-*c*]pyridin-5(4*H*)-yl)-2-oxoethyl)carbamate** (**4d**). The Boc-protected intermediate **4d** was prepared **4c** (0.15 mmol, 37 mg) and **2** (0.18 mmol, 42 mg) using **procedure H** to afford the title compound as a colourless oil (0.12 mmol, 57 mg, 80%). R_f = 0.3 (n-hexane/EtOAc 3:7). ^{1}H-NMR (400 MHz, $CDCl_3$) δ (ppm): 7.48 (q, *J* = 7.6 Hz, 1H), 7.23-7.11 (m, 1H), 7.10-6.95 (m, 1H), 6.92-6.74 (m, 2H), 6.53 (s, 1H), 5.98 (d, *J* = 15 Hz, 1H), 4.95 (d, *J* = 11 Hz, 1H), 4.88 (d, *J* = 11 Hz, 1H), 4.74-4.47 (m, 1H), 4.43-4.21 (m, 1H), 4.16-3.94 (m, 1H), 3.43 (dd, *J* = 15, 6.6 Hz, 1H), 3.31-2.72 (m, 4H), 2.60-2.41 (m, 3H), 1.53-1.30 (m, 9H), 1.16-0.98 (m, 1H), 0.94-0.75 (m, 6H). HRMS (ESI): calculated for $C_{25}H_{36}N_3O_3S$ $[M+H]^+$: 458.2477, found: 458.2490.

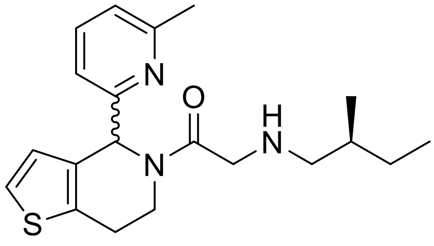

2-(*S*)-(2-Methylbutylamino)-1-[4-(6-methylpyridin-2-yl)-6,7-dihydro-4*H*-thieno[3,2-*c*]pyridin-5-yl]ethanone (**4**). Compound **4** was prepared from the Boc-protected intermediate

4d (0.11 mmol, 52 mg) using **procedure I** to afford the title compound as a yellow oil (0.90 mmol, 32 mg, 79%). R_f = 0.17 (EtOAc). Chiral HPLC (**Method A**): RT = 9.1 min (50%), 12.7 min (50%). ^{1}H-NMR (500 MHz, $CDCl_3$) δ (ppm): 7.49 (q, *J* = 7.4 Hz, 1H), 7.14 (d, *J* = 5.8 Hz, 1H), 7.09-6.94 (m, 1H), 6.92-6.77 (m, 1H), 6.56 (s, 1H), 5.98 (s, 1H), 4.94 (dd, *J* = 12, 4.0 Hz, 1H), 4.06-3.97 (m, 1H), 3.71 (dd, *J* = 16, 5.4 Hz, 1H), 3.56 (qd, *J* = 16, 5.7 Hz, 1H), 3.09-2.79 (m, 1H), 2.58-2.49 (m, 1H), 2.47-2.39 (m, 2H), 1.61-1.49 (m, 1H), 1.48-1.36 (m, 1H), 1.14 (m, 1H), 0.94-0.82 (m, 6H). ^{13}C-NMR (125 MHz, $CDCl_3$) δ (ppm): 171.1, 169.9, 159.0, 158.6, 158.4, 136.8, 136.6, 135.5, 134.1, 132.9, 132.3, 126.6, 126.2, 123.3, 122.8, 122.3, 121.9, 118.8, 118.2, 58.8, 56.4, 56.3, 56.1, 51.4, 51.2, 40.6, 36.8, 35.0, 35.0, 34.8, 27.4, 27.4, 27.3, 25.6, 24.8, 24.6, 24.4, 17.6, 17.5, 11.3, 11.2, 11.2. HRMS (ESI): calculated for $C_{20}H_{28}N_3OS$ [M+H]$^+$: 358.1953, found: 358.1960.

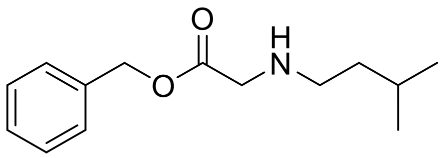

Benzyl [(3-methylbutyl)amino]acetate (5a). Compound **5a** was prepared from benzyl bromoacetate (2.8 mmol, 0.44 mL) and isopentylamine (8.4 mmol, 1.0 mL) using **procedure E** to afford the title compound as a colourless oil (0.73 mmol, 170 mg, 25%). R_f = 0.27 (n-hexane/ EtOAc/NEt_3 70:30:1). ^{1}H-NMR (400 MHz, $CDCl_3$) δ (ppm): 7.41-7.29 (m, 5H), 5.17 (s, 2H), 3.46 (s, 2H), 2.65-2.54 (m, 2H), 1.63 (m, 1H), 1.43-1.31 (m, 2H), 0.89 (d, *J* = 6.8 Hz, 6H). ^{13}C-NMR (101 MHz, $CDCl_3$) δ (ppm): 172.5, 135.6, 128.6, 128.4, 66.5, 51.0, 47.7, 39.1, 26.0, 22.3. HRMS (ESI): calculated for: $C_{14}H_{22}NO_2$ [M+H]$^+$: 236.1651, found: 236.1653.

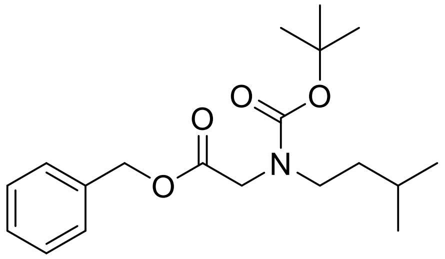

Benzyl [(*N*-*tert*-butoxycarbonyl)(3-methylbutyl)amino]acetate (5b). Compound **5b** was prepared from **5a** (0.73 mmol, 170 mg), TEA (1.1 mmol, 0.16 mL) and di-*tert*-butyl dicarbonate (0.73 mmol, 160 mg) using **procedure F** to afford the title compound as a colourless oil (0.53 mmol, 180 mg, 73%). R_f = 0.40 (n-hexane/EtOAc 1:4). ^{1}H-NMR (400 MHz, $CDCl_3$) δ(ppm): 7.35 (s, 5H), 5.17 (s, 2H), 3.99 (s, 1H), 3.88 (s, 1H), 3.27 (dt, *J* = 21, 7.7 Hz, 2H), 1.61-1.33 (m, 11H), 0.89 (d, *J* = 6.5 Hz, 7H). ^{13}C-NMR (101 MHz, $CDCl_3$) δ (ppm): 170.0, 128.6, 128.5, 128.4, 128.3, 80.0, 66.7, 49.3, 48.7, 46.8, 37.3, 36.9, 28.4, 28.2, 27.4, 25.9, 25.6, 22.5. HRMS (ESI): calculated for $C_{19}H_{30}NO_4$ [M+H]$^+$: 336.2175, found: 336.2180.

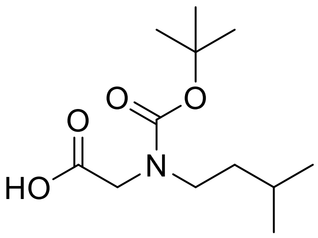

[(*N*-*tert*-Butoxycarbonyl)(3-methylbutyl)amino]acetic acid (5c). Compound **5c** was prepared from **5b** (0.52 mmol, 180 mg), 10% Pd/C (0.16 mmol, 100 mg) and ammonium formate (3.1 mmol, 190 mg) using **procedure G** to afford the title compound as a brown oil (0.46 mmol, 110 mg, 88%). ^{1}H-NMR (400 MHz, $CDCl_3$) δ (ppm): 3.97 (s, 1H), 3.90 (s, 1H), 3.28 (m, 2H), 1.63-1.29 (m, 12H), 0.91 (d, *J* = 6.6 Hz, 6H). ^{13}C-NMR (101 MHz, $CDCl_3$) δ (ppm): 77.3, 77.0, 76.7, 49.1, 47.1, 37.2, 28.3, 25.6, 22.5. HRMS (ESI): calculated for $C_{12}H_{22}NO_4$ $[M+H]^+$: 244.1549, found: 244.1553.

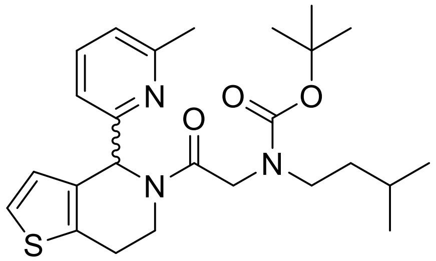

***tert*-Butyl isopentyl(2-(4-(6-methylpyridin-2-yl)-6,7-dihydrothieno[3,2-*c*]pyridin-5(4*H*)-yl)-2-oxoethyl)carbamate (5d).** The Boc-protected intermediate **5d** was prepared **5c** (0.096 mmol, 25 mg) and **2** (0.096 mmol, 22 mg) using **procedure H** to afford the title compound as a colourless oil (0.066 mmol, 28 mg, 60%). R_f = 0.3 (n-hexane/EtOAc 3:7). ^{1}H-NMR (400 MHz, $CDCl_3$) δ (ppm): 7.49 (q, *J* = 8.0 Hz, 2H), 7.22-7.10 (m, 2H), 7.05 (t, *J* = 7.1 Hz, 2H), 6.98 (d, *J* = 7.7 Hz, 1H), 6.65-6.94 (m, 3H), 6.53 (s, 1H), 6.02 (s, 1H), 5.97 (s, 1H), 4.99-4.83 (m, 1H), 4.58 (d, *J* = 17 Hz, 1H), 4.38-4.27 (m, 1H), 4.13-3.98 (m, 2H), 3.44 (dt, *J* = 15 Hz, *J* = 7.8 Hz, 1H), 3.10-3.35 (m, 2H), 3.12-2.76 (m, 5H), 2.55 (s, 3H), 2.45 (s, 2H), 1.59-1.50 (m, 1H), 1.49-1.23 (m, 14H), 1.23-1.11 (m, 1H), 0.93-0.83 (m, 10H). ^{13}C-NMR (101 MHz, $CDCl_3$) δ (ppm): 168.7, 158.9, 158.6, 136.8, 126.6, 126.2, 123.3, 122.3, 121.9, 118.9, 118.4, 79.5, 59.0, 58.7, 56.5, 49.4, 48.7, 46.6, 46.3, 41.1, 37.1, 36.9, 36.5, 29.7, 28.4, 26.1, 25.8, 24.9, 24.5, 22.6, 22.6. HRMS (ESI): calculated for $C_{25}H_{36}N_3O_3S$ $[M+H]^+$: 458.2472, found: 458.2462.

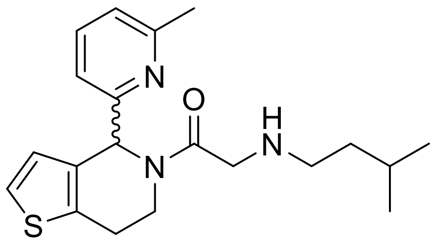

2-(Isopentylamino)-1-(4-(6-methylpyridin-2-yl)-6,7-dihydrothieno[3,2-*c*]pyridin-5(4*H*)-yl)ethan-1-one (5). Compound **5** was prepared from the Boc-protected intermediate **5d**

(0.045 mmol, 19 mg) using **procedure I** to afford the title compound as a colourless oil (0.045 mmol, 15 mg, 100%). R_f = 0.18 (n-hexane/EtOAc 3:1). ^{1}H-NMR (500 MHz, $CDCl_3$) δ (ppm): 7.53-7.45 (m, 1H), 7.14 (d, *J* = 5.2 Hz, 1H), 7.10-7.02 (m, 1H), 6.98 (d, *J* = 7.7 Hz, 1H), 6.90 (d, *J* = 5.2 Hz, 1H), 6.85 (d, *J* = 5.2 Hz, 1H), 6.80 (d, *J* = 7.7 Hz, 1H), 6.56 (d, *J* = 1.5 Hz, 1H), 5.97 (s, 1H), 4.98-4.90 (m, 1H), 4.09-3.94 (m, 2H), 3.70 (d, *J* = 16 Hz, 1H), 3.57 (q, *J* = 16 Hz, 1H), 3.08-2.83 (m, 3H), 2.69-2.57 (m, 3H), 2.54 (s, 2H), 2.45 (s, 1H), 1.60-1.65 (m, 1H), 1.46-1.36 (m, 2H), 1.23-1.12 (m, 1H), 0.92-0.85 (m, 6H). ^{13}C-NMR (125 MHz, $CDCl_3$) δ (ppm): 171.0, 169.8, 159.0, 158.6, 158.4, 136.8, 136.6, 135.6, 134.1, 132.9, 132.2, 126.6, 126.2, 123.3, 122.8, 122.4, 121.8, 118.8, 118.2, 58.8, 56.4, 51.1, 50.9, 50.6, 48.3, 48.1, 40.6, 39.0, 38.8, 36.8, 26.0, 25.6, 24.8, 24.6, 24.5, 22.6, 22.6. HRMS (ESI): calculated for $C_{20}H_{28}N_3OS$ [M+H]$^+$: 358.1948, found: 358.1968.

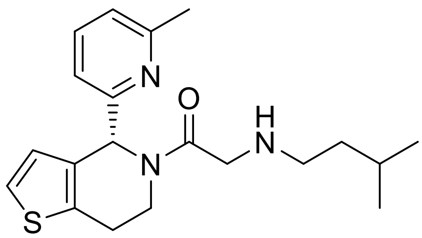

2-(Isopentylamino)-1-(4-(6-methylpyridin-2-yl)-6,7-dihydrothieno[3,2-*c*]pyridin-5(4*H*)-yl)ethan-1-one ((*R*)-5). The (*R*)-enriched derivative **(*R*)-5** was obtained from **(*R*)-2** (10 mg, 0.04 mmol, *er* 87:13) using **procedures H** and **I** to afford the title compound as a colourless oil (0.02 mmol, 7 mg, 50% over 2 steps, *er* 87:13 (Figure S5)). Characterisation data were consistent with **5**.

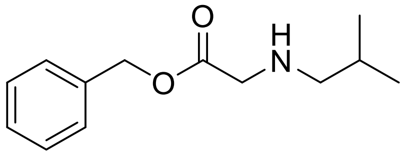

Benzyl [(2-methylpropyl)amino]acetate (6a). Compound **6a** was prepared from benzyl bromoacetate (2.4 mmol, 0.53 mL) and isobutylamine (11 mmol, 1.0 mL) using **procedure E** to afford the title compound as a colourless oil (2.4 mmol, 530 mg, 71%). R_f = 0.29 (n-hexane/EtOAc/NEt_3 70:30:1). ^{1}H-NMR (400 MHz, $CDCl_3$) δ (ppm): 7.42-7.28 (m, 5H), 5.17 (s, 2H), 3.45 (d, *J* = 0.8 Hz, 2H), 2.42 (d, *J* = 6.8 Hz, 2H), 1.73 (m, 1H), 0.91 (d, *J* = 6.8 Hz, 6H). ^{13}C-NMR (101 MHz, $CDCl_3$) δ (ppm): 172.5, 135.7, 128.6, 128.4, 66.5, 57.6, 51.2, 28.5, 20.6. HRMS (ESI): calculated for $C_{13}H_{20}NO_2$ [M+H]$^+$: 222.1494, found: 222.1503.

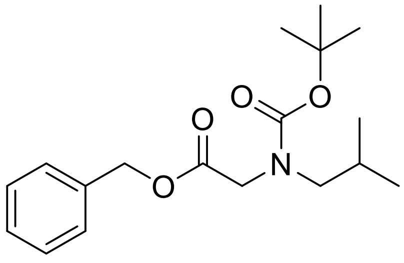

Benzyl [(*N*-*tert*-butoxycarbonyl)(2-methylpropyl)amino]acetate (6b). Compound **6b** was prepared from **6a** (2.3 mmol, 500 mg), TEA (3.5 mmol, 0.48 mL) and di-*tert*-butyl dicarbonate (2.3 mmol, 500 mg) using **procedure F** to afford the title compound as a colorless oil (2.1 mmol, 670 mg, 90%). R_f = 0.28 (n-hexane/EtOAc 1:4). ^{1}H-NMR (400 MHz, $CDCl_3$) δ(ppm): 7.42-7.31 (m, 5H), 5.22 – 5.16 (m, 2H), 4.02 (s, 1H), 3.92 (s, 1H), 3.11 (dd, *J* = 18, 7.4 Hz, 2H), 1.84 (m, 1H), 1.43-1.38 (m, 9H), 0.90 (t, *J* = 6.5 Hz, 6H). ^{13}C-NMR (101 MHz, $CDCl_3$) δ (ppm): 128.6, 128.5, 128.4, 128.3, 128.2, 80.0, 66.7, 66.7, 56.0, 55.8, 50.1, 49.4, 28.3, 28.2, 27.9, 27.6, 20.1, 20.0, 14.2. HRMS (ESI): calculated for $C_{18}H_{28}NO_4$ $[M+H]^+$: 322.2018, found: 322.2026.

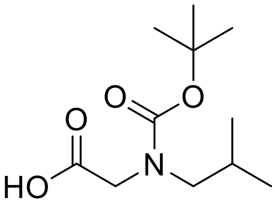

[(*N*-*tert*-Butoxycarbonyl)(2-methylpropyl)amino]acetate (6c). Compound **6c** was obtained from **6b** (2.8 mmol, 640 mg), 10% Pd/C (0.8 mmol, 880 mg) and ammonium formate (16 mmol, 1.0 g) using **procedure G** to afford the title compound as a brown oil (1.2 mmol, 370 mg, 42%). ^{1}H-NMR (400 MHz, $CDCl_3$) δ (ppm): 10.75 (s, 1H), 4.00 (s, 1H), 3.91 (s, 1H), 3.10 (dd, *J* = 13, 7.3 Hz, 2H), 1.84 (m, 1H), 1.46 (d, *J* = 16 Hz, 9H), 0.90 (d, *J* = 6.7 Hz, 6H). ^{13}C-NMR (101 MHz, $CDCl_3$) δ (ppm): 80.9, 80.4, 56.2, 55.7, 49.6, 29.7, 28.3, 28.2, 27.8, 27.6, 20.0. HRMS (ESI): calculated for $C_{11}H_{20}NO_4$ $[M+H]^+$: 230.1392, found: 230.1399.

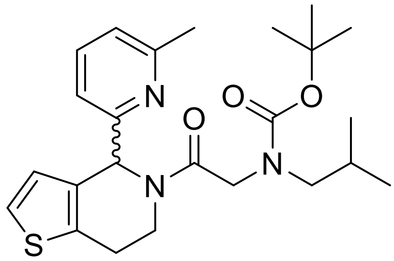

***tert*-Butyl isobutyl(2-(4-(6-methylpyridin-2-yl)-6,7-dihydrothieno[3,2-*c*]pyridin-5(4*H*)-yl)-2-oxoethyl)carbamate (6d).** The Boc-protected intermediate **6d** was prepared from **6c** (0.090 mmol, 20 mg) and **2** (0.090 mmol, 20 mg) using **procedure H** to afford the title compound as a colourless oil (0.080 mmol, 37 mg, 89%). R_f = 0.23 (n-hexane/EtOAc 3:7). ^{1}H-NMR (400 MHz, $CDCl_3$) δ (ppm): 7.49 (q, *J* = 7.7 Hz, 1H), 7.22-6.94 (m, 2H), 6.75-6.92 (m, 2H), 6.53 (s, 1H), 5.98 (d, *J* = 17 Hz, 1H), 4.98-4.83 (m, 1H), 4.62 (q, *J* = 17 Hz, 1H), 4.47-

4.26 (m, 1H), 4.17-3.89 (m, 1H), 3.34-2.76 (m, 5H), 2.57-2.39 (m, 3H), 1.94-1.79 (m, 2H), 1.51-1.25 (m, 9H), 0.94-0.81 (m, 6H). ^{13}C-NMR (101 MHz, $CDCl_3$) δ (ppm): 158.9, 158.6, 156.5, 136.8, 135.9, 126.1, 123.3, 122.4, 118.6, 79.5, 59.0, 58.6, 56.5, 55.6, 50.1, 49.4, 36.8, 36.4, 29.7, 28.4, 28.3, 27.6, 27.4, 25.7, 24.9, 24.5, 20.1, 14.2. HRMS (ESI): calculated for: $C_{24}H_{34}N_3O_3S$ [M+H]$^+$: 444.2315, found: 444.2323.

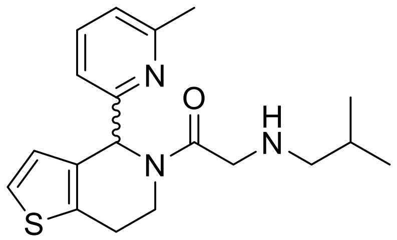

2-(Isobutylamino)-1-(4-(6-methylpyridin-2-yl)-6,7-dihydrothieno[3,2-*c*]pyridin-5(4*H*)-yl)ethan-1-one (6). Compound **6** was prepared from Boc-protected intermediate **6d** (0.080 mmol, 37 mg) using **procedure I** to afford the title compound as a colourless oil (0.080 mmol, 28 mg, 100%). Chiral HPLC (Method A): room temperature = 9.6 min (50%), 13.8 min (50%). ^{1}H-NMR (500 MHz, $CDCl_3$) δ (ppm): 7.50 (q, *J* = 7.6 Hz, 1H), 7.15 (t, J = 5.7 Hz, 1H), 7.06 (dd, *J* = 12 Hz, *J* = 6.4 Hz, 1H), 6.98 (d, *J* = 7.7 Hz, 1H), 6.89 (d, *J* = 5.2 Hz, 1H), 6.85 (d, *J* = 5.2 Hz, 1H), 6.80 (d, *J* = 7.7 Hz, 1H), 6.56 (d, *J* = 1.5 Hz, 1H), 5.97 (s, 1H), 4.98-4.87 (m, 1H), 4.12 (d, *J* = 16 Hz, 1H), 4.06-3.96 (m, 1H), 3.76 (d, *J* = 16 Hz, 1H), 3.59 (q, *J* = 16 Hz, 1H), 3.07-2.81 (m, 1H), 2.54 (s, 2H), 2.50-2.39 (m, 3H), 1.86-1.73 (m, 1H), 0.98-0.90 (m, 6H). ^{13}C-NMR (125 MHz, $CDCl_3$) δ (ppm): 170.6, 169.7, 158.9, 158.7, 158.5, 158.4, 136.9, 136.6, 135.6, 134.1, 132.9, 132.1, 126.6, 126.2, 123.4, 122.8, 122.4, 121.9, 118.8, 118.3, 58.7, 58.1, 57.9, 56.5, 51.2, 50.9, 40.7, 36.8, 28.5, 28.2, 25.6, 24.8, 24.6, 24.5, 20.6, 20.6, 20.5. HRMS (ESI): calculated for $C_{19}H_{26}N_3OS$ [M+H]$^+$: 344.1791, found: 344.1796.

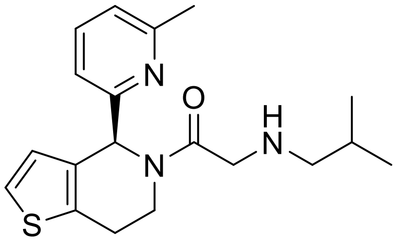

(*S*)-(-)-2-(Isobutylamino)-1-(4-(6-methylpyridin-2-yl)-6,7-dihydrothieno[3,2-*c*]pyridin-5(4*H*)-yl)ethan-1-one ((-)-6). Compound **(-)-6** was obtained from compound **6** by preparative chiral HPLC (**Method B**) (*er* 100:0). Chiral HPLC (**Method A**): 13.5 min (100%). $[\alpha]^{23}_D$ (c = 0.1, $CHCl_3$): -57.2. ^{1}H-NMR (500 MHz, $CDCl_3$) δ (ppm): 7.50 (q, *J* = 7.6 Hz, 1H), 7.32 (d, *J* = 5.7 Hz, 1H), 7.10-7.02 (m, 1H), 6.98 (d, *J* = 7.7 Hz, 1H), 6.90 (d, *J* = 5.2 Hz, 1H), 6.85 (d, *J* = 5.2 Hz, 1H), 6.81 (d, *J* = 7.7 Hz, 1H), 6.56 (s, 1H), 5.98 (s, 1H), 4.99-4.91 (m, 1H), 4.08-3.96 (m, 2H), 3.70 (d, *J* = 16 Hz, 1H), 3.56 (q, *J* = 16 Hz, 1H), 3.09-2.83 (m, 3H), 2.54 (s, 2H), 2.50-2.37 (m, 3H), 1.82-1.71 (m, 1H), 0.95-0.81 (m, 6H). ^{13}C-NMR (125 MHz, $CDCl_3$) δ (ppm): 171.1, 170.0, 159.0, 158.7, 158.4, 136.8, 136.6, 135.6, 134.1, 132.9, 132.3, 126.6, 126.3, 123.4, 122.8, 122.4, 121.9, 118.8, 118.2, 58.8, 58.2, 58.0, 56.4, 51.4, 51.1, 40.6, 36.8, 29.7, 28.6, 28.4, 25.6,

24.8, 24.6, 24.5, 20.7, 20.6, 20.6. HRMS (ESI): calculated for $C_{19}H_{26}N_3OS$ $[M+H]^+$: 344.1791, found: 344.1800.

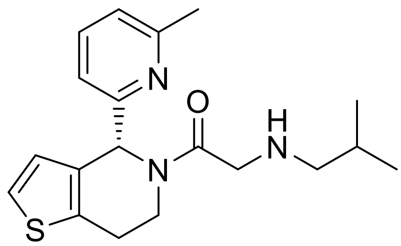

(*R*)-(+)-2-(Isobutylamino)-1-(4-(6-methylpyridin-2-yl)-6,7-dihydrothieno[3,2-*c*]pyridin-5(4*H*)-yl)ethan-1-one ((+)-6, IMP-1575). Compound **(+)-6** was obtained from compound **6** by preparative chiral HPLC (**Method B**) (*er* 100:0). Chiral HPLC (**Method A**): 9.4 min (100%). $[\alpha]^{23}_{D}$ (c = 0.67, $CHCl_3$): +43.6. ^{1}H-NMR (500 MHz, $CDCl_3$) δ (ppm): 7.50 (q, *J* = 7.6 Hz, 1H), 7.17-7.12 (m, 1H), 7.10-7.02 (m, 1H), 6.98 (d, *J* = 7.7 Hz, 1H), 6.90 (d, *J* = 5.2 Hz, 1H), 6.85 (d, *J* = 5.2 Hz, 1H), 6.80 (d, *J* = 7.7 Hz, 1H), 6.56 (d, *J* = 1.5 Hz, 1H), 5.97 (s, 1H), 5.00-4.90 (m, 1H), 4.11-3.97 (m, 1.7H), 3.73 (d, *J* = 16.2 Hz, 1H), 3.57 (q, *J* = 16 Hz, 1H), 3.08-2.83 (m, 3H), 2.54 (s, 2H), 2.52-2.38 (m, 3H), 1.84-1.71 (m, 1H), 0.96-0.84 (m, 6H). ^{13}C-NMR (125 MHz, $CDCl_3$) δ (ppm): 170.9, 169.9, 159.0, 158.7, 158.6, 158.4, 136.9, 136.6, 135.6, 134.1, 132.9, 132.2, 126.6, 126.2, 123.4, 122.8, 122.4, 121.9, 118.8, 118.2, 58.8, 58.2, 57.9, 56.5, 51.3, 51.0, 40.6, 36.8, 29.7, 28.6, 28.3, 25.6, 24.9, 24.6, 24.5, 20.7, 20.6, 20.6. HRMS (ESI): calculated for $C_{19}H_{26}N_3OS$ $[M+H]^+$: 344.1791, found: 344.1811.

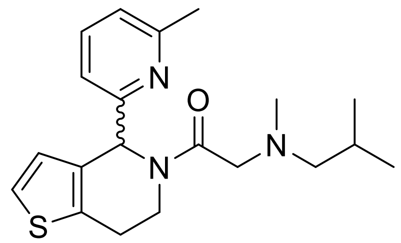

2-(Isobutyl(methyl)amino)-1-(4-(6-methylpyridin-2-yl)-6,7-dihydrothieno[3,2-*c*]pyridin-5(4*H*)-yl)ethan-1-one (7). The required tertiary amine was prepared from **2** via the chloroacetamide (66 mg crude yield, 0.22 mmol) and *N*-methyl isobutylamine (29 µL, 0.24 mmol) using **procedure K** to afford the title compound as a yellow oil (0.022 mmol, 7.9 mg, 10%). R_f = 0.25 (n-hexane/EtOAc 1:1.5 with 1% TEA) ^{1}H-NMR (400 MHz, $CDCl_3$) δ (ppm): 7.52-7.41 (m, 1H), 7.16-7.08 (m, 1H), 7.06 (d, *J* = 5.3 Hz, 1H), 7.01 (d, *J* = 7.7 Hz, 1H), 6.97 (d, *J* = 7.7 Hz, 1H), 6.89 (t, *J* = 5.0 Hz, 1H), 6.85 (s, 1H), 6.79 (d, *J* = 7.7 Hz, 1H), 6.55 (d, *J* = 1.6 Hz, 1H), 4.96-4.85 (m, 1H), 4.54 (ddd, *J* = 13.2, 5.4, 1.7 Hz, 1H), 3.96 (d, *J* = 13.2 Hz, 1H), 3.90 (ddd, *J* = 13.3, 11.8, 3.9 Hz, 1H), 3.33-3.17 (m, 1H), 3.15 (d, *J* = 13.3 Hz, 1H), 3.10-2.76 (m, 3H), 2.52 (s, 2H), 2.45 (s, 1H), 2.23 (s, 2H), 2.20 (s, 1H), 2.17-2.13 (m, 1H), 2.11 (dd, *J* = 7.4, 1.4 Hz, 1H), 1.79-1.63 (m, 1H), 0.85 (t, *J* = 6.9 Hz, 3H), 0.82 (d, *J* = 6.6 Hz, 2H), 0.72 (d, *J* = 6.5 Hz, 2H). ^{13}C-NMR (101 MHz, $CDCl_3$) δ (ppm): 170.4, 169.3, 159.3, 159.1, 158.3, 158.1, 136.4, 136.3, 134.8, 134.0, 133.3, 133.1, 126.6, 126.3,

122.7, 122.3, 121.7, 121.5, 118.5, 117.9, 66.2, 65.9, 63.2, 62.6, 58.4, 56.0, 42.4, 41.83, 41.4, 36.2, 25.8, 24.8, 24.4, 24.3, 20.7, 20.6, 20.4, 20.2. HRMS (ESI): calculated for $C_{20}H_{28}N_3OS$ $[M+H]^+$: 358.1953, found: 358.1962.

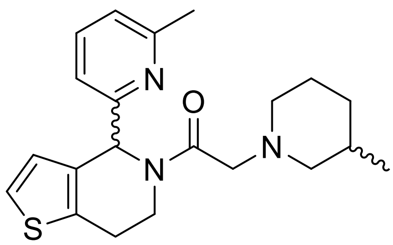

2-(3-Methylpiperidin-1-yl)-1-(4-(6-methylpyridin-2-yl)-6,7-dihydrothieno[3,2-*c*]pyridin-5(4*H*)-yl)ethan-1-one (8). The required tertiary amine was prepared from **2** via the chloroacetamide (66 mg crude yield, 0.22 mmol) and 3-methyl piperidine (28 µL, 0.24 mmol) using **procedure K** to afford the title compound as a yellow oil (0.024 mmol, 9.0 mg, 11%). R_f = 0.32 (n-hexane/EtOAc 1:1.5 with 1% TEA). ^1H NMR (400 MHz, $CDCl_3$) δ (ppm): 7.51-7.43 (m, 2H), 7.12 (d, J = 5.5 Hz, 2H), 7.06 (dd, J = 5.4, 2.1 Hz, 1H), 7.03-6.94 (m, 4H), 6.92 (dd, J = 5.2, 1.1 Hz, 1H), 6.83 (dd, J = 7.7, 2.2 Hz, 1H), 6.58 (s, 1H), 6.54-6.50 (m, 2H), 4.92 (dt, J = 13, 3.4 Hz, 1H), 4.49-4.37 (m, 1H), 3.94-3.80 (m, 1H), 3.65 (dd, J = 14, 11 Hz, 1H), 3.32 (dd, J = 16, 14 Hz, 2H), 3.18 (d, J = 4.2 Hz, 1H), 3.15 (d, J = 4.2 Hz, 1H), 3.10-2.96 (m, 1H), 2.96-2.86 (m, 2H), 2.86-2.67 (m, 5H), 2.62-2.59 (m, 1H), 2.52 (s, 3H), 2.46 (s, 1H), 2.45 (s, 2H), 2.15 (d, J = 7.5 Hz, 1H), 1.98 (d, J = 18 Hz, 3H), 1.88 (s, 1H), 1.78-1.20 (m, 6H),0.87-0.79 (m, 4H), 0.77 (dd, J = 6.7, 2.2 Hz, 3H). ^{13}C NMR (101 MHz, $CDCl_3$) δ (ppm): 170.2, 169.4, 159.4, 158.6, 158.4, 136.6, 135.2, 134.3, 133.4, 127.0, 126.7, 123.0, 122.6, 121.9, 121.8, 118.7, 118.0, 63.1, 62.4, 62.3, 62.1, 62.0, 61.7, 59.2, 56.4, 54.3, 54.2, 53.8, 42.0, 41.3, 37.00, 32.6, 31.9, 31.4, 31.2, 30.9, 30.6, 26.14, 25.7, 25.5, 25.4, 25.2, 25.1, 24.7, 19.7, 19.5. HRMS (ESI): calculated for $C_{21}H_{28}N_3OS$ $[M+H]^+$: 370.1953, found: 370.1949.

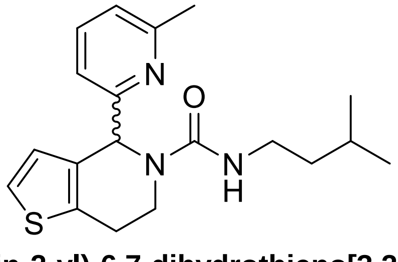

***N*-Isopentyl-4-(6-methylpyridin-2-yl)-6,7-dihydrothieno[3,2-*c*]pyridine-5(4*H*)-carboxamide (9).** Compound **9** was prepared from isopentylamine (0.076 mmol, 7 mg) and **2** (0.096 mmol, 22 mg) using **procedure L** to afford the title compound as a colourless oil (0.048 mmol, 13 mg, 64%). R_f = 0.2 (n-hexane/EtOAc 3:7). ^1H-NMR (400 MHz, $CDCl_3$) δ (ppm): 7.52 (t, J = 7.7 Hz, 1H), 7.21 (t, J = 5.2 Hz, 1H), 7.13-7.06 (m, 2H), 6.77 (d, J = 7.7 Hz, 1H), 6.61 (d, J = 5.2 Hz, 1H), 5.88 (s, 1H), 4.64-4.55 (m, 1H), 3.33-3.20 (m, 2H), 3.06-2.79 (m, 3H), 2.56 (s, 3H), 1.71 (q, J = 6.7 Hz, 1H), 1.56-1.39 (m, 2H), 0.94 (d, J = 6.7 Hz, 6H). ^{13}C-NMR (101 MHz, $CDCl_3$) δ (ppm): 159.9, 158.9, 157.6, 137.4, 136.7, 132.1, 125.8, 123.0, 122.6,

119.0, 59.4, 39.1, 39.1, 37.1, 25.8, 25.3, 24.2, 22.6, 22.6. HRMS (ESI): calculated for $C_{19}H_{26}N_3OS$ $[M+H]^+$: 344.1791, found: 344.1781.

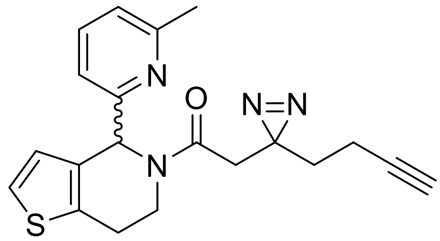

2-(3-(But-3-yn-1-yl)-3*H*-diazirin-3-yl)-1-(4-(6-methylpyridin-2-yl)-6,7-dihydrothieno[3,2-*c*]pyridin-5(4*H*)-yl)ethanone (10). 2-(3-(But-3-yn-1-yl)-3H-diazirin-3-yl)acetic acid was synthesised according to known literature procedures.[4-5] Probe **10** was prepared from **2** (18 mg, 0.078 mmol, 1.0 eq.) and 2-(3-(but-3-yn-1-yl)-3H-diazirin-3-yl)acetic acid (13 mg, 0.078 mmol, 1.0 eq.) using **procedure H** to afford a crude product that was purified by preparative TLC to afford the title compound as a white oil (0.030 mmol, 11 mg, 22%). R_f = 0.85 (EtOAc). ^{1}H NMR (400 MHz, $CDCl_3$) δ(ppm): 7.52 (t, *J* = 7.7 Hz, 1H,), 7.47 (t, *J* = 7.7 Hz, 1H), 7.16 (d, *J* = 8.0 Hz, 1H), 7.15 (d, *J* = 5.2 Hz, 1H), 7.09 (d, 5.2 Hz, 1H), 7.05 (d, *J* = 8.0 Hz, 1H), 6.99 (d, *J* = 7.6 Hz, 1H), 6.83 (d, *J* = 5.2 Hz, 1H), 6.73 (d, *J* = 7.6 Hz, 1H), 6.56 (s, 1H), 5.84 (s, 1H), 4.92 (d, *J* = 12.5 Hz, 1H), 4.89 (m, 1H), 4.10 (td, *J* = 12.5, 4.3 Hz, 1H), 3.80 (dd, *J* = 13.8, 4.3 Hz, 1H), 3.48 (s, 2H), 3.21 (d, *J* = 16.6 Hz, 1H), 3.05-2.82 (m, 4H), 2.64 (d, *J* = 16.6 Hz, 1H), 2.52 (s, 3H), 2.45 (s, 3H), 2.07 (td, *J* = 7.0, 2.7 Hz, 2H), 2.03 (td, *J* = 7.0, 2.7 Hz, 2H), 1.94 (t, *J* = 2.6 Hz, 1H), 1.92 (t, *J* = 2.6 Hz, 1H), 1.84 (t, *J* = 7.0 Hz, 2H), 1.77 (t, *J* = 7.0 Hz, 2H). ^{13}C-NMR (101 MHz, $CDCl_3$) δ (ppm): 168.6, 167.2, 158.9, 158.9, 158.6, 158.6, 137.0, 136.8, 136.0, 134.2, 133.0, 132.3, 126.7, 126.2, 123.7, 123.1, 122.7, 122.1, 119.1, 118.5, 83.1, 69.4, 69.2, 60.6, 59.8, 43.5, 42.5, 39.7, 39.5, 36.4, 35.7, 32.6, 31.9, 26.6, 26.0, 24.8, 24.6. HRMS (ESI): calculated for $C_{20}H_{20}N_4OS$ $[M+H]^+$: 365.1431, found: 365.1439.

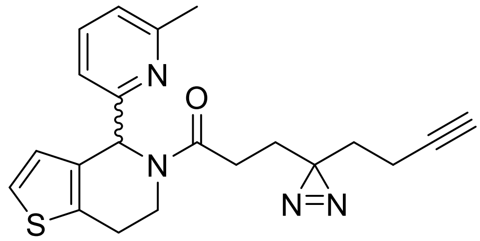

3-(3-(But-3-yn-1-yl)-3*H*-diazirin-3-yl)-1-(4-(6-methylpyridin-2-yl)-6,7-dihydrothieno[3,2-*c*]pyridin-5(4*H*)-yl)propan-1-one (11). 3-(3-(But-3-yn-1-yl)-3H-diazirin-3-yl)propanoic acid was synthesised according to known literature procedures.[6] Probe **11** was prepared from **2** (18 mg, 0.078 mmol, 1.0 eq.) and 3-(3-(but-3-yn-1-yl)-3H-diazirin-3-yl)propanoic acid (13 mg, 0.078 mmol, 1.0 eq.) using **procedure H** to afford a crude product that was purified by preparative HPLC-MS to afford the title compound as a white solid (0.021 mmol, 8 mg, 27 %) R_f = 0.87 (EtOAc/n-hexane 20:1). ^{1}H NMR (500 MHz, $CDCl_3$) δ(ppm): 7.50 (t, *J* = 8.0 Hz, 1H), 7.49 (t, *J* = 8.0 Hz, 1H), 7.14 (d, *J* = 5.2 Hz, 1H), 7.13 (d, *J* = 8.0 Hz, 1H), 7.08 (d, *J* = 5.2 Hz, 1H), 7.06 (d, *J* = 8.0 Hz, 1H), 6.97 (d, *J* = 7.5 Hz, 1H), 6.91 (d, *J* = 5.2 Hz, 1H), 6.85 (d,

J = 5.2 Hz, 1H), 6.80 (d, *J* = 7.5 Hz, 1H), 6.55 (s, 1H), 6.01 (s, 1H), 4.97-4.90 (m, 1H), 4.06-3.99 (m, 1H), 3.02-2.90 (m, 2H), 2.89-2.81 (m, 1H), 2.76 (dt, *J* = 16, 7.7 Hz, 1H), 2.57 (s, 3H), 2.45 (s, 3H), 2.36 (dt, *J* = 16, 7.7 Hz, 1H), 2.28-2.11 (m, 2H), 2.02 (td, *J* = 7.6, 2.6 Hz, 2H), 1.96 (t, *J* = 2.6 Hz, 1H), 1.94 (t, *J* = 2.6 Hz, 1H), 1.89 (t, *J* = 7.7 Hz, 2H), 1.84 (t, *J* = 7.7 Hz,, 2H), 1.66 (t, *J* = 7.5 Hz, 2H), 1.66 (t, *J* = 7.5 Hz, 2H). ^{13}C-NMR (126 MHz, $CDCl_3$) δ (ppm): 171.5, 170.0, 159.2, 159.0, 158.8, 158.5, 137.0, 136.8, 135.8, 134.4, 132.8, 132.7, 127.5, 126.5, 123.5, 123.0, 122.5, 122.0, 119.0, 118.3, 83.0, 82.9, 77.7, 69.3, 69.2, 59.7, 56.4, 41.6, 36.8, 32.7, 32.6, 32.7, 28.3, 28.1, 28.0, 27.7, 25.8, 25.0, 24.8, 24.7, 13.5, 13.5. HRMS (ESI): calculated for $C_{21}H_{22}N_4OS$ [M+H]$^+$: 379.1587, found: 379.1591.

NMR

1.1. 1-(4-(6-Methylpyridin-2-yl)-6,7-dihydrothieno[3,2-*c*]pyridin-5(4*H*)-yl)ethan-1-one (3)

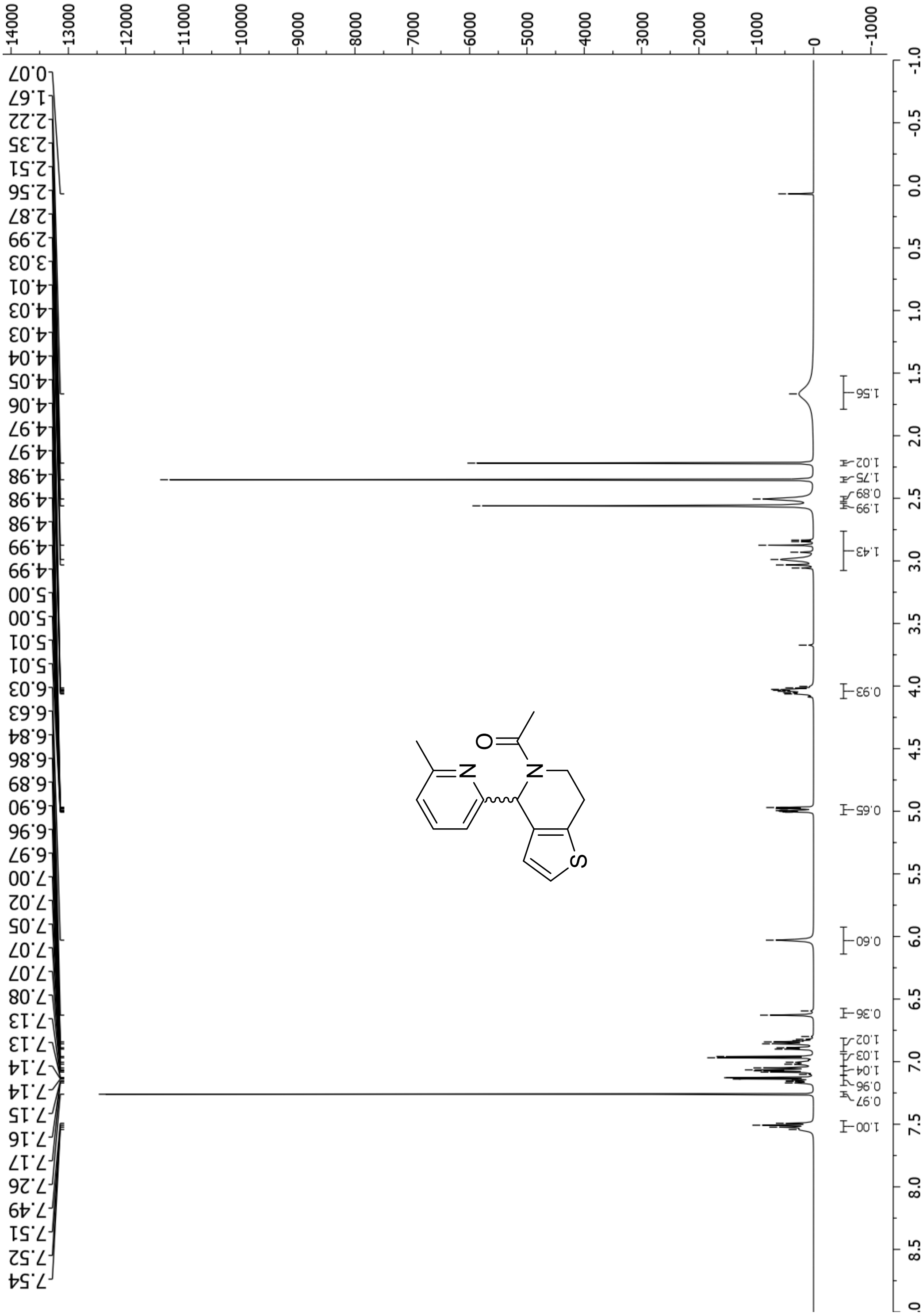

Figure S11. ^{1}H NMR (500 MHz, $CDCl_3$, 298 K) of **3**.

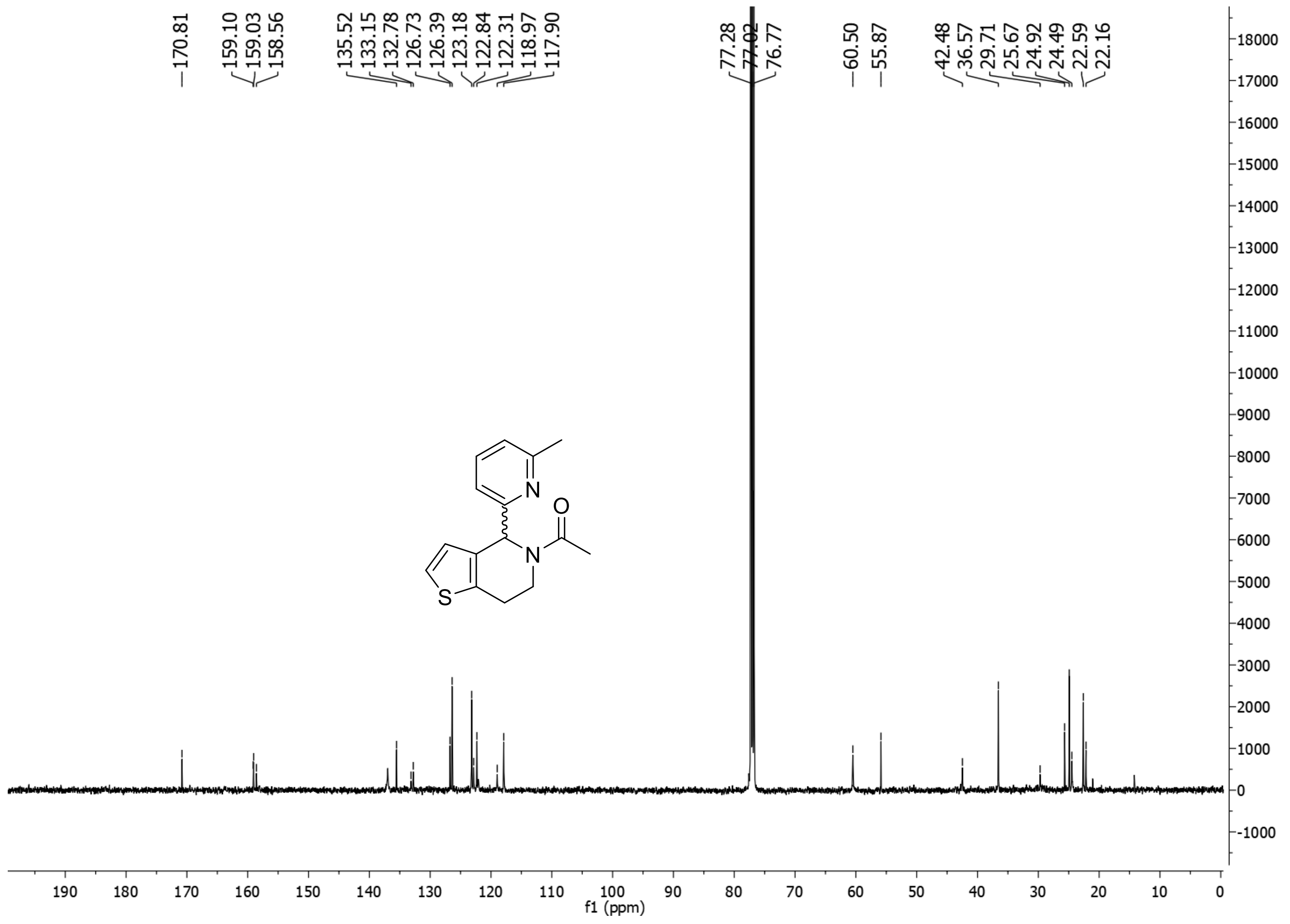

Figure S12. ${}^{13}C$ NMR (500 MHz, $CDCl_3$, 298 K) of **3**.

1.2. 2-(S)-(2-Methylbutylamino)-1-[4-(6-methylpyridin-2-yl)-6,7-dihydro-*4H*-thieno[3,2-*c*]pyridin-5-yl]ethanone (4)

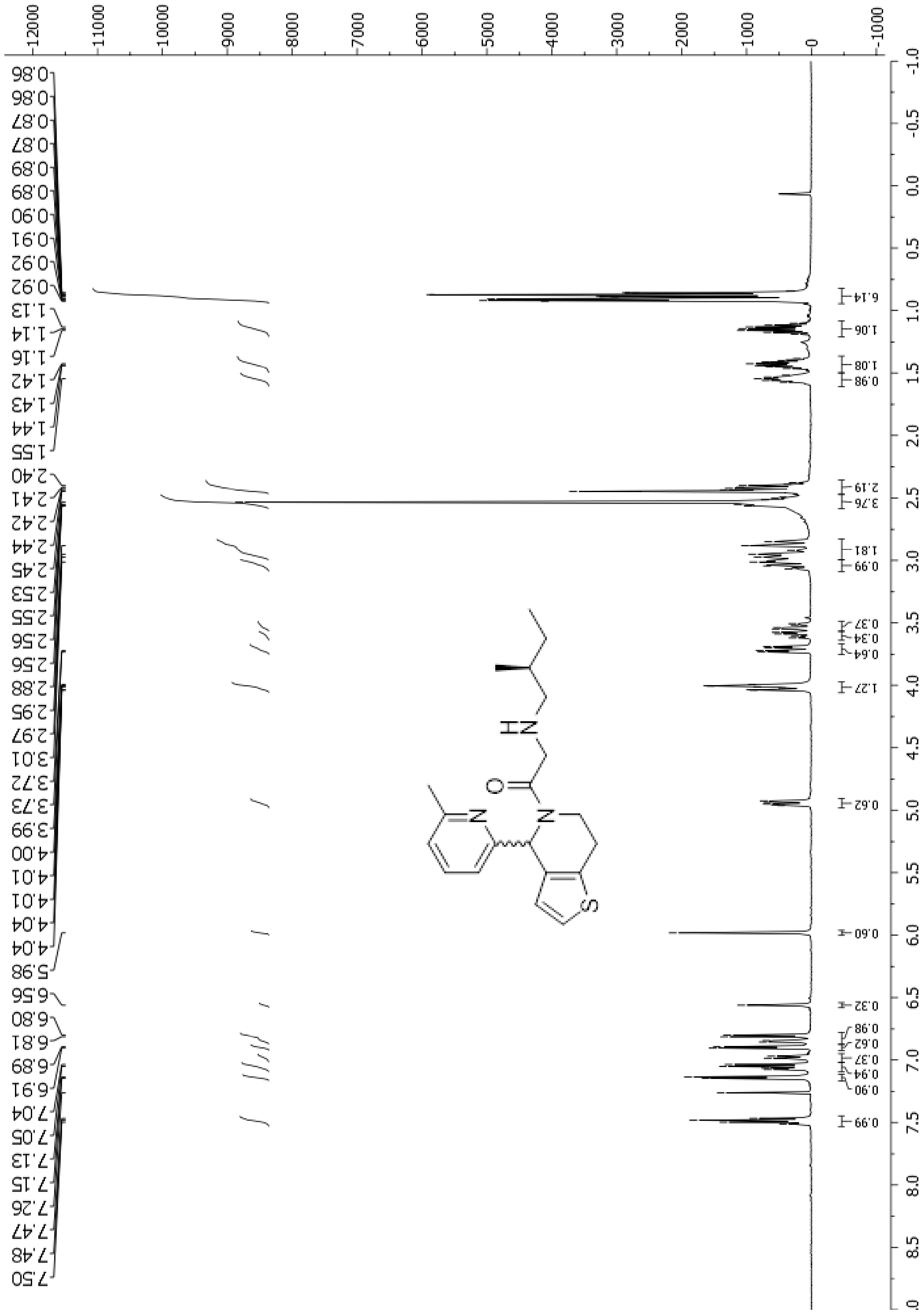

Figure S13. ^{1}H NMR (500 MHz, $CDCl_3$, 298 K) of **4**.

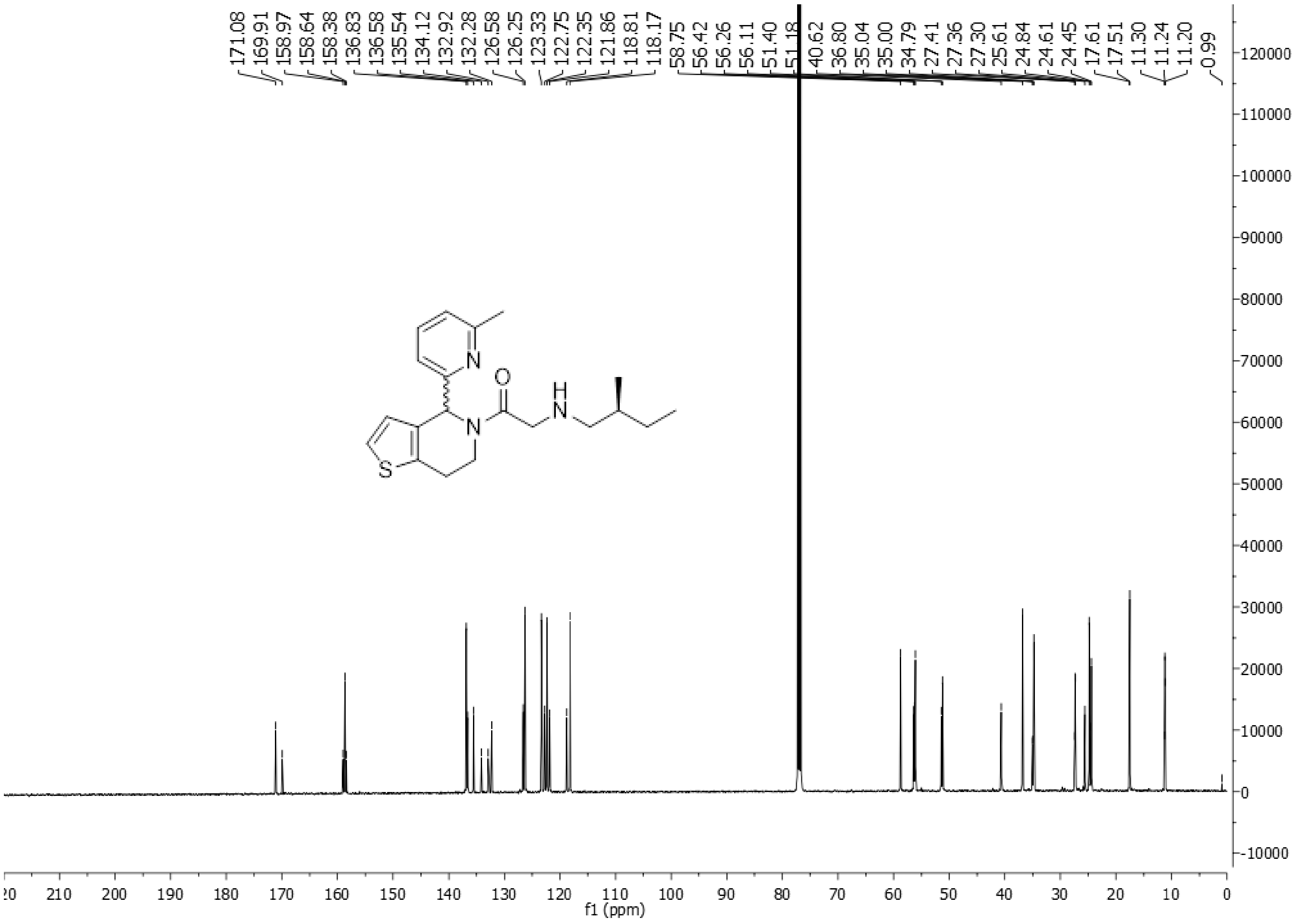

Figure S14. ^{13}C NMR (500 MHz, $CDCl_3$, 298 K) of **4**.

1.3. 2-(Isopentylamino)-1-(4-(6-methylpyridin-2-yl)-6,7-dihydrothieno[3,2-*c*]pyridin-5(4*H*)-yl)ethan-1-one (5)

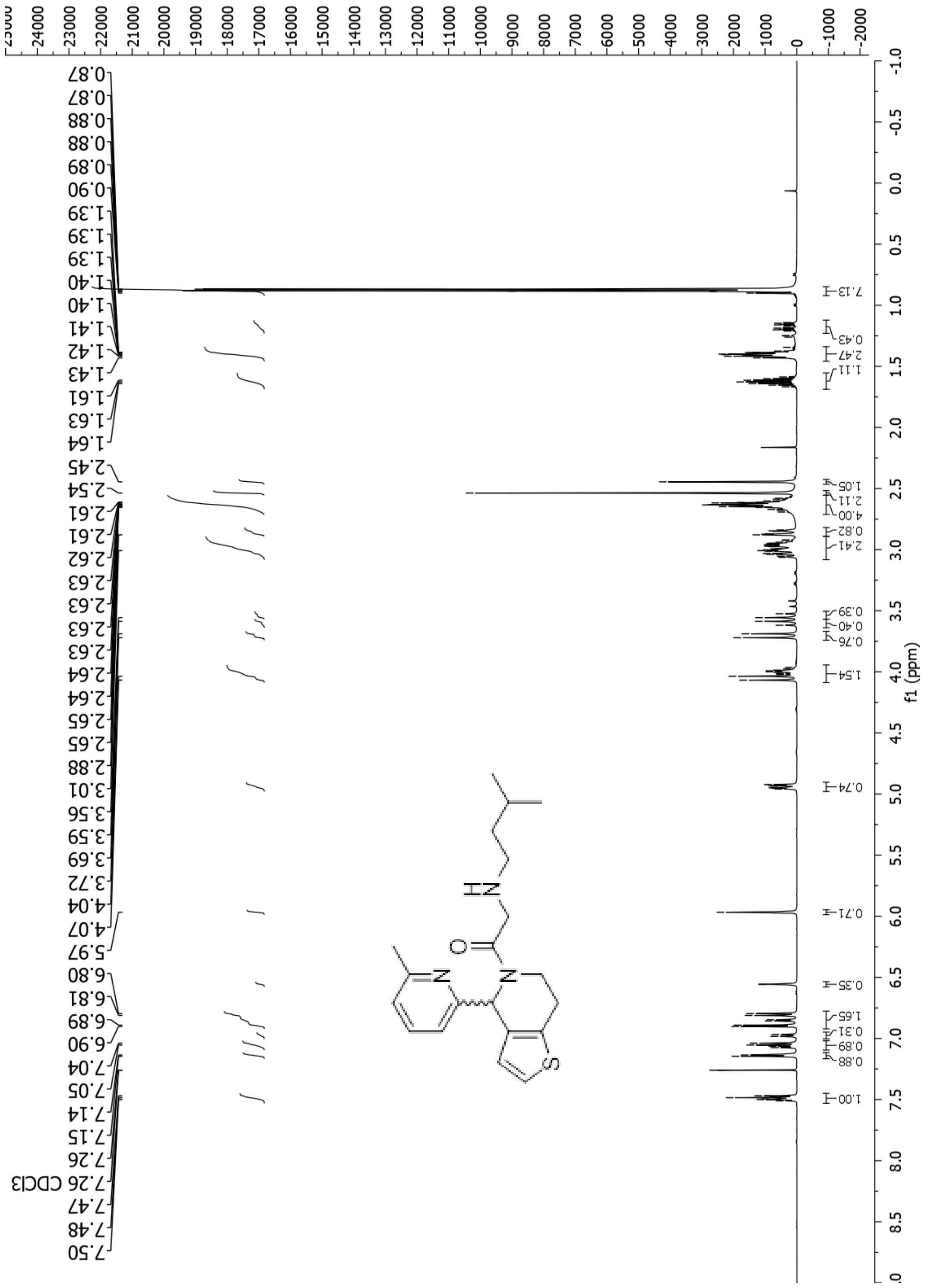

Figure S15. ^{1}H NMR (500 MHz, $CDCl_3$, 298 K) of **5**.

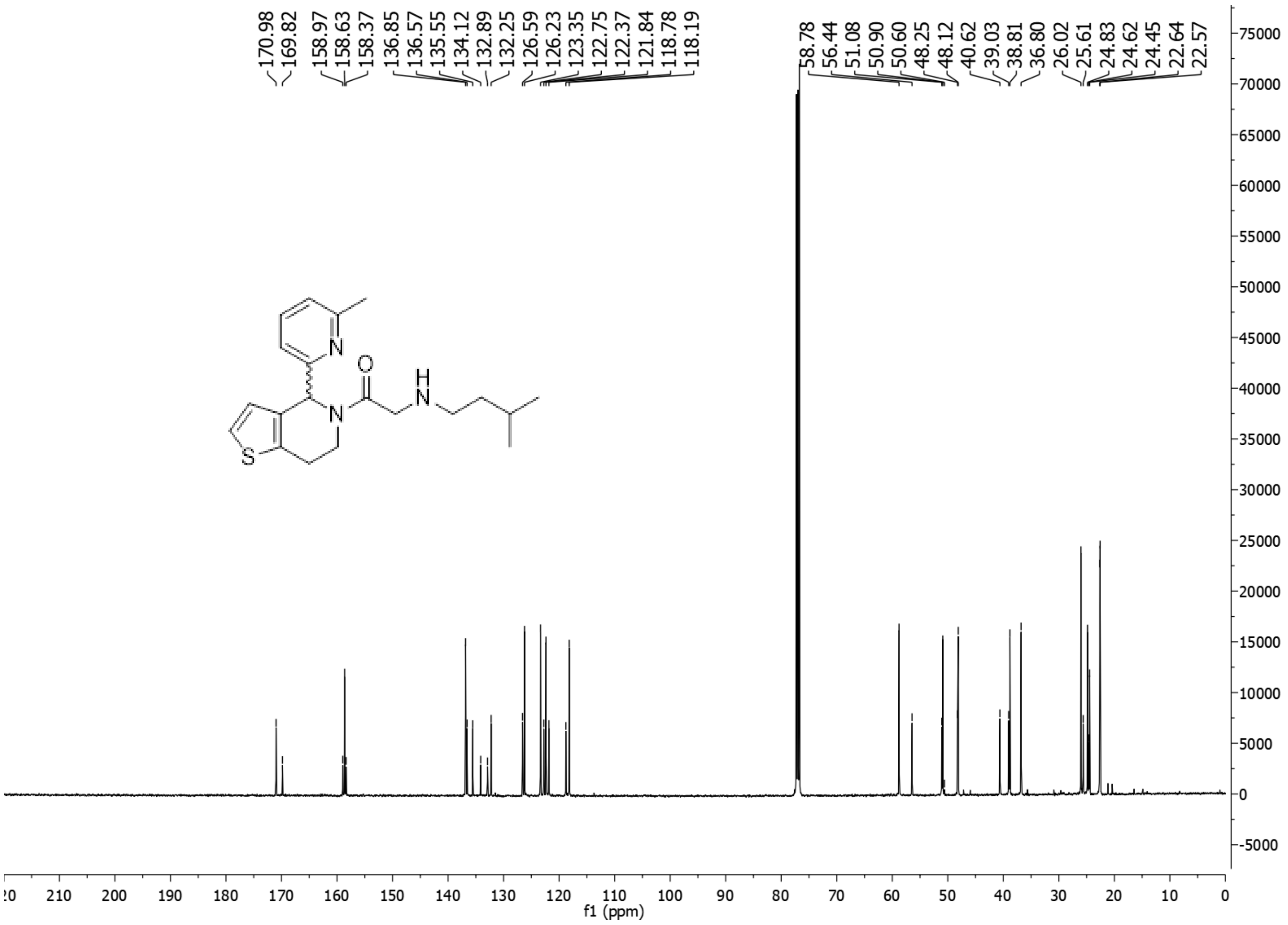

Figure S16. ^{13}C NMR (500 MHz, $CDCl_3$, 298 K) of **5**.

1.4. 2-(2-Methylpropylamino)-1-[4-(6-methylpyridin-2-yl)-6,7-dihydro-4*H*-thieno[3,2-*c*]pyridin-5-yl]ethanone (6)

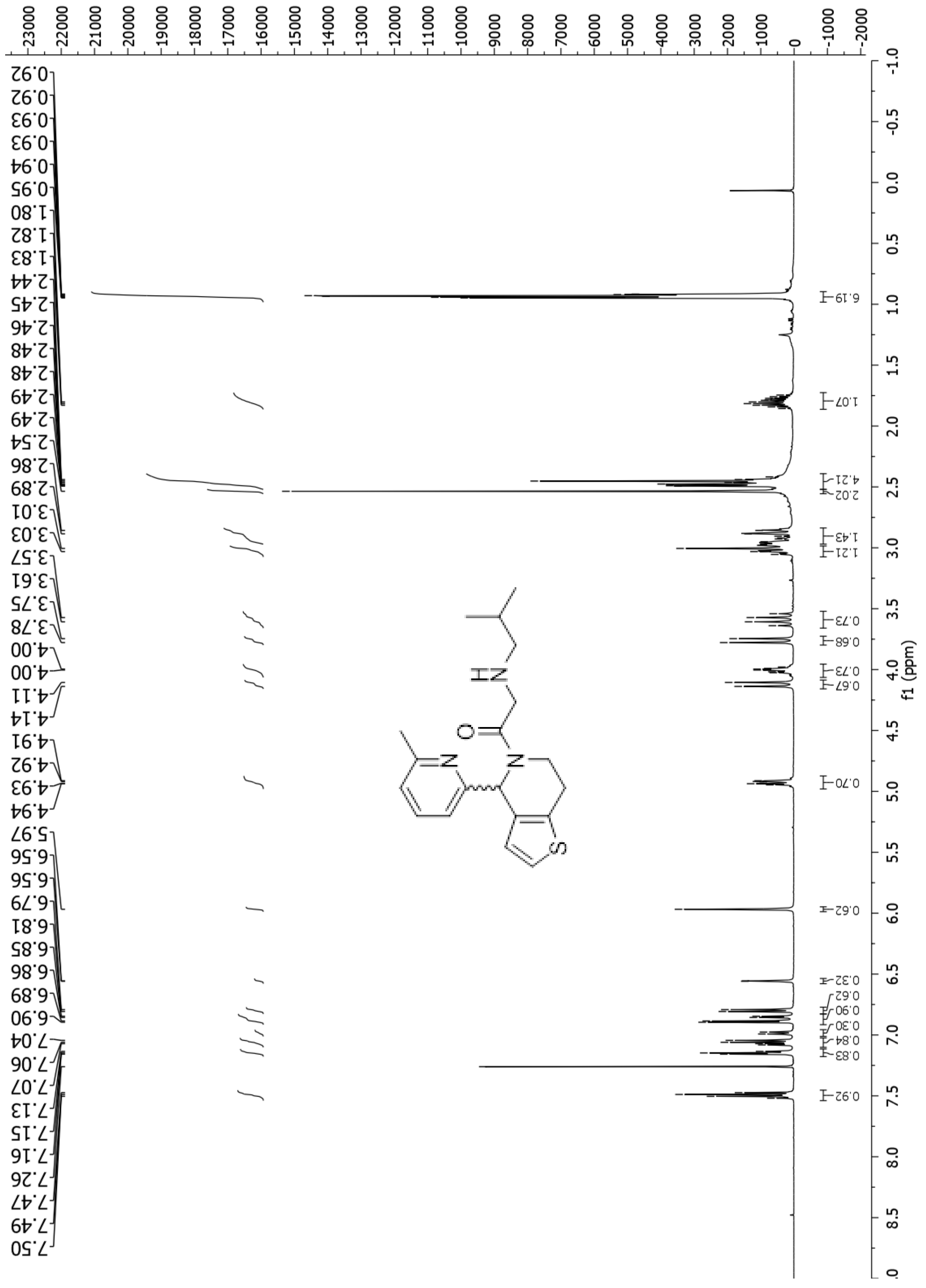

Figure S17. ^{1}H NMR (500 MHz, $CDCl_3$, 298 K) of **6**.

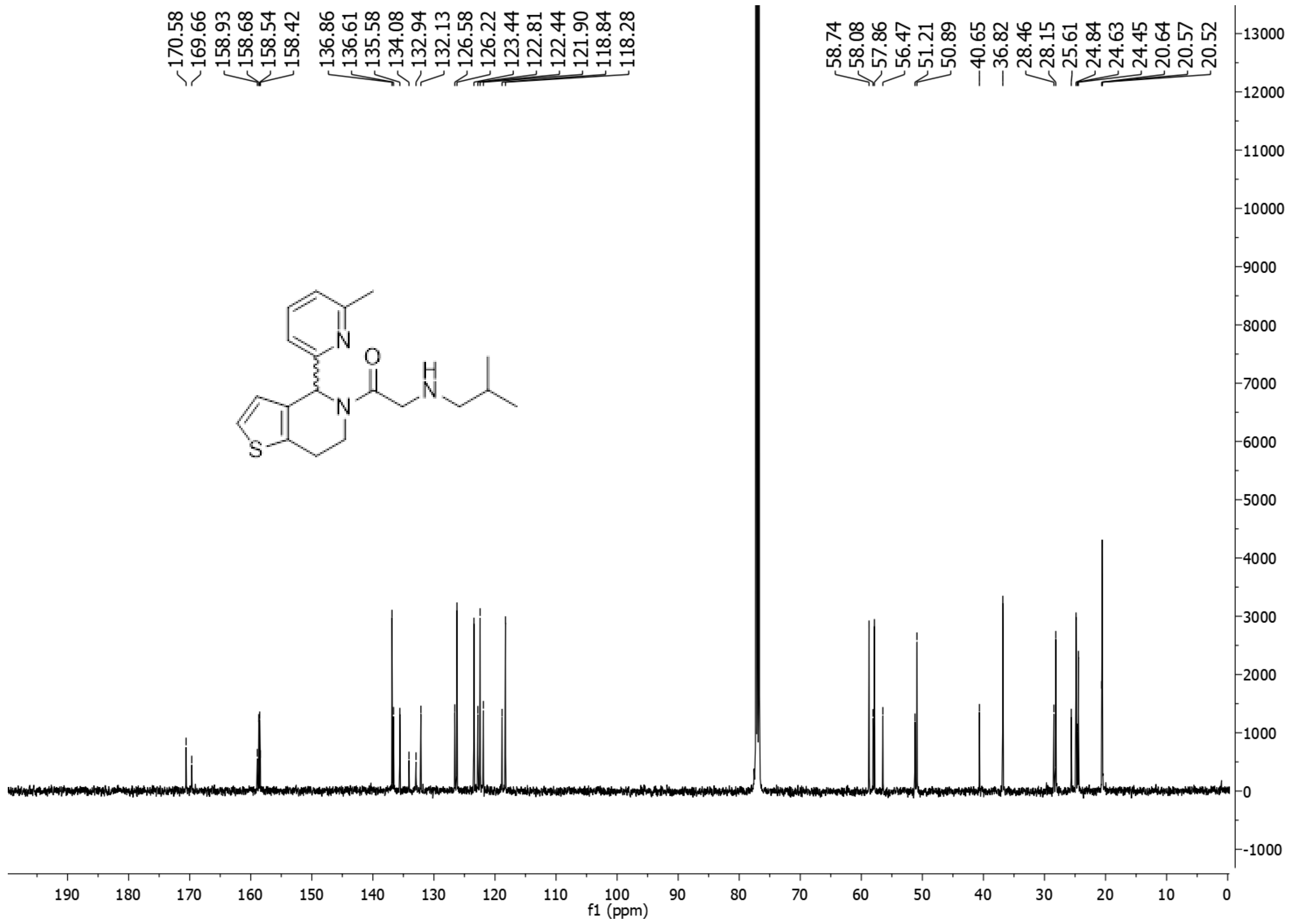

Figure S18. ^{13}C NMR (500 MHz, $CDCl_3$, 298 K) of **6**.

1.5. (*S*)-(-)-2-(2-Methylpropylamino)-1-[4-(6-methylpyridin-2-yl)-6,7-dihydro-4*H*-thieno[3,2-*c*]pyridin-5-yl]ethenone [(-)-6]

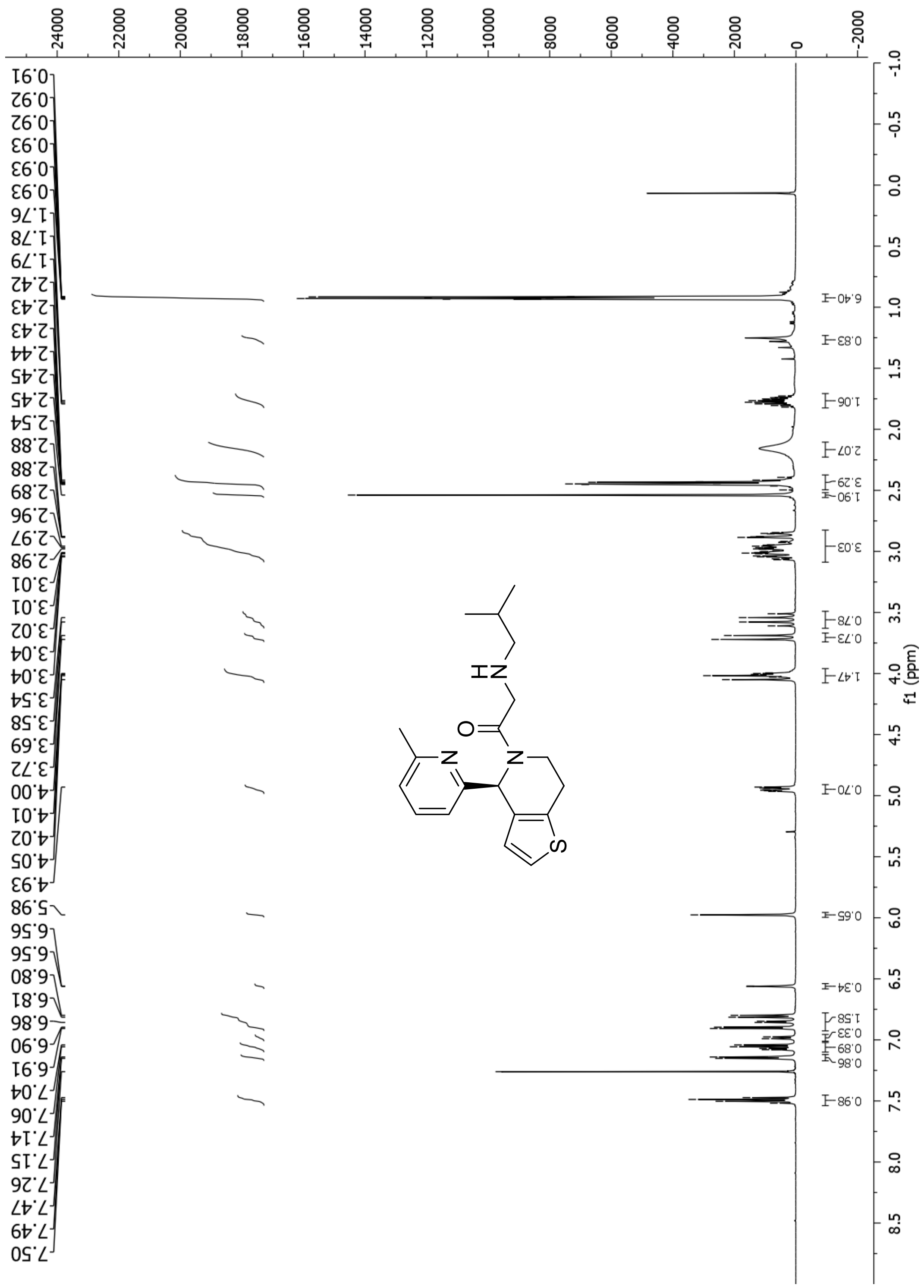

Figure S19. ^{1}H NMR (500 MHz, $CDCl_3$, 298 K) of **(-)-6**.

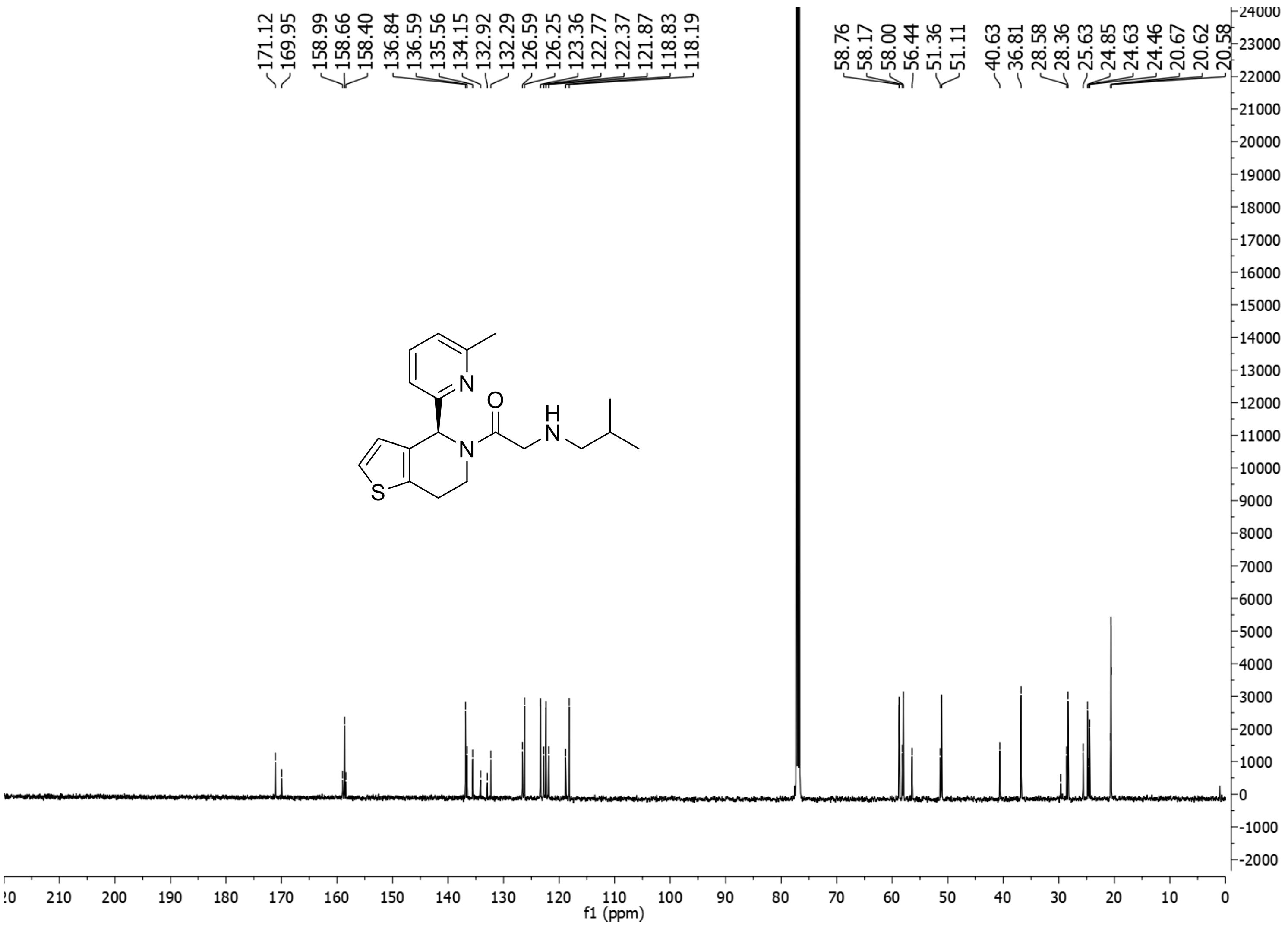

Figure S20. ^{13}C NMR (500 MHz, $CDCl_3$, 298 K) of **(-)-6**.

1.6. (*R*)-(+)-2-(2-Methylpropylamino)-1-[4-(6-methylpyridin-2-yl)-6,7-dihydro-4*H*-thieno[3,2-*c*]pyridin-5-yl]ethanone [(+)-6, IMP-1575]

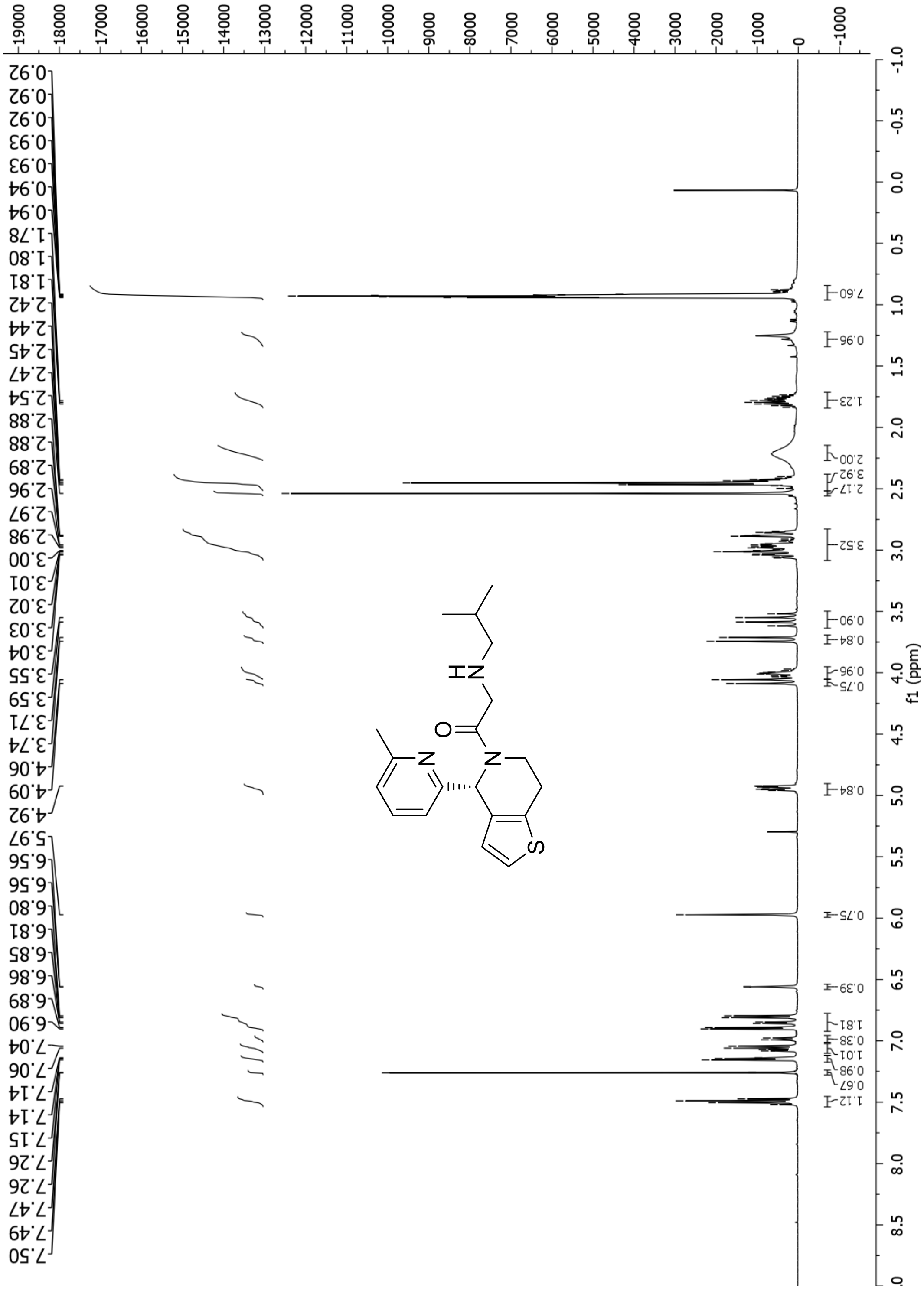

Figure S21. ^{1}H NMR (500 MHz, $CDCl_3$, 298 K) of **(+)-6** (**IMP-1575**).

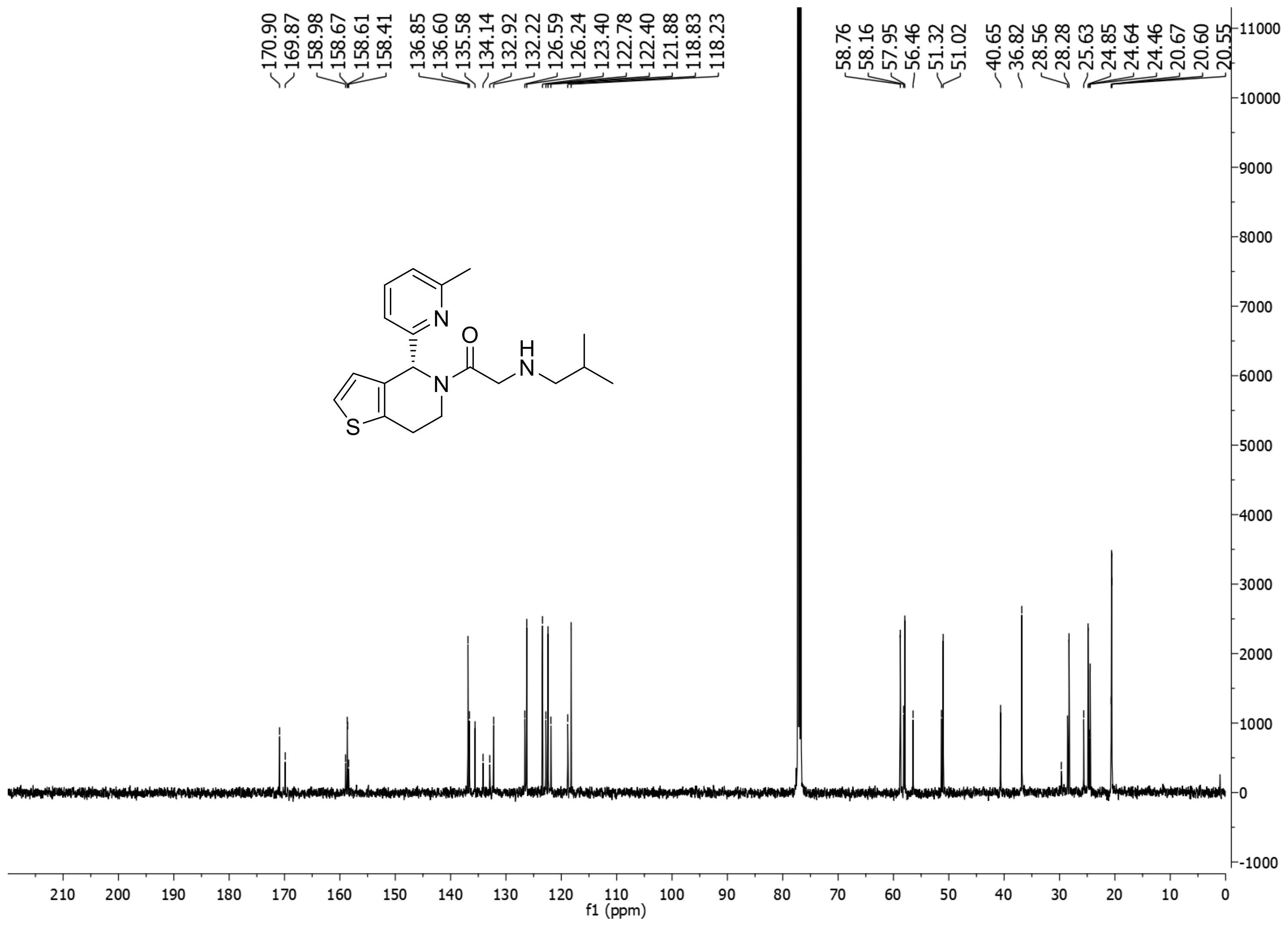

Figure S22. ^{13}C NMR (500 MHz, $CDCl_3$, 298 K) of **(+)-6** (**IMP-1575**)

1.7. 2-(Isobutyl(methyl)amino)-1-(4-(6-methylpyridin-2-yl)-6,7-dihydrothieno[3,2-*c*]pyridin-5(4*H*)-yl)ethan-1-one (7)

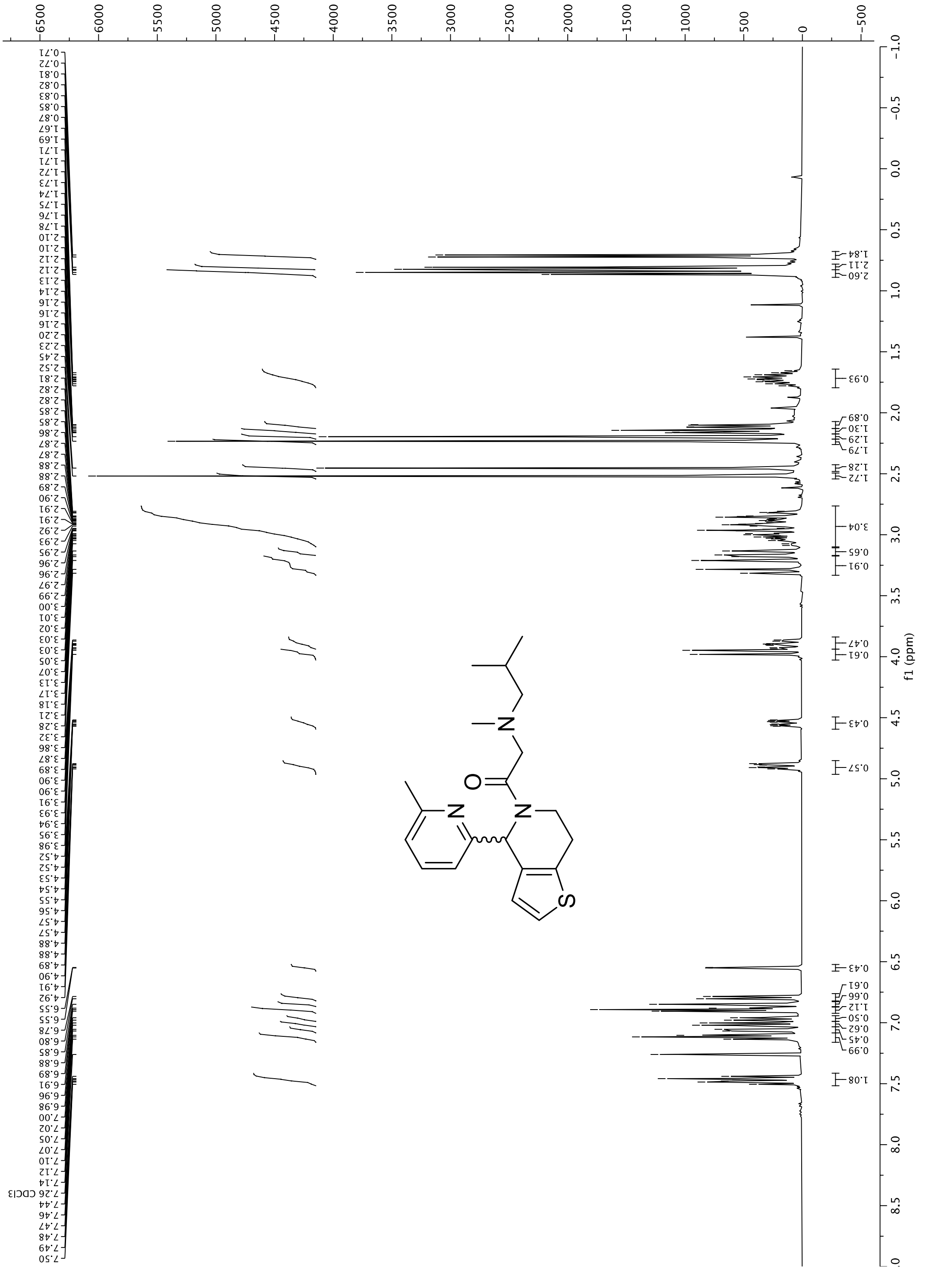

Figure S23. ^{1}H NMR (500 MHz, $CDCl_3$, 298 K) of **7**.

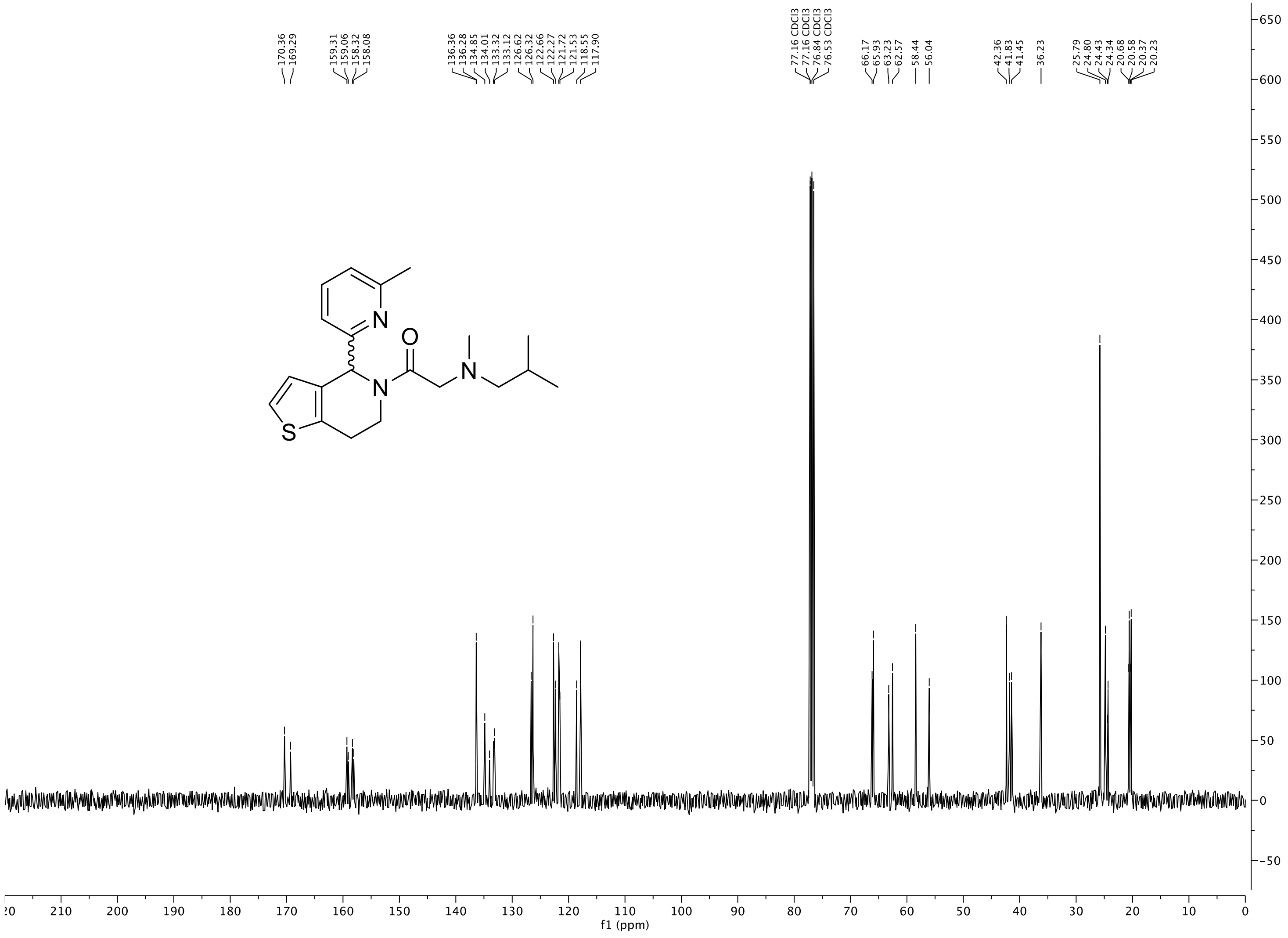

Figure S24. ^{13}C NMR (500 MHz, $CDCl_3$, 298 K) of **7**.

1.8. 2-(3-Methylpiperidin-1-yl)-1-(4-(6-methylpyridin-2-yl)-6,7-dihydrothieno[3,2-*c*]pyridin-5(4*H*)-yl)ethan-1-one (8)

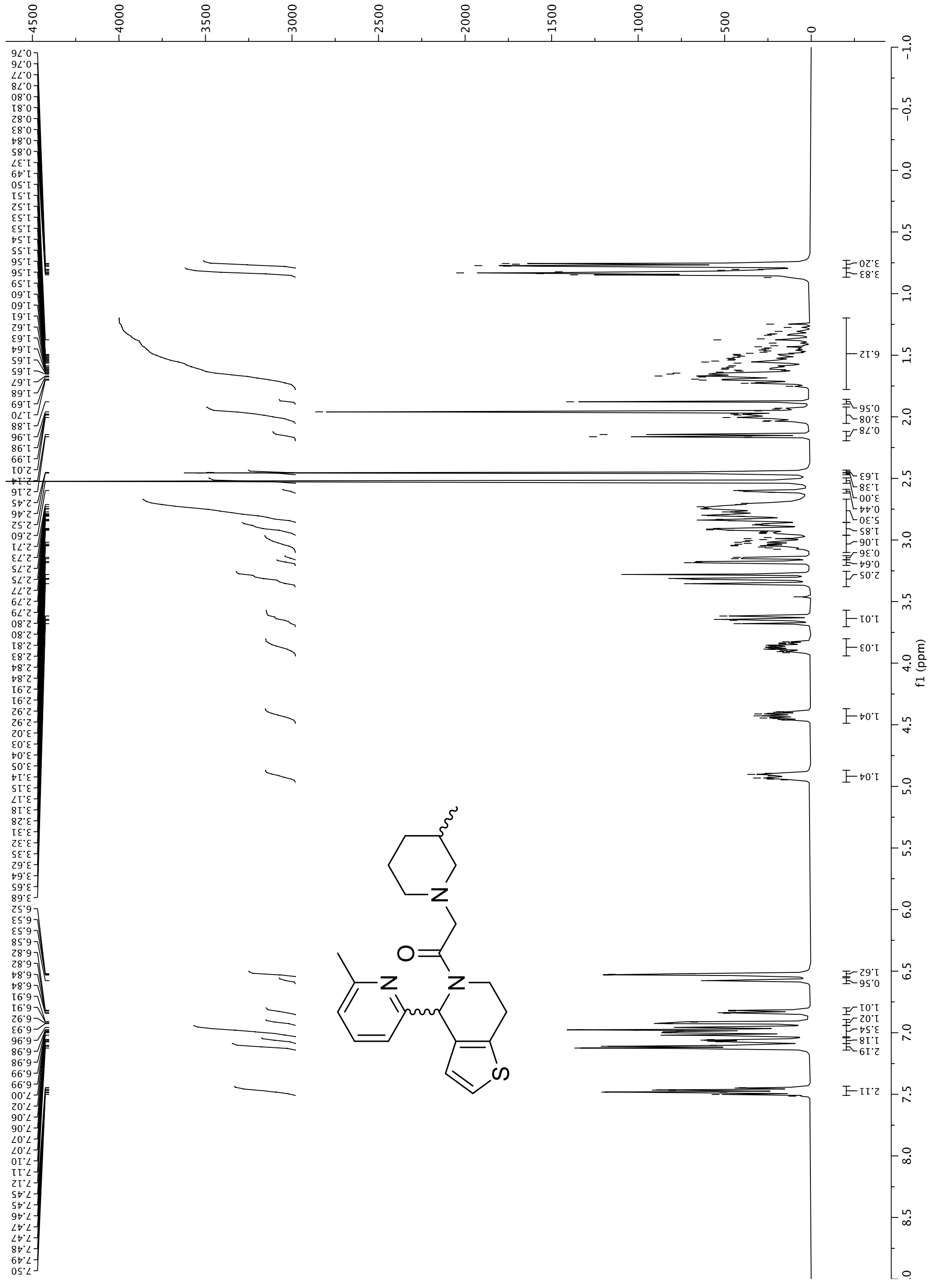

Figure S25. ^{1}H NMR (500 MHz, $CDCl_3$, 298 K) of **8**.

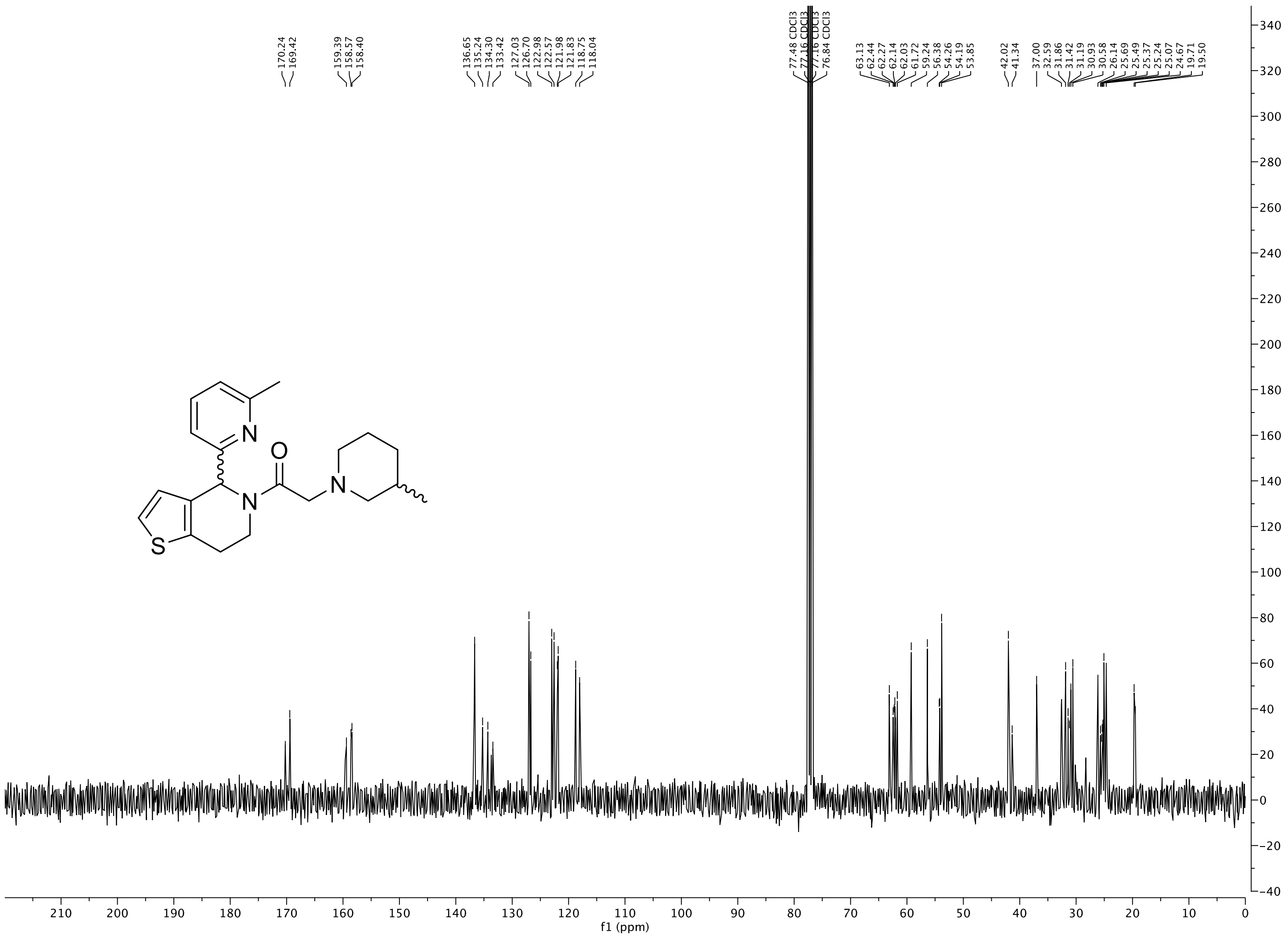

Figure S26. ^{13}C NMR (500 MHz, $CDCl_3$, 298 K) of **8**.

1.9. *N*-isopentyl-4-(6-methylpyridin-2-yl)-6,7-dihydrothieno[3,2-*c*]pyridine-5(4*H*)-carboxamide (9)

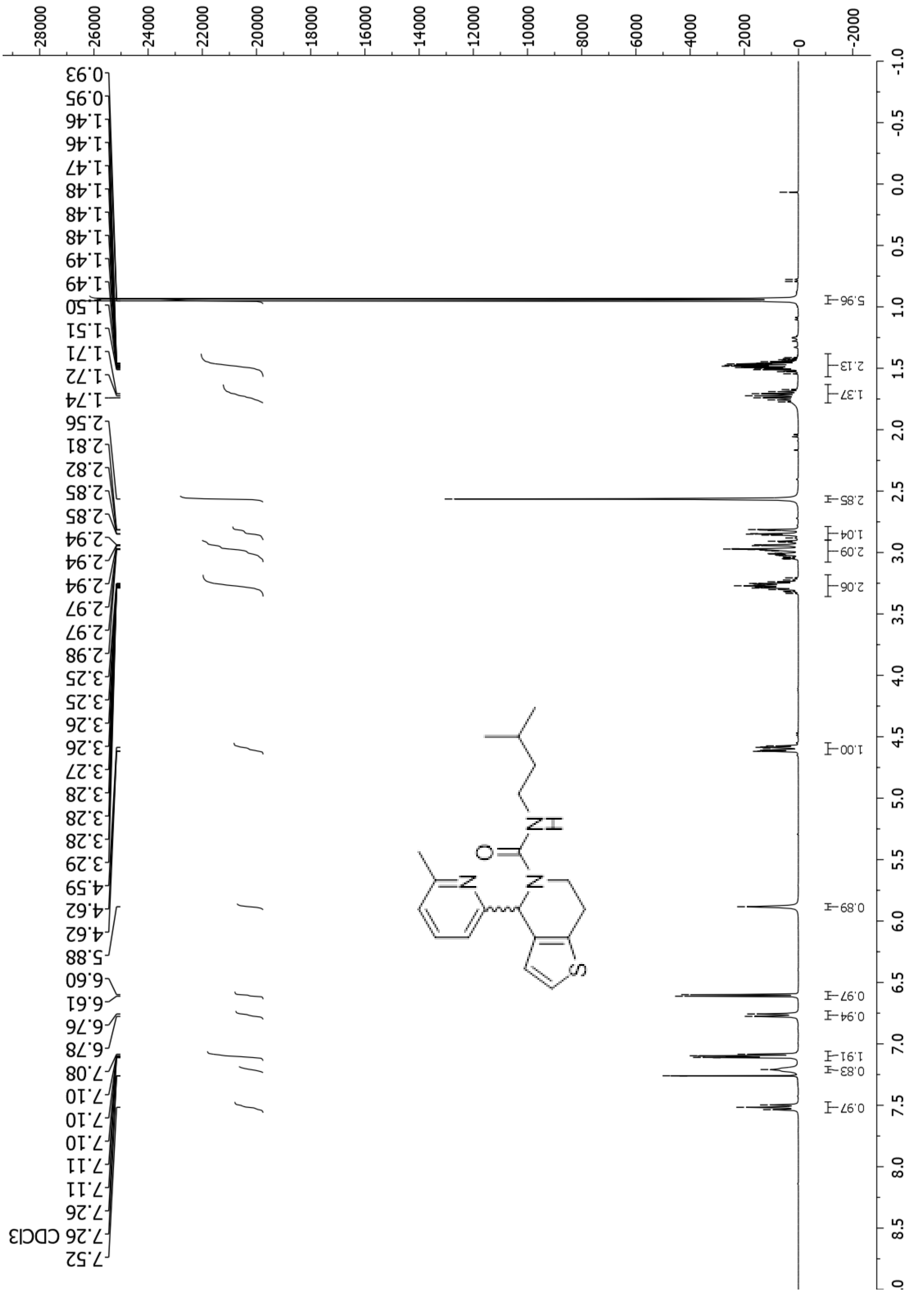

Figure S27. ^{1}H NMR (400 MHz, $CDCl_3$, 298 K) of **9**.

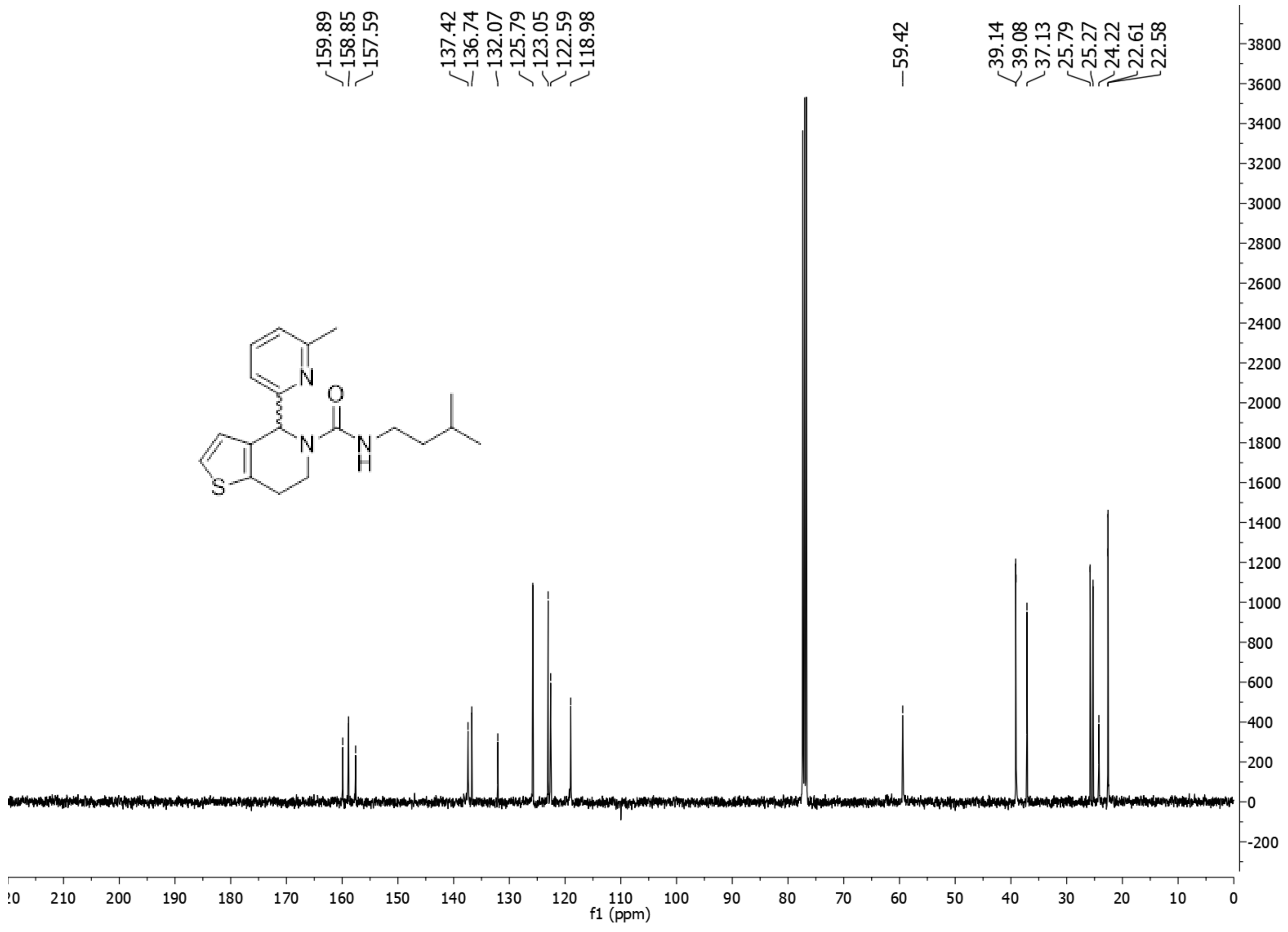

Figure S28. ^{13}C NMR (400 MHz, $CDCl_3$, 298 K) of **9**.

1.10. 2-(3-(But-3-yn-1-yl)-3*H*-diazirin-3-yl)-1-(4-(6-methylpyridin-2-yl)-6,7-dihydrothieno[3,2-*c*]pyridin-5(4*H*)-yl)ethanone (10)

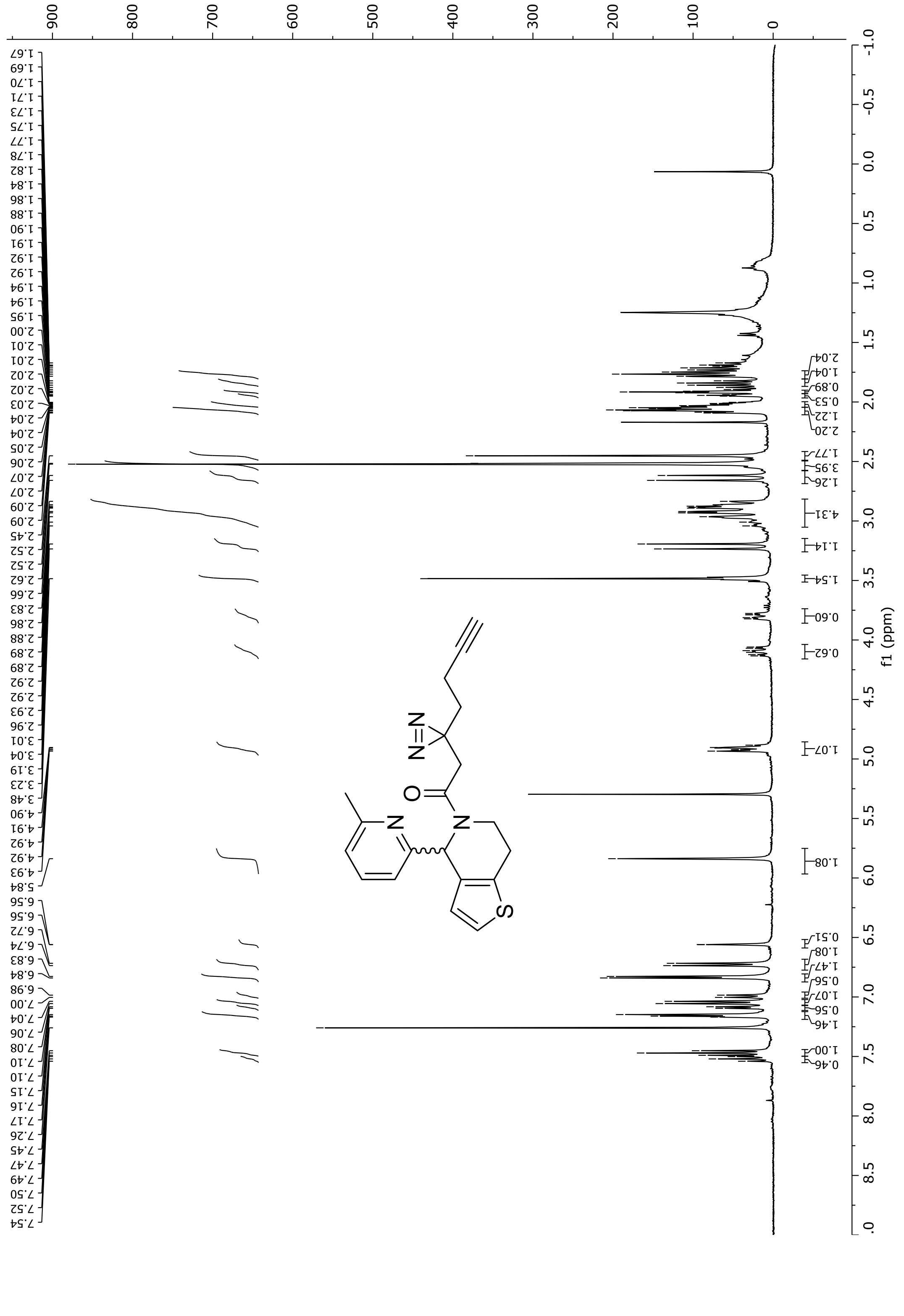

Figure S29. ^{1}H NMR (500 MHz, $CDCl_3$, 298 K) of **10**.

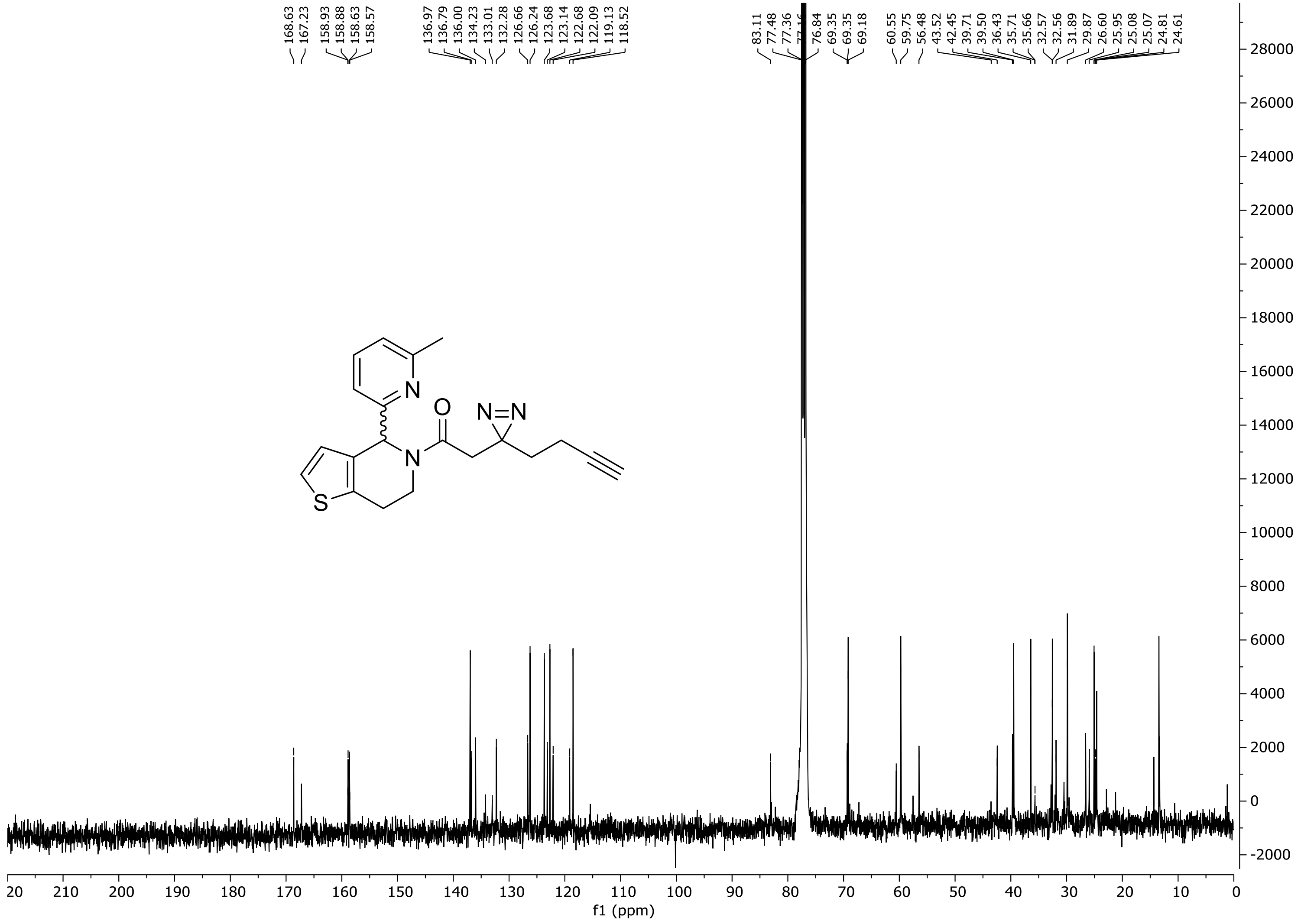

Figure S30. ^{13}C NMR (500 MHz, $CDCl_3$, 298 K) of **10**.

1.11. 3-(3-(But-3-yn-1-yl)-*3H*-diazirin-3-yl)-1-(4-(6-methylpyridin-2-yl)-6,7-dihydrothieno[3,2-*c*]pyridin-5(*4H*)-yl)propan-1-one (11)

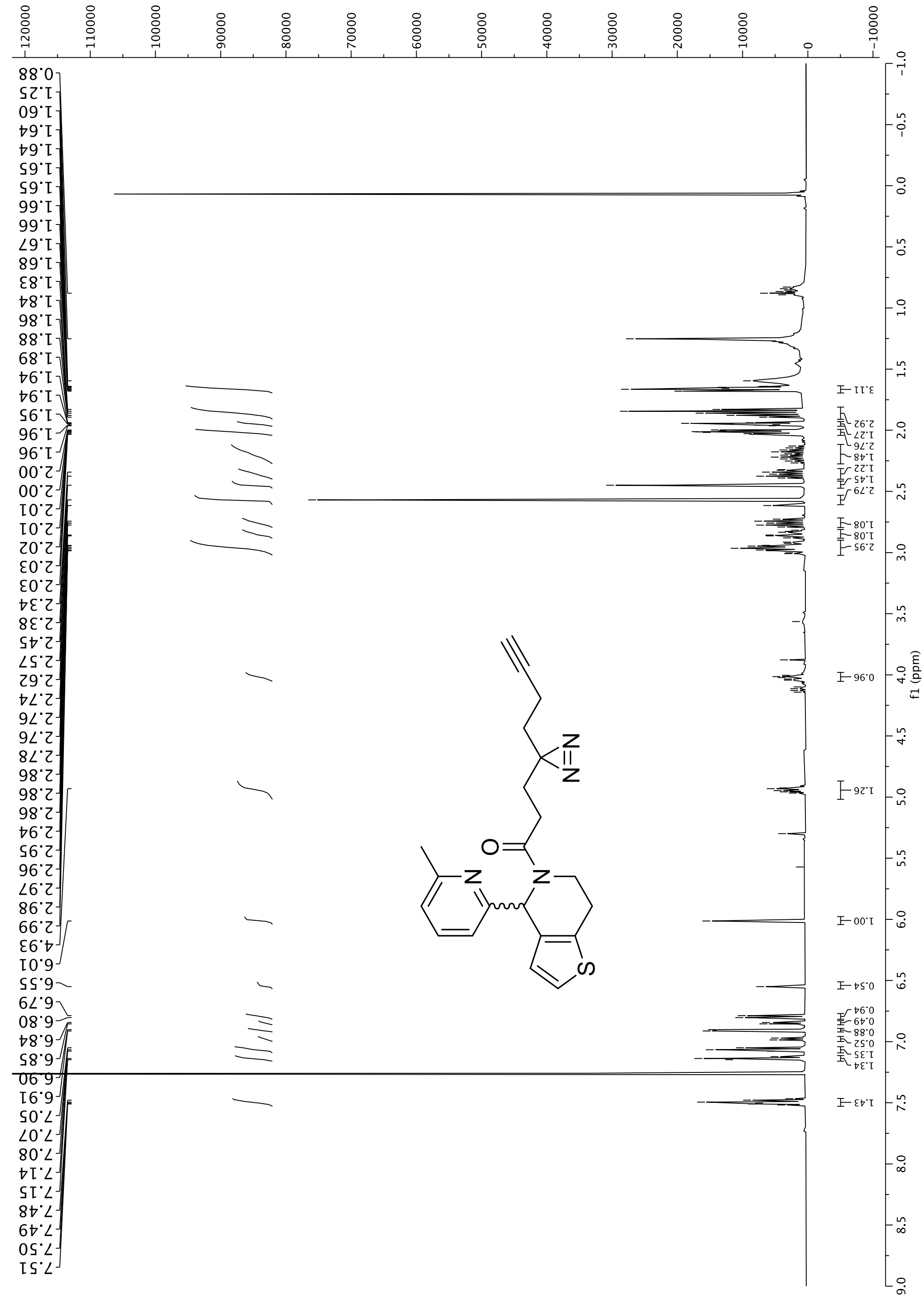

Figure S31. ^{1}H NMR (500 MHz, $CDCl_3$, 298 K) of **11**.

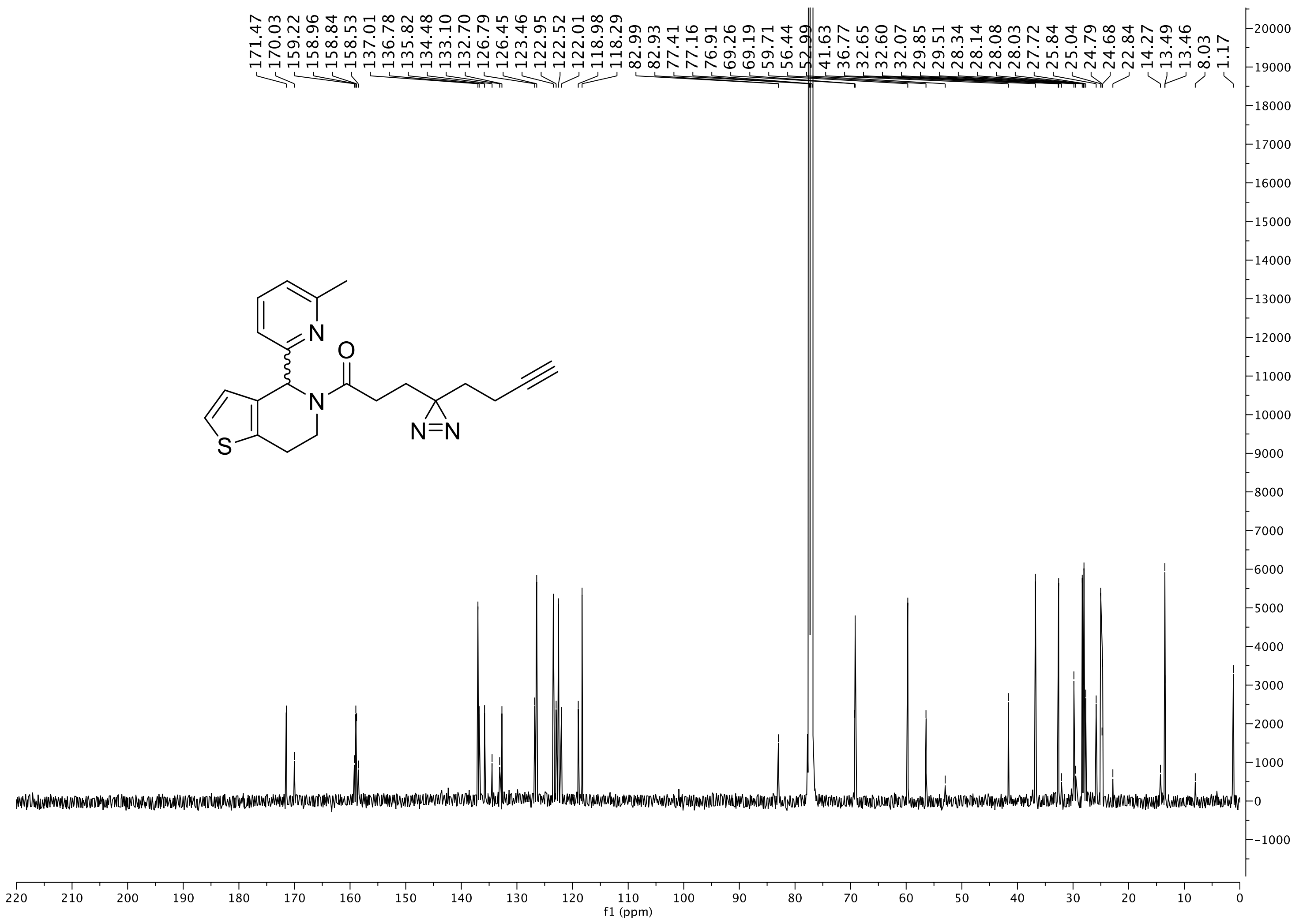

Figure S32. ^{13}C NMR (500 MHz, $CDCl_3$, 298 K) of **11**.

Supplementary References

[1] a) T. Lanyon-Hogg, M. Ritzefeld, N. Masumoto, A. I. Magee, H. S. Rzepa, E. W. Tate, *J. Org. Chem.* **2015**, *80*, 4370-4377; b) T. Lanyon-Hogg, N. Masumoto, G. Bodakh, A. D. Konitsiotis, E. Thinon, U. R. Rodgers, R. J. Owens, A. I. Magee, E. W. Tate, *Data Brief* **2016**, *7*, 257-281.

[2] A. P. M. Ružič, D. Prudič, D. Kralj, C. Scriban, A. Zanotti-Gerosa, *Org. Process Res. Dev.* **2012**, *16*, 1293-1300.

[3] Z. Wu, M. Perez, M. Scalone, T. Ayad, V. Ratovelomanana-Vidal, *Angew. Chem. Int. Ed. Engl.* **2013**, *52*, 4925-4928.

[4] Z. Li, P. Hao, L. Li, C. Y. Tan, X. Cheng, G. Y. Chen, S. K. Sze, H. M. Shen, S. Q. Yao, *Angew. Chem. Int. Ed. Engl.* **2013**, *52*, 8551-8556.

[5] P. Kleiner, W. Heydenreuter, M. Stahl, V. S. Korotkov, S. A. Sieber, *Angew. Chem. Int. Ed. Engl.* **2017**, *56*, 1396-1401.

[6] a) Y. Feng, J. Liu, Y. P. Carrasco, J. B. MacMillan, J. K. De Brabander, *J. Am. Chem. Soc.* **2016**, *138*, 7130-7142; b) C. G. Parker, et al., *Cell* **2017**, *168*, 527-541 e529.

[7] P. Shieh, V. T. Dien, B. J. Beahm, J. M. Castellano, T. Wyss-Coray, C. R. Bertozzi, *J. Am. Chem. Soc.* **2015**, *137*, 7145-7151.

[8] T. Lanyon-Hogg, N. V. Patel, M. Ritzefeld, K. J. Boxall, R. Burke, J. Blagg, A. I. Magee, E. W. Tate, *Slas Discov* **2017**, *22*, 418-424.

[9] T. Lanyon-Hogg, et al., *Chem. Sci.* **2019**, *10*, 8995-9000.

[10] R. A. Copeland, *Methods Biochem. Anal.* **2005**, *46*, 1-265.

[11] M. J. Frisch, et al., Wallingford, CT, **2016**.

[12] a) F. Weigend, R. Ahlrichs, *Phys. Chem. Chem. Phys.* **2005**, *7*, 3297-3305; b) F. Weigend, *Phys. Chem. Chem. Phys.* **2006**, *8*, 1057-1065; c) A. V. Marenich, C. J. Cramer, D. G. Truhlar, *J. Phys. Chem. B* **2009**, *113*, 6378-6396; d) U. Ekstrom, L. Visscher, R. Bast, A. J. Thorvaldsen, K. Ruud, *J Chem Theory Comput* **2010**, *6*, 1971-1980.

[13] a) Avogadro: an open-source molecular builder and visualization tool. Version 1.20. http://avogadro.cc/; b) M. D. Hanwell, D. E. Curtis, D. C. Lonie, T. Vandermeersch, E. Zurek, G. R. Hutchison, *J Cheminform* **2012**, *4*, 17.

[14] T. Bruhn, A. Schaumloffel, Y. Hemberger, G. Bringmann, *Chirality* **2013**, *25*, 243-249.

[15] T. Cheeseright, M. Mackey, S. Rose, A. Vinter, *J Chem Inf Model* **2006**, *46*, 665-676.

[16] J. Rappsilber, Y. Ishihama, M. Mann, *Anal. Chem.* **2003**, *75*, 663-670.

[17] D. Ma, Z. Z. Wang, C. N. Merrikh, K. S. Lang, P. L. Lu, X. Li, H. Merrikh, Z. H. Rao, W. Q. Xu, *Nature* **2018**, *562*, 286-290.

[18] a) A. D. Konitsiotis, B. Jovanovic, P. Ciepla, M. Spitaler, T. Lanyon-Hogg, E. W. Tate, A. I. Magee, *J. Biol. Chem.* **2015**, *290*, 3293-3307; b) A. Matevossian, M. D. Resh, *J. Biol. Chem.* **2015**, *290*, 2235-2243.
